# Supplementary material for: Dating ancient manuscripts using radiocarbon and AI-based writing style analysis
Source: PLoS One. 2025 Jun 4;20(6):e0323185. doi: 10.1371/journal.pone.0323185 (PMC12136314; doi:10.1371/journal.pone.0323185)
Supplement: S1 Appendix — (PDF) [file pone.0323185.s001.pdf]

# **S1 Appendix** for the article:

# Dating ancient manuscripts using radiocarbon and AI-based writing style analysis

Mladen Popović[1*], Maruf A. Dhali[1,2], Lambert Schomaker[2], Johannes van der Plicht[3], Kaare Lund Rasmussen[4], Jacopo La Nasa[5], Ilaria Degano[5], Maria Perla Colombini[5], Eibert Tigchelaar[6],

**1** Qumran Institute, University of Groningen, 9712 GK, The Netherlands
**2** Artificial Intelligence, Bernoulli Institute, University of Groningen, 9747 AG, The Netherlands
**3** Center for Isotope Research, University of Groningen, 9747 AG, The Netherlands
**4** Department of Physics, Chemistry, and Pharmacy, University of Southern Denmark, DK 5230, Denmark
**5** Department of Chemistry and Industrial Chemistry, University of Pisa, 56126 Pisa PL, Italy
**6** Faculty of Theology and Religious Studies, KU Leuven, 3000 Leuven, Belgium

* m.popovic@rug.nl

**Data and materials:** All data, code, and test film associated with this article are publicly available on Zenodo with the following DOIs:

- Data and prediction plots (v3): https://doi.org/10.5281/zenodo.10998958.
- Code and feature files (v6): https://doi.org/10.5281/zenodo.13319794.
- Film (see details in S7 Appendix: https://doi.org/10.5281/zenodo.8167946.

Please note that this article has 12 appendices in total, from **S1** to **S12**.

# S1 The dating problem of the Dead Sea Scrolls

There is broad agreement in scholarship about the long-term lines of development of Aramaic and Hebrew script in Judaea from the fourth century BCE until the second century CE as an evolution from imperial Aramaic chancery script of the fourth century BCE to what became the dominant Jewish square script in the first and second centuries CE. However, when we zoom into the specifics of the centuries in between, the finer typological and chronological distinctions—misleadingly connected with historical-political eras—are not reliably grounded in the data; rather, they rely on so-called absolute pegs that are not absolute at all and on unsubstantiated suppositions about historical processes that would have influenced palaeographic developments.

The main problem is that there is a palaeographic gap between the third century BCE and the second century CE. There is a lack of absolute dates across the time period of the scrolls.

## S1.1 Too few date-bearing manuscripts to compare with

Palaeographic comparison of undated and dated manuscripts with a similar script is not possible. Only few date-bearing manuscripts have survived and those are at the outer limits of the date range. The oldest, from fourth-century BCE Wadi Daliyeh [1] and fourth-century BCE Bactria [2], have script comparable to only one or two manuscripts, 4Q52 and 4Q70 (see also appendix S1.2.2 and S4.1.1, but not the vast majority of the scrolls. The manuscripts from fifth-century BCE Elephantine are even further away in time [3].

The youngest, from first- and second-century CE Murabbaʿat [4, 5] and Naḥal Ḥever [4, 6], are mostly in cursive script and cannot be used to compare and date the vast majority of the Hebrew and Aramaic scrolls written in more formal scripts. Those dated manuscripts include about 30 documentary texts, mainly from Murabbaʿat and Naḥal Ḥever. From the same period are 15 undated but datable letters, mostly in cursive script, to and from Simon bar Kokhba, the leader of the revolt against the Romans in 132–135 CE. Dated documents written in formal or bookhand script are limited to a farming contract from Murabbaʿat (Mur24) and three leases of land from Naḥal Ḥever (5/6Ḥev 44, 45, 46), from 133 and 134 CE.

Only one dated ostracon, from 176 BCE from Maresha [7] is known from the crucial period between the third century BCE and the first century CE. Another ostracon, from Khirbet el-Qom, is partially dated, and could date from 277, 241, or 217/6 BCE [8]. Yet, these can hardly be used for dating formal hands, and cannot even serve as an indicative time marker to tie in manuscripts with a semicursive handwriting.

## S1.2 Weak workarounds

The way taken by Cross and others around the lack of date-bearing documents in formal, semiformal, or semicursive script from the third century BCE until the first century CE does not solve the problem. The relative development and absolute chronology of the scrolls' palaeography was determined by taking recourse to a combination of a. supposed absolute pegs and b. unsubstantiated palaeographic and historical suppositions:

### S1.2.1 Not so absolute time markers

Cross [9] claimed that his model was pegged by a series of absolute datings, in scores if not hundreds of documents inscribed on a variety of materials, especially from the late first century BCE and first century CE. Puech [10] provided additional pegs, specifically for the less formal Hasmonaean hands. Cross and Puech relied on inscriptions on other surfaces such as stone and metal, but here too there are no absolute dates, not even for the most important pegs, such as the Benei Ḥezir tomb and the Jason's tomb inscriptions. Avigad [11] acknowledged this, but his caution seems to have been forgotten.

A telling example is the estimated date of the Benei Ḥezir tomb inscription in Jerusalem's Kidron Valley (CIIP 137 [12]), which, according to Cross, had been dated securely, on the basis of archaeological

and historical evidence, to the end of the first century BCE. Based on architectural typology of the Hellenistic-style façade and Josephus's description of the Maccabees' family tomb in Modiʿin, Avigad [13] initially suggested to date the tomb to the mid-second century BCE. He then estimated the inscription, which lists eight priests from two generations who had been interred in the tomb, to have been made on the façade one or two generations after the construction of the tomb, in the first half of the first century BCE. Later, he dated the inscription palaeographically to the second half of the first century BCE, or to the Herodian period, and on that basis redated the tomb to the end of the Hasmonaean period [11]. The precise length of time between the construction of the façade and the writing of the inscription (how many years are one to two generations?) is a conjecture.

After the 2000–2001 exploration of the Benei Ḥezir and Zechariah tombs, Barag [14] put forward new data and interpretations which would indicate that the tomb dated to the period of flourishing in Jerusalem between ca. 132/1 and 63 BCE, most likely in the first century BCE. For example, it features the new type of tombs typical of the Hasmonaean period, which became common in the first century BCE. In the same direction point correspondences with Nabataean tomb architecture (undated but supposed to go back to the first century BCE), which, Barag argued, likely inspired the Benei Ḥezir tomb. As for the inscription, which he conjectured to be 50–100 years younger than the construction of the tomb, he compared its writing to that of the bronze coins of the 25th year of Alexander Jannaeus (79/8) BCE, and posited that the script of the Benei Ḥezir inscription would seem to be slightly later, from the late Hasmonaean or early Herodian period.

Without mentioning the Benei Ḥezir inscription, Naveh [15] had identified the script on the Alexander Jannaeus coinage as 'vulgar semiformal' and saw its closest parallels to the letters found on ossuaries. Cross [16] had described this style as a "crude simplified derivative" of the formal Herodian hand. Naveh's aligning of the letters of the coins with those of the ossuaries might suggest that this type of Herodian hand was already anticipated by the Jannaeus coins. Naveh therefore referred to the palaeographical significance of these coins. One should note, however, that neither Naveh nor Barag carefully analysed the letters of the coins.

Summarizing, all scholars associate the Benei Ḥezir tomb with the Maccabaees or the Hasmonaean period (either early or late), and date its inscription to the first century BCE. Yet, Cross's claim that a late first-century BCE date is secure and an absolute peg, cannot be sustained. The date estimates of the tomb and its inscriptions are not only based on architectural typology, but also on the palaeographic typology. None of the evidence argues against a mid-first century BCE or even earlier date of the inscription.

This is one example to demonstrate that inscriptions in Hebrew and Aramaic on other surfaces, such as stone and metal, cannot fill the void of absolute dating pegs between the third century BCE until the first century CE. In addition to the Benei Ḥezir burial inscription, this applies also, for example, to the so-called Queen Helena inscription (CIIP 123 [12]) and Uzziah plaque (CIIP 602 [12]) from the first century CE. Strictly speaking, these are not absolutely dated. The same applies to the Jason's tomb inscriptions (CIIP 392-397 [12]), the date of which is not fixed either. Puech [17, 18] had initially argued on the basis of his reconstruction of the historical background of the inscriptions that the main one in Aramaic (CIIP 392) must be dated to 82/1 BCE, but more recently he stated that the Aramaic inscription dates palaeographically to about the middle of the first century BCE or slightly earlier [10]. Yet, Yardeni dated the inscription shortly before the destruction of the tomb by an earthquake in 31 BCE [12].

Another example are the hundreds of ossuary inscriptions, which Cross [19] said to virtually all belong to the Herodian era. A post-20/15 BCE date for the ossuaries may be archaeologically correct [20], but the political and historical framing to the Herodian period does not limit the emergence of the script exhibited on the ossuaries to that period. The question when the so-called Herodian script came into being is decided somewhat arbitrarily. Cross took 30 BCE, Milik and Baillet 50 BCE [21]. Avigad also took 50 BCE or slightly earlier. Furthermore, Avigad already acknowledged that scrolls referred to as 'Herodian' may easily be earlier than this period [11]. In other words, even for the 'Herodian' script, just as for the 'Hasmonaean' script (see below), the emergence is difficult to establish. In terms of typological development, we have to reckon with the possibility of longer, broader time frames for both scripts.

### S1.2.2 Unsubstantiated palaeographic and historical premises

Even if one were to accept Cross's recourse to a series of absolute datings, these would support mainly late first-century BCE and first-century CE comparisons. They do not help to establish the beginnings of the 'Hasmonaean' script. Lacking dated material from the third and second centuries BCE, Cross had to take further recourse to two premises to attempt to establish the upper range of the oldest scripts, 'Archaic' and 'Hasmonaean', from the scrolls, and to limit the earliest dating of the scrolls mainly to the second century BCE, with only a few exceptions for older 'Archaic' manuscripts such as 4Q52 and 4Q70.

In addition to a lack of time markers, two palaeographic and historical premises by Cross, Yardeni and others stand out: a slow development of the Aramaic/Hebrew script in the early Hellenistic period (third century BCE); and the emergence of a national script as a watershed around 200–150 BCE.

The presumed slow development of the Aramaic/Hebrew script in the early Hellenistic period is not supported by any dated evidence of that period. The assumption was in part based on a few undated cursive Aramaic papyri from Egypt containing Greek names (hence assumed to be from the third century BCE), but the later discovery of the dated Wadi Daliyeh papyri showed that there were different lines of development, some having taken place much earlier [22, 23], thus challenging the premise of the slow development, and reducing the importance of those Hellenistic Egyptian Aramaic papyri for establishing the evolution of the Aramaic/Hebrew script. For Judaea, Cross [19] also referred in passing to a conservative palaeography for the copying of sacred texts, but without further explanation or supporting evidence.

Cross initially dated 4Q52 (4QSam$^{b}$) to "the last quarter of the third century B.C." [19], "no doubt late in the century" [24], but after the discovery of the Wadi Daliyeh manuscripts, simply to "ca. 250 B.C." [22] or "the mid-third century BCE" [9, 25]. He seems to have been reluctant to date 4Q52 and also 4Q70 (4QJer$^{a}$) earlier, and therefore assumed a very slow evolution of the script, so as not to have a large time gap with the manuscripts written in what he called the "early Hasmonaean" script and which he dated to ca. 150 BCE.

Yardeni, too, regarded 4Q52 and 4Q70 as examples of a transitional stage from the fourth and third century BCE Aramaic script in the direction of the 'Hasmonaean' script [23]. Her conclusion that these two manuscripts could therefore be dated to the late third or early second century BCE seems to be based rather on the supposed proximity to this national script than on the correspondences with the earlier Aramaic scripts.

However, the palaeographic principle is to date an undated manuscript by comparing its script to that of dated writings with a similar script. This means that the oldest manuscript of the scrolls, 4Q52, must be compared to the Aramaic evidence from Wadi Daliyeh from the fourth century BCE. 4Q52 should then be chronologically closer to those manuscripts, especially WDSP 1 (335 BCE).

The hypothesis of the emergence of a national script around 200–150 BCE and the supposition that the 'Hasmonaean' script was a development of the Hasmonaean period after 150 BCE are not supported by any dated evidence but based on historical assumptions, given in passing, about a "nationalistic expansion and resurgent Orientalism" [9] after the death of the Seleucid king Antiochus IV Epiphanes (164 BCE). These unfounded assumptions were then imposed as an interpretative framework on the manuscript evidence. But, given the absence of dated material from the third and second century BCE, there are no historical, typological or other palaeographic reasons for limiting the rise of the script which Cross called 'Hasmonaean' to the mid-second century BCE.

This means that manuscripts written in 'Hasmonaean' script may date already from the early second century or from the third century BCE. This older dating is also realistic when manuscripts written in so-called 'Archaic' script, such as 4Q52 or 4Q70, can be dated earlier in the third century BCE or, for 4Q52, even perhaps in the late fourth century BCE. Furthermore, this older dating can be independently supported by the $^{14}$C dating results in this study (see appendix S4.1).

## S1.3 The way out of the gap

Summarizing, the dating problem of the Dead Sea Scrolls, due to the absence of calendar dates, is further confounded by the fact that there are no other date-bearing manuscripts in similar script available for

palaeographic comparison. This lack of date-bearing documents cannot be overcome by using inscriptions on other surfaces instead because these, too, have no absolute dates. Also, datable inscriptions mainly date from the first century BCE and first century CE and thus cannot shed light on script developments in the third and second centuries BCE. Historical premises and assumptions remain unsubstantiated and devoid of factual support, and they fail to support a chronological framework for the palaeography and the manuscript evidence. These assumptions cannot determine or sufficiently constrain the dates connected to the writing of the scrolls.

Therefore, $^{14}$C dates derived from manuscript samples are needed as absolute time markers to lead the way out of the palaeographic gap. In the absence of an abundance of date-bearing manuscripts written in similar script available for palaeographic comparison, $^{14}$C dating, a scientific measurement ("yardstick of time"), provides more reliable time markers, and in combination with our style-based date-prediction model Enoch even more precise time markers.

There is the theoretical possibility that there might be a chronological gap between the writing material and the act of writing, i.e., scribes could use blank papyrus or leather sheets that had been prepared some time beforehand. This would lead to a time difference between the scientific dating of the artefact and the presumed date of writing. However, we do not know of archaeological, documentary or literary evidence from antiquity that demonstrates it was standard practice to use writing material that was prepared decades before the text was written upon it. Therefore, we assume that in most cases the text written on a manuscript is the same year or very close in years to the preparation of the writing material so that $^{14}$C dating is the best way to date the handwriting in the current circumstances.

## References


1. Gropp DM. Discoveries in the judaean desert: Volume XXVIII. Wadi daliyeh II and Qumran miscellanea, part 2. Oxford: Clarendon Press; 2001.

2. Naveh J, Shaked S. Aramaic documents from ancient Bactria (Fourth Century BCE.). London: The Khalili Family Trust; 2012.

3. Porten B, Yardeni A. Textbook of Aramaic documents from ancient Egypt, 4 vols. Jerusalem: The Hebrew University; 1986–1999.

4. Yardeni A. Textbook of Aramaic, Hebrew and nabataean documentary texts from the judaean desert and related material, 2 vols. Jerusalem: The Hebrew University; 2000.

5. Benoit P, Milik JT, de Vaux R. Discoveries in the Judaean Desert: Volume II. Les grottes de Murabbaʿât, 2 Vols. Oxford: Clarendon Press; 1961.

6. Cotton HM, Yardeni A. Discoveries in the Judaean Desert: Volume XXVII. Aramaic, Hebrew and Greek documentary texts from Naḥal Ḥever and other sites. With an appendix containing alleged Qumran texts. Oxford: Clarendon Press; 1997.

7. Eshel E, Kloner A. An Aramaic Ostracon of an Edomite Marriage Contract from Maresha, Dated 176 BCE. Israel Exploration Journal. 1996;46:1–22.

8. Geraty LT. The Khirbet el-Kôm bilingual ostracon. Bulletin of the American Schools of Oriental Research. 1975;220:55–61.

9. Cross FM. The Development of the Jewish Scripts. In: Leaves from an Epigrapher's Notebook: Collected Papers in Hebrew and West Semitic Palaeography and Epigraphy. Winona Lake, IN: Eisenbrauns; 2003. p. 1–43.

10. Puech E. La paléographie des manuscrits de la mer Morte. In: Fidanzio M, editor. The Caves of Qumran. Leiden: Brill; 2017. p. 96–105.

11. Avigad N. The Palaeography of the Dead Sea Scrolls and Related Documents. In: Scripta Hierosolymitana, Volume IV: Aspects of the Dead Sea Scrolls. Jerusalem: Magness Press; 1965. p. 56–87.

12. Eck W, Cotton HM, Di Segni L. Corpus Inscriptionum Iudaeae/Palaestinae: Volume 1, Part 1. vol. 1. Berlin: De Gruyter; 2010.

13. Avigad N. Ancient Monuments in the Kidron Valley. Jerusalem: Bialik Institute; 1954.

14. Barag D. The 2000-2001 exploration of the tombs of Benei Ḥezir and Zechariah. Israel Exploration Journal. 2003;53:78–110.

15. Naveh J. Dated Coins of Alexander Janneus. Israel Exploration Journal. 1968;18:20–26.

16. Baillet M, Milik JT, de Vaux R. Discoveries in the Judaean Desert of Jordan: Volume III. Les 'petites grottes' de Qumrân, 2 Vols. Oxford: Clarendon Press; 1962.

17. Puech E. Discoveries in the Judaean Desert: Volume XXXI. Qumrân Grotte 4.XXII. Textes araméens, première partie: 4Q529-549. Oxford: Clarendon Press; 2001.

18. Puech E. Inscriptions funéraires palestiniennes: Tombeau de Jason et ossuaires. Revue biblique. 1983;90:481—-533.

19. Cross FM. The oldest manuscripts from Qumran. Journal of Biblical Literature. 1955;74:147–172.

20. Magness J. Ossuaries and the Burials of Jesus and James. Journal of Biblical Literature. 2005;124:121–154.

21. Tigchelaar E. Seventy Years of Palaeographic Dating of the Dead Sea Scrolls. In: Drawnel H, editor. Sacred Texts and Disparate Interpretations: Qumran Manuscripts Seventy Years Later. Leiden: Brill; 2020. p. 258–278.

22. Cross FM. The Papyri and Their Historical Implications. In: Lapp PW, Lapp NL, editors. Discoveries in the Wâdī ed-Dâliyeh. Cambridge, MA: American Schools of Oriental Research; 1974. p. 17–29.

23. Yardeni A. The Palaeography of 4QJer$^{a}$ – A Comparative Study. Textus. 1990;15:233–268. doi:10.1163/2589255x-01501012.

24. Cross FM. The development of the Jewish scripts. In: Wright GE, editor. The bible and the ancient near east. Garden City, NY: Doubleday; 1961. p. 133–202.

25. Cross FM. Palaeography and the Dead Sea Scrolls. In: Flint PW, VanderKam JC, editors. The Dead Sea scrolls after fifty years: A comprehensive assessment, Volume one. Leiden: Brill; 1998. p. 379–402.
