# Supplementary material for: Dating ancient manuscripts using radiocarbon and AI-based writing style analysis
Source: PLoS One. 2025 Jun 4;20(6):e0323185. doi: 10.1371/journal.pone.0323185 (PMC12136314; doi:10.1371/journal.pone.0323185)
Supplement: S2 Appendix — (PDF) [file pone.0323185.s002.pdf]

S2 Appendix for the article:

Dating ancient manuscripts using radiocarbon and AI-based writing style analysis

Mladen Popović[1*], Maruf A. Dhali[1,2], Lambert Schomaker[2], Johannes van der Plicht[3], Kaare Lund Rasmussen[4], Jacopo La Nasa[5], Ilaria Degano[5], Maria Perla Colombini[5], Eibert Tigchelaar[6],

1 Qumran Institute, University of Groningen, 9712 GK, The Netherlands
2 Artificial Intelligence, Bernoulli Institute, University of Groningen, 9747 AG, The Netherlands
3 Center for Isotope Research, University of Groningen, 9747 AG, The Netherlands
4 Department of Physics, Chemistry, and Pharmacy, University of Southern Denmark, DK 5230, Denmark
5 Department of Chemistry and Industrial Chemistry, University of Pisa, 56126 Pisa PL, Italy
6 Faculty of Theology and Religious Studies, KU Leuven, 3000 Leuven, Belgium

* m.popovic@rug.nl

Data and materials: All data, code, and test film associated with this article are publicly available on Zenodo with the following DOIs:

- Data and prediction plots (v3): https://doi.org/10.5281/zenodo.10998958.
- Code and feature files (v6): https://doi.org/10.5281/zenodo.13319794.
- Film (see details in S7 Appendix: https://doi.org/10.5281/zenodo.8167946.

Please note that this article has 12 appendices in total, from **S1** to **S12**.

S2 Radiocarbon dating of the Dead Sea Scrolls

Two series of Dead Sea Scrolls were radiocarbon dated in the 1990s, in Zurich and in Tucson, Arizona [1–3]. In addition, three samples were submitted to Oxford but in all three cases the chemistry is recorded as having "failed," i.e., no sample to measure; probably the samples completely dissolved during the pretreatment phase (communication from R. Hedges, Research Laboratory for Archaeology, Oxford, 7 January 2005).

Although scrolls were radiocarbon dated in the 1990s, new radiocarbon dating was necessary because of castor oil contamination issues with these previous dates. Furthermore, since then, radiocarbon dating methods and procedures have improved significantly in terms of better calibration, higher precision obtained by more modern methods and instruments, and also more effective cleaning procedures for dealing with contaminated samples.

In this study, we have taken the following analytical steps for the samples:

1. They were precleaned by a Soxhlet procedure in Odense (see appendix S2.2 and S2.7.1);
2. Subsequently, they were further pretreated by standard methods in Groningen (see appendix S2.2);
3. The cleaned samples were dated by Accelerator Mass Spectrometry (AMS) in Groningen (see appendix S2.3–S2.6);
4. During the study, the residual lipids in the extracts of all 30 samples after the Soxhlet cleaning were analysed, and 17 samples have been further investigated by specialized analytical chemistry methods in Pisa regarding the nature of the contamination (see appendix S2.7.2–S2.7.5).

S2.1 Selection of Samples

The 30 samples we received from the Israel Antiquities Authority (IAA) were selected on the basis of script and presumed period so as to obtain reliable time markers in the palaeographic gap between the fourth century BCE and the second century CE. We made this selection at the start of the project on the basis of the default model in the field (see appendix S1). The dates associated with the manuscripts according to this traditional model provided balanced coverage of the timeline under investigation (as can be seen in Fig S1). Also, because the ^{14}C dates are needed to go into the date-prediction model, we selected manuscripts that contain a sufficient number of characters in their extant material, 150–200 [4]. The manuscript identity and presumed palaeographic periods of the samples were not known to the staff of the laboratories in Groningen, Odense, and Pisa at the time of the measurement. One of the 30 samples, from a date-bearing document (Mur19), was added as a control text. Its identity and date were also unknown to the laboratories at the time of measurement. Furthermore, in consultation with the IAA, the final selection of samples was determined also on the basis of practical and conservational considerations regarding specific manuscript remains. The IAA provided general indications concerning where the physical samples were taken from (see appendix S10). In our sample set, we have 28 manuscripts of animal skin, and 2 of papyrus (4Q255/4Q433a and Mur19).

From the first century CE onward, a clear distinction appears in the manuscript evidence between the square bookhand script and the standard cursive style [5], but such a distinction is less pronounced in the manuscript evidence of earlier periods. This also applies to the distinctions made between formal, semiformal, and semicursive styles. Across the continuum of the chronological range covered by the scrolls, exemplary specimens for some styles are lacking [6]. Often manuscripts exhibit a mixture of these presumed styles [7–9]. Therefore, our sampled manuscripts cover all three categories and their mixtures. The cursive style has been excluded from our sampling, except for Mur19 which was used as a validation test for ^{14}C.

S2.2 Soxhlet Treatment and AAA Pretreatment

Castor oil was used in the 1950s by the original team of scholars reconstructing and editing the Dead Sea Scrolls to clean the manuscripts and to improve readability of the text. But the castor oil needs

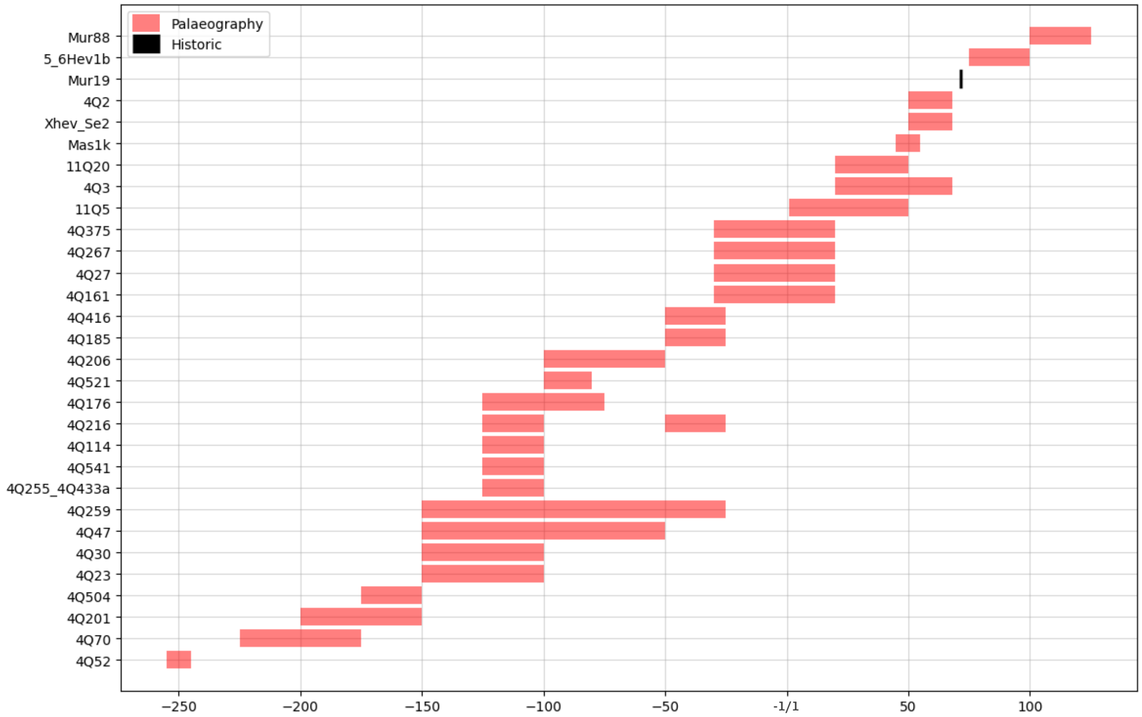

Fig S1. The selection of 30 manuscript samples according to their traditional palaeographic date estimates.

to be removed, because it would give a misleading ^{14}C age that was "younger" than the true age of the sample. Later testing showed that not all castor oil will be removed even by the standard AAA (Acid-Alkali-Acid) protocol, let alone by the reduced form of the standard protocol used in the 1990s in Zurich and Tucson [10–12].

Before the actual start of the project, we received 2 test samples from the IAA which were relatively large (tens of milligrams). These were materials without context but of scrolls origin according to the IAA. Both samples were subjected to the standard AAA treatment, but the material immediately started dissolving before our eyes during the first acid step. This meant we could not apply the standard method; also considering the test samples were much larger than the identified manuscript samples we were to receive. As in the previous attempts made at dating the scrolls [2, 3], we, too, had to adjust the standard chemical AAA (Acid-Alkali-Acid) pretreatment

In our project the first step was to pre-clean the samples by a liquid extraction with suitable solvents, performed inside a Soxhlet apparatus to remove the castor oil contamination. The Soxhlet treatment was carried out in Odense, initially in three but subsequently in four extraction steps. The latter was done for redundancy, and not because of suspicion that the three-step procedure was not sufficient within the given dating uncertainty. The fourth step furthermore guaranteed that even more potential contaminants were removed. Castor oil is a plant product, which consists of several triglycerides and free fatty acids. The Soxhlet treatment is designed to remove lipid material to a high extent, and the analyses done in Pisa by HPLC-MS and Py-GC/MS are performed to demonstrate that the amount of the remaining lipid material, including fatty acids, is below a threshold which does not significantly skew the radiocarbon date (see appendix S2.7).

The scrolls are extremely delicate material. Their fragility is an issue for the chemical cleaning of samples for radiocarbon dating. In the 1990s, the Zurich and Tucson laboratories had to adjust or stop the AAA pretreatment because the samples were dissolving [2, 3]. Most of these samples were much larger than the samples in the current study. With much smaller sample materials, we also had to adjust the standard chemical pretreatment.

Following the Soxhlet treatment in Odense, the samples were further prepared for dating in Groningen.

The pretreatment was adapted to Acid only in a “soft” form: 0.5–1% HCl, refrigerator temperature (ca. 4°C) and only for 10 minutes. Next, we dried the sample in an oven at a temperature of 80°C overnight. Using diluted HCl and skipping the Alkali step is necessary because of the delicate nature of the samples. This is justified because of the conditions the scrolls were kept in. No significant amounts of foreign materials that could cause errors larger than the measurement uncertainties were observed. Our procedure is proven correct because the sample with a known historical date (Mur19) was ^{14}C dated correctly. Combined with the Soxhlet treatment, this is the optimum treatment for this delicate material, and generally effective.

The scrolls were stored in caves in the Judaean desert in the absence of humic acids and constant groundwater. In particular the humic acids constitute a problem for many other archaeological excavations worldwide and they are the main reason that necessitates the alkaline bath in the standard pretreatment protocol (the second A in AAA). The environment in the caves can be characterized as limestone, gypsum and marls — none of which has the potential to inflict alkali-soluble compounds onto the parchments. Similarly, bat guano and excretions from other small animals who have possibly found their way into the caves over the centuries are unlikely to contain humic acids, and therefore their deposits are likely to be dissolvable in either the more polar solvents of the Soxhlet treatment (i.e., the ethanol) or in the acidic bath of the pretreatment in the radiocarbon laboratory. And even further, the pyrolysis-gas-chromatography measurements did not reveal any compounds unaccounted for (see appendix S2.7.4); that includes the alkanes that can be considered markers for bat guano [13].

S2.3 AMS Measurements

After cleaning, the samples were combusted into CO_2 gas. For the GrA dates, the gas is subsequently reduced to graphite using H_2. Subsequently, the ^{14}C content was measured in this graphite. This method was also applied by the GrM machine for routine dating. However, this machine also has the option to measure the ^{14}C content in CO_2, skipping the graphite production step. This is very useful for small samples, as is the case for many scroll samples. Therefore, for scroll samples measured by the Micadas, the gas source was used. For more details on measurement procedures see [14].

For the 30 samples in this study, there is a grand total of 131 individual AMS runs. This total number includes duplicate samples and multiple runs. In most cases a solid date can be calculated for the separate runs done for a particular scroll, based on averaging. The numbers reported reflect the measurements by AMS. In addition, there are aspects of sample integrity and pretreatment which are hard or even impossible to quantify. We have rejected 10 AMS runs for technical reasons, resulting in a final number of 121 valid runs.

The ^{14}C content in the sample is measured by AMS. The original AMS was a 2.5 MV Tandetron accelerator [15]. It was decommissioned in 2017, and replaced by a Micadas system [16]. This took place during the project, so that both machines have been used to date the scroll samples. This allows for internal intercomparison (see Table S1). The Tandetron dates have laboratory code GrA; for the Micadas, this is GrM.

S2.4 AMS Dating Results

Radiocarbon dates are reported by convention in BP, using a defined halflife and reference radioactivity for ^{14}C, and a correction for isotopic fractionation using the stable isotope ^{13}C [17]. The BP dates are converted to calendar dates, using the IntCal20 calibration curve ([18]) and OxCal program [19]. The authors are aware that OxCal 4.4.4 (Web interface build number: 174, last updated: 25/6/2024) is available at the time of publication but we have checked that quantitatively the results do not change since the same calibration curve is used as in 4.4.2. The calibration results in a non-Gaussian probability distribution of calendar dates. This distribution is given in 1σ (68.3% confidence) and 2σ (95.4% confidence) date ranges.

For the 30 samples, 27 yielded valid dates; only 3 samples yielded inconsistent results and had to be technically rejected (4Q216, 11Q20, and Mur88; see appendix S2.6). Also, it appeared that the sample

received for 4Q185 could not be ascertained as belonging to that particular manuscript. This sample is therefore not used in our analysis (see appendix S2.5).

The resulting ^{14}C dates for the 26 samples are shown in Table S1. Each individual ^{14}C sample receives a unique laboratory number. As the table shows, each scroll is dated at least twice. In addition, many measurement batches were repeated (thus yielding two dates per graphite sample). The resulting ^{14}C age shown is the averaged number for all valid runs. Overall, the logistics is complex. For example, the sample 4Q114 (4QDaniel[c]) has been dated in 7 runs. Two samples were received from the IAA. Graphite was prepared from all material of the first sample, and it was dated by the GrA machine. There were 3 runs from the same graphite (to increase the ^{14}C statistics), so all have the same GrA number; the 3 runs are triplicates and can be taken together as 1 GrA date. An additional second sample was received later. From this sample we dated 4 subsamples in 4 runs by the GrM machine. Hence there are 4 GrM numbers.

The resulting BP dates are very precise, with 1σ uncertainties of only 15–28 years. For the full results of all runs with more details (in particular Carbon yield and δ^{13}C value), see appendix S11.

Table S1 shows the summarized results of 26 valid ^{14}C dates: laboratory code, sample identification, ^{14}C age (BP), its sigma (BP), and calibrated dates (both 1σ and 2σ ranges). The OxCal plots can be seen in appendix S3.

Although the most recent calibration curve, IntCal20, has a resolution of 1 calendar year that does not mean 1-year resolution is significant. The measurement precision for the ^{14}C dates is, at best, 15 ^{14}C years, and often a few decades. Moreover, OxCal can be calculated for 1 year, but the default resolution of OxCal is 5 years without any interpolation. However, if the resolution is set to less than 5 years, the curve will be interpolated by a cubic function. A cubic function is a polynomial function of degree 3, which, in the case of OxCal, performs interpolation of two different data points to obtain intermediate points. This is a mathematical formulation and not a calibration of 1-year resolution. Hence, we do not take a 1-year interpolated resolution but present the raw 5-year resolution data from OxCal. For more details, we refer to https://c14.arch.ox.ac.uk/oxcalhelp/hlp_analysis_inform.html.

Furthermore, for the time range relevant for the scrolls our calibrated results are often bimodal, especially for 2σ distributions which we use for our further analyses for firmer grounding of our date-prediction model. The calibrated results from the 1990s were also often bimodal [1–3]. This bimodality is an effect of the calibration curve not being monotonous, showing peaks and other irregularities caused by variations in the cosmic ray flux which produces ^{14}C in the earth's atmosphere [20].

Table S2 shows the valid and acceptable radiocarbon date of the sample received for 4Q185 but the date cannot be used (see appendix S2.5).

Samples of the 3 scrolls 4Q216, 11Q20 and Mur88 did not produce valid ^{14}C dates; these are summarized in Table S3 (see appendix S2.6).

Table S1. Summarized results of 26 valid ^{14}C dates: laboratory code, sample identification, ^{14}C age (BP), sigma (BP), calibrated ranges (1σ and 2σ ranges) in 5-year resolution.

lab code	scroll	age BP	σ	calibrated ranges (1σ)	calibrated ranges (2σ)
GrA-68446<br>GrA-68447	P421-Fr004<br>4Q504<br>(4QDibHam[a])	2164	16	345–320,<br>205–170 BCE	355–285,<br>230–150 BCE
GrA-69793<br>GrM-10677<br>GrM-10678	P206-Fr003<br>4Q52 (4QSam[b])	2303	26	405–365 BCE	410–355,<br>285–230 BCE
GrA-69794<br>GrM-10679<br>GrM-10680	P285-Fr002<br>4Q176 (4QTanh)	2153	19	345–320,<br>205–165 BCE	355–300, 210–100,<br>70–60 BCE
GrA-69795<br>GrM-13252<br>GrM-13253<br>GrM-13254<br>GrM-13255	P224-Fr001<br>4Q114 (4QDan[c])	2168	15	345–315,<br>205–175 BCE	355–285,<br>230–160 BCE
GrM-10659<br>GrM-10660	P891-Fr003<br>5/6Hev1b (Ps)	1940	28	25–45,<br>55–125 CE	10–205 CE

Table S1 continued from previous page

lab code	scroll	age BP	σ	calibrated ranges (1σ)	calibrated ranges (2σ)
GrA-69810<br>GrM-10661<br>GrM-10662	P585-Fr001<br>4Q161 (4QpIsa^{a})	2028	18	45 BCE–10 CE	90–80 BCE,<br>55 BCE–30 CE,<br>45–60 CE
GrM-11151<br>GrM-11152<br>GrM-11170<br>GrM-11171	P1111-Fr010<br>4Q70 (4QJer^{a})[1]	2226	17	365–350,<br>295–205 BCE	375–345,<br>320–200 BCE
GrM-11153<br>GrM-11154<br>GrM-11172	P1093-Fr005<br>4Q47 (4QJosh^{a})	2155	19	345–320,<br>200–165 BCE	355–290,<br>210–100 BCE
GrM-11155<br>GrM-11156	P271-Fr002<br>4Q23 (4QLevNum^{a})	2152	24	350–315,<br>205–150,<br>130–120 BCE	355–285, 230–220,<br>210–95, 75–55 BCE
GrM-11166<br>GrM-11167<br>GrM-11184<br>GrM-11185	P177-Fr001<br>4Q255/4Q433a<br>(4QpapS^{a}/4Qpap<br>Hodayot-like Text B)	2100	17	155–90,<br>75–55 BCE	170–50 BCE
GrM-11168<br>GrM-11169<br>GrM-11186<br>GrM-11187	P977-Fr004<br>11Q5 (11QPs^{a})	1967	18	20–80,<br>100–110 CE	35–15 BCE,<br>5–120 CE
GrM-14380<br>GrM-14381<br>GrM-14228<br>GrM-14229	P393-Fr005<br>4Q3 (4QGen^{c})	2123	21	175–100,<br>70–60 BCE	340–325,<br>200–50 BCE
GrM-13385<br>GrM-13386	P1081a-Fr002<br>4Q27 (4QNum^{b})	2115	26	175–95,<br>75–55 BCE	340–330,<br>200–50 BCE
GrM-13387<br>GrM-13388<br>GrM-14175<br>GrM-14223	Px232-Fr001<br>Mas1k (MasShirShabb)	2007	18	45 BCE–25 CE	50 BCE–65 CE
GrM-14382<br>GrM-14383<br>GrM-14230<br>GrM-14241	P386-Fr001<br>4Q206 (4QEn^{e} ar)	2169	21	350–310,<br>210–170 BCE	360–280, 235–145,<br>135–120 BCE
GrM-14565<br>GrM-14566<br>GrM-14395<br>GrM-14242<br>GrM-14243	P237-Fr007<br>4Q30 (4QDeut^{c})	2182	18	355–290<br>210–175 BCE	360–275, 260–245,<br>235–165 BCE
GrM-13389<br>GrM-13390<br>GrM-14173<br>GrM-14174	P904-Fr009<br>4Q201/4Q338<br>(4QEn^{a} ar/<br>4QGenealogical List)	2077	18	110–45 BCE	165–40, 10–1 BCE
GrM-14396<br>GrM-14397<br>GrM-14244<br>GrM-14245	P810-Fr011<br>4Q259 (4QS^{e})	2148	19	345–320,<br>205–150 BCE	350–310, 210–100,<br>70–55 BCE
GrM-14398<br>GrM-14399<br>GrM-14246<br>GrM-14359	P180-Fr004<br>4Q416<br>(4QInstruction^{b})	2130	22	200–100 BCE	345–320, 205–90,<br>80–50 BCE
GrM-14400<br>GrM-14401<br>GrM-14360<br>GrM-14361	P215-Fr004<br>4Q2 (4QGen^{b})	2059	20	100–70,<br>60–35,<br>15 BCE–5 CE	155–130 BCE,<br>125 BCE–10 CE
GrM-14567	P122A-Fr001				
		2126	23	195–185,<br>180–100,<br>70–60 BCE	345–320, 205–50 BCE

Table S1 continued from previous page

lab code	scroll	age BP	σ	calibrated ranges (1σ)	calibrated ranges (2σ)
GrM-14568<br>GrM-14362<br>GrM-14363	4Q375<br>(4QapocrMoses[a])				
GrM-13391<br>GrM-13392<br>GrM-14224<br>GrM-14225	P534-Fr002<br>XHev/Se2<br>(XHev/Se Num[a])	1998	20	40–10 BCE,<br>1–30,<br>40–60 CE	45 BCE–75 CE
GrM-14569<br>GrM-14570<br>GrM-14364<br>GrM-14365	P147-Fr019<br>4Q541<br>(4QapocrLevi[b])	2148	22	345–320,<br>205–150 BCE	355–300, 210–95,<br>75–55 BCE
GrM-14571<br>GrM-14572<br>GrM-14377<br>GrM-14366	P330-Fr004<br>4Q521<br>(4QMessianic<br>Apocalypse)	2159	22	350–315,<br>205–165 BCE	355–285, 230–100 BCE
GrM-13393<br>GrM-13394<br>GrM-14226<br>GrM-14227	P107-Fr010<br>4Q267<br>(4QDamascus[b])	2151	21	345–315,<br>205–155 BCE	355–290, 210–95,<br>70–55 BCE
GrM-14573<br>GrM-14574<br>GrM-14378<br>GrM-14379	P879-Fr001<br>Mur19 pap WrDiv	1987	21	35–15 BCE,<br>5–65 CE	45 BCE–85 CE,<br>95–110 CE

As was done in the 1990s [2, 3], we also tested our procedure by dating a date-bearing manuscript, Mur19. The text of Mur19 refers to "year 6 of Masada", which is now understood as a reference from the first Jewish revolt against Rome to 71/72 CE [22–28]. The 2σ calibrated range is 45 BCE–85 CE (91.5%), 95–110 CE (3.9%). The ^{14}C date is clearly consistent with the historical date, 71/72 CE.

S2.5 Result not to be used for palaeography: 4Q185

From a radiocarbon point of view, the dating of the sample is a valid and acceptable result. However, because the sample fragment cannot be attributed to a larger manuscript, the date cannot be used for our palaeographic analysis.

For 4Q185 (4QSapiential Work), we had requested Plate 801 fragment 1. Because Plate 801 fragment 1 was sewn and encapsulated for exhibition, the IAA sent sample Plate 801 fragment 3 instead. Unfortunately, it is very uncertain that this sampled fragment is part of manuscript 4Q185. From a palaeographic perspective, identification with 4Q185 is doubtful. E.g., the letter *ayin* is different from other occurrences in the manuscript (see also [29]). For that reason, the measurement results cannot be used for our palaeographic purposes.

S2.6 Technically rejected results: 4Q216, 11Q20, and Mur88

The various AMS runs for scrolls 4Q216, Mur88, and 11Q20 resulted in internally inconsistent results. No valid ^{14}C date could be deduced. Therefore, the results are rejected for technical reasons.

For all three scrolls, different samples were received from the IAA in subsequent batches. The first samples were measured by the GrA machine, the subsequent samples were later during the project measured by the GrM machine.

[1] This fragment was previously unidentified, but see now for a positive identification [21].

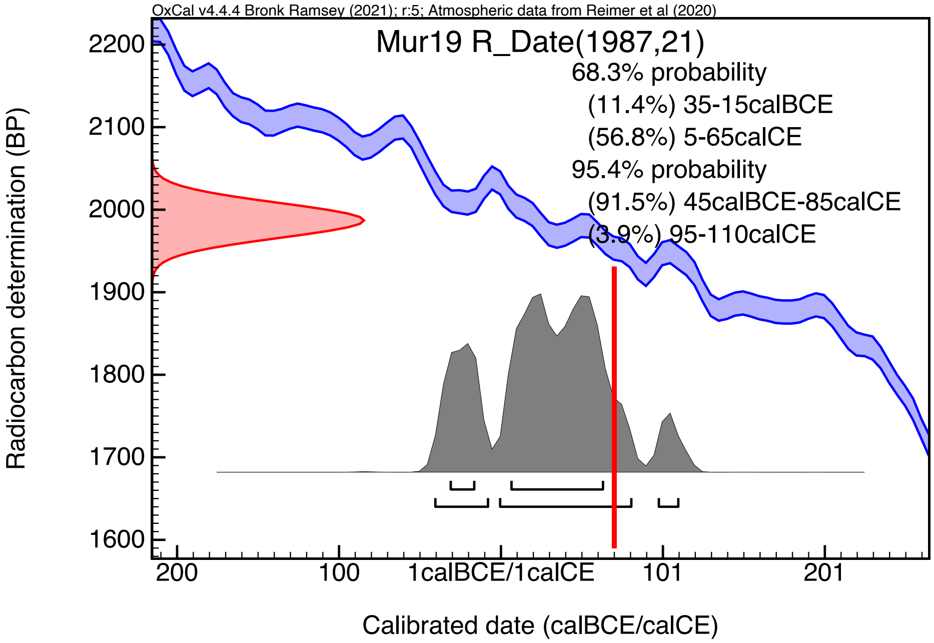

Fig S2. OxCal plot for Mur19 with **red** vertical line indicating the calendar date 71/72 CE.

Table S2. Result not to be used for palaeography: 4Q185, laboratory code, sample identification, 14C age (BP), sigma (BP), calibrated dates (1σ and 2σ ranges)

lab code	scroll	age (BP)	σ	calibrated date (1σ)	calibrated date (2σ)
GrA-68448<br>GrA-68449	P801-Fr003<br>4Q185<br>(4QSapiential Work)	2078	17	107–46 BCE	159–42, 7–5 BCE

For 4Q216 (4QJub[a]), the first sample was measured for graphite (GrA-69799). For the second sample, two gas samples were measured (GrM-10675, 10676). The GrA and GrM measurements do not provide mutually consistent dates. In other words, both samples received from the IAA do not give consistent results. In addition, the measurements yield ^{14}C dates which are impossibly old. We conclude that the sample material may not be homogeneous.

For 11Q20 (11QTemple[b]), the first sample was measured for graphite in triplicate (GrA-69800). For the second sample, two different parts of the scroll sample were taken, and two gas samples measured for each (GrM-10681, 10682, 18827, 18828). The 3 GrA measurements are internally consistent, the same for the 4 GrM results. However, GrA and GrM do not provide mutually consistent dates. Also here, both samples received from the IAA do not give consistent results. We conclude that the sample material may not be homogeneous.

For Mur88 (MurXII), the first sample was measured for graphite in triplicate (GrA-69806). For the second sample, two different parts of the scroll sample were taken, and two gas samples measured for each of them (GrM-10663, 10664, 18829, 18830). The resulting GrA and GrM measurements yield three different ^{14}C dates. Also here, the sample material may not be homogeneous.

For the full results of these runs with more details (in particular Carbon yield and δ^{13}C value) see appendix S11.

Table S3. Technically rejected results: 4Q216, 11Q20, and Mur88, laboratory code, sample identification, 14C age (BP), sigma (BP)

lab code	scroll	age (BP)	σ
GrA-69799	P385–Fr011<br>4Q216 (4QJub[a])	2342	51
GrM-10675<br>GrM-10676	P385–Fr011<br>4Q216 (4QJub[a])	2979	32
GrA-69800	P577-Fr014<br>11Q20 (11QTemple[b])	2027	24
GrM-10681<br>GrM-10682	P577-Fr014<br>11Q20 (11QTemple[b])	2183	32
GrM-18827<br>GrM-18828	P577-Fr014<br>11Q20 (11QTemple[b])	2202	26
GrA-69806	P64-Fr001<br>Mur88 (MurXII)	1950	18
GrM-10663<br>GrM-10664	P64-Fr001<br>Mur88 (MurXII)	1951	30
GrM-18829<br>GrM-18830	P64-Fr001<br>Mur88 (MurXII)	2053	25

S2.7 Analytical Chemistry

S2.7.1 Soxhlet extraction

Upon arrival of the samples in Odense, they were photographed, if this was not already done in Groningen. Detailing what was said in appendix S2.2, the chemical cleaning procedure developed to remove later added contamination such as, e.g., castor oil, was the following. Three Soxhlet apparatuses were operated in parallel, with three samples mounted simultaneously one in each chamber. The Soxhlet apparatuses had different volumes: the first one operated with 100 mL of solvent, the second with 70 mL and the third with 50 mL of solvent. All solvents were of the highest quality available (LC-grade for Liquid Chromatography).

The cleaning procedure was initiated by running the whole set of solvents with no sample mounted, intended to clean the apparatus, the stainless-steel cage and glass utensils. Then a sample was placed in the stainless-steel cage mounted in a Soxhlet apparatus chamber. The first solvent was added to the lower flask. The first solvent was LC-grade ethanol LiChrosolv (1.11727.2500 from Merck). This was operated for one hour corresponding to ca. 50 turnovers of the solvent over the sample. The second solvent was LC-grade *n*-hexane LiChrosolv (1.03701.2500 from Merck), which was operated for four hours, corresponding to ca. 240 turnovers of the solvent over the sample. The third solvent applied was LC-grade ethanol LiChrosolv (1.11727.2500 from Merck), operated for one hour, corresponding to ca. 50 turnovers. After each step in the cleaning procedure samples of 8 mL of each of the solvent were transferred to pre-cleaned glass vials. That is, three samples of 8 mL of ethanol, hexane, and ethanol were procured after each step in the cleaning procedure. They were placed in a heating apparatus operating at 80°C, which evaporated the solvents in the glass vials to dryness, after which the glass vials were sealed with a lid. The condensate was later to be re-dissolved and analyzed by HPLC-MS in Pisa (see appendix S2.7.2). After cleaning, the samples were removed from the stainless-steel cages and brought to dryness for one night at 60°C at zero humidity in a Memmert HCP 108 Climate chamber. Following this, the samples were weighed, packed, and shipped to Groningen, there to undergo pretreatment and dating following ^{14}C protocols.

This three-step Soxhlet protocol, which was developed by [12], was applied to the first batch of 10 samples (4Q52, 4Q114, 4Q161, 4Q176, 4Q185, 4Q216, 4Q504, 11Q20, Mur88, 5/6Hev1b) which were analyzed in the project. Following the chromatographic-mass spectrometric analyses in Pisa of this first set of solvents, it was decided that a fourth cleaning step should be added to the procedure for

the remaining 20 samples. This was done for redundancy, and not because of proof or suspicion that the three-step procedure was not sufficient within the given dating uncertainty. The fourth step was added to further ensure that castor oil and many other contaminants were removed even in the worst case scenario. The fourth Soxhlet step was performed using a 30:70 mixture of dichloromethane:hexane, both of LC-grade purity (dicholoromethane CHROMASOLV 34856 by Sigma-Aldrich, and *n*-hexane as described above), operated for one hour, corresponding to ca. 60 turnovers of the solvent over the sample.

S2.7.2 Raman spectroscopy, optical microscopy, Py-GC/MS, and HPLC-MS analysis

The study of the materials constituting the scrolls was performed in Pisa using a multi-analytical approach based on chromatographic and spectroscopic analytical techniques. The use of these complementary approaches allowed us to characterize both the original materials of the parchments and to evaluate the possible occurrence of modern materials used for consolidating/restoring the scrolls. These results were used to define the best cleaning strategy to remove from the scrolls the modern materials that could affect the dating, and to evaluate the efficiency of the purification steps. In detail:

- *Raman spectroscopy and optical microscopy* (OM) were used as non-invasive and non-destructive methods to evaluate the general appearance of the parchments and to characterize the possible occurrence of inorganic materials.
- *Analytical pyrolysis coupled with gas chromatography and mass spectrometry* (Py-GC/MS) analyses were performed on small (ca. 100 µg) sub-samples of the samples before these went into cleaning treatment by Soxhlet and AAA to characterize the organic material constituting the scrolls and to evaluate the possible presence of modern synthetic materials used as consolidating materials. This technique represents one of the best methods to obtain a complete picture of the organic materials in a sample [30]. Pyrolysis consists of a thermal decomposition of organic materials in absence of oxygen. This process leads to the formation of low molecular weight species that can be separated by gas chromatography and identified by mass spectrometry. This analytical approach allows to obtain specific molecular markers that can be used to identify the source of organic materials.
- *Liquid chromatography coupled with mass spectrometry* (HPLC-MS) was applied to evaluate the content of lipid materials present in Soxhlet extracts from parchments during the cleaning steps. This is among the best approaches for the separation and characterization of complex mixtures of lipid materials, such as castor oil. The use of mass spectrometry as detection system allows to obtain information on the glyceride chemical structure [31]. This information cannot be achieved using more conventional analytical approaches such as GC/MS. Moreover, this method allows to detect very low amounts of analytes.

S2.7.3 Results of the optical microscopy and Raman spectroscopy analyses performed on 17 samples

The microscopy observations and micro-Raman analyses were performed on samples 4Q2, 4Q3, 4Q27, 4Q30, 4Q114, 4Q201/4Q338, 4Q206, 4Q216, 4Q259, 4Q267, 4Q375, 4Q416, 4Q521, 4Q541, Mas1k, Mur19, XHev/Se2. All these samples were characterized by similar appearance, except for sample Mur19 that showed a different morphology, suggesting the use of a different material as a writing support.

Several samples featured microscopic black spots with diameters in the range of 10-200 µm, except for sample 4Q114 that was characterized by one black spot of approximately 600 µm. Raman spectroscopy was applied to investigate the chemical composition of the spots. For several samples, the Raman spectra featured the typical peaks at 1350 and 1580 cm^{-1} corresponding to the Raman wavenumbers typical of C-C of amorphous carbon (signals not detected in the background). For example, Fig S3 reports the spectrum obtained from one spot on the sample 4Q216, and Table S4 presents the OM photographs along with a description of the observed surface and summarizes the relevant information obtained by Raman spectroscopy.

The biggest black spot from the scroll 4Q114 was sampled separately, and radiocarbon dated to 2390±60 BP (GrM-13256). The size of the black spot was ca. 600 µm in diameter, with an observed thickness of ca. 50 µm, translating into a calculated mass of ca. 14 µg. Thus, with a sample mass of 6.2 mg for the sample radiocarbon dated for 4Q114, the contamination mass fraction from the black spot would be ca. 0.2% and thus, the effect of such contamination is negligible, whatever its age.

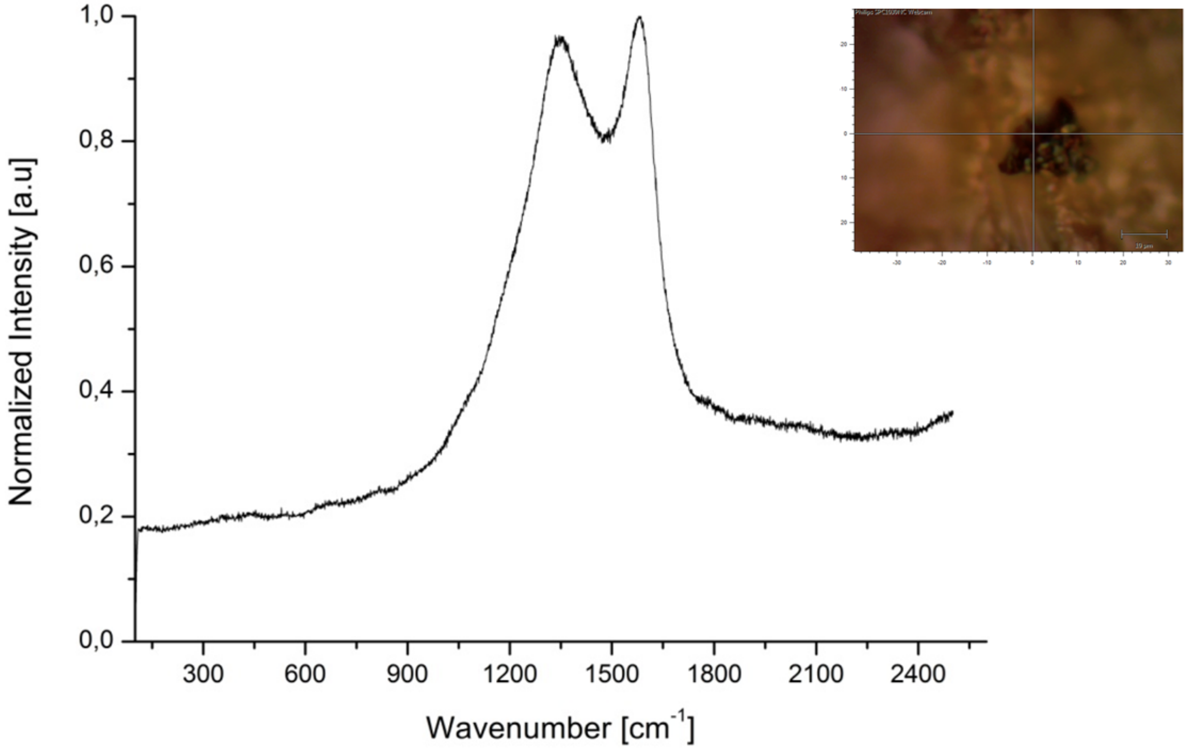

Fig S3. Raman spectrum obtained for one spot in sample 4Q216

Table S4. Optical microscope pictures and observations

Sample	MO side A	MO side B	Observation
**4Q3**	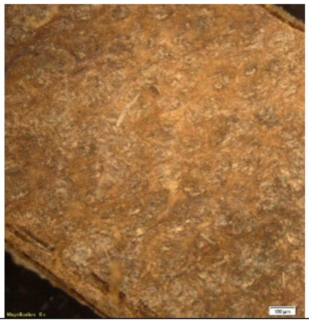	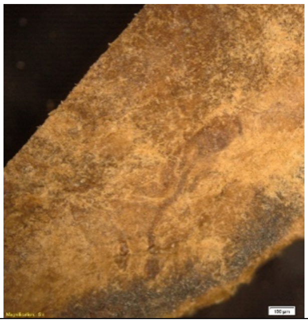	Only one detectable black spot. Carbon was identified by Raman analysis.

Table S4 continued from previous page

**4Q27**	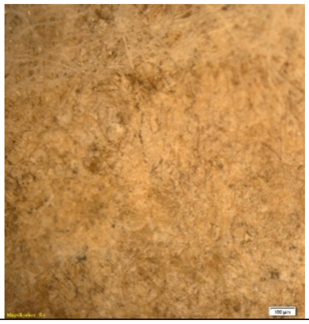	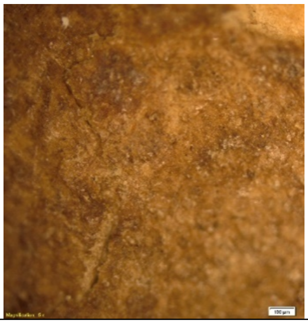	Only one detectable black spot. Carbon was identified by Raman analysis.
**Mas1k**	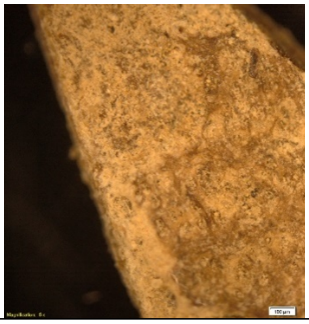	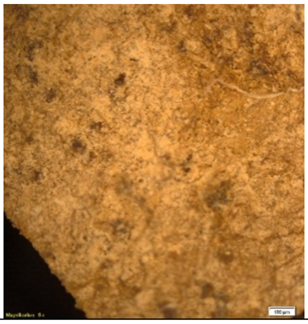	The surface of the sample was characterized by high fluorescence and few dark spots. One spot was identified as carbon by Raman analysis.
**4Q206**	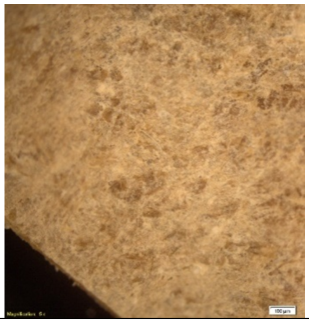	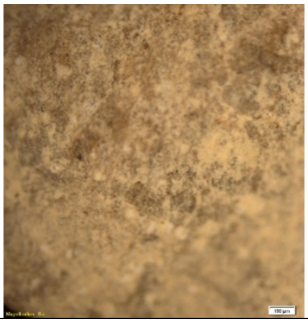	The surface was characterized by the presence of few black spots and few red spots: Raman analysis revealed the presence of carbon and hematite.
**4Q30**	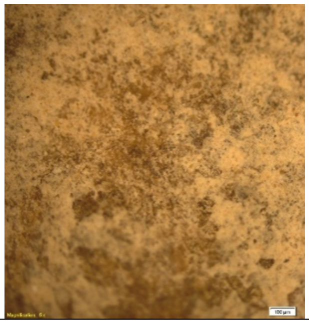	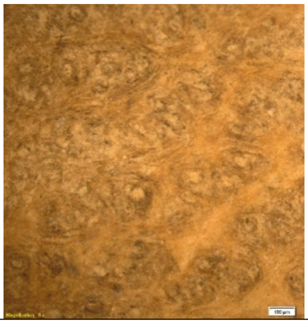	The sample was characterized by the presence of a few black spots with a size larger than 10 µm. Carbon was identified.

Table S4 continued from previous page

**4Q201/ 4Q338**	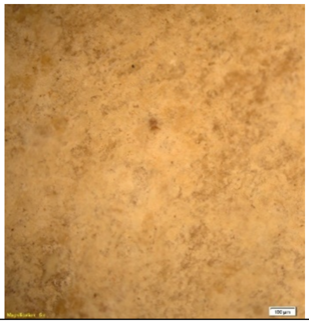	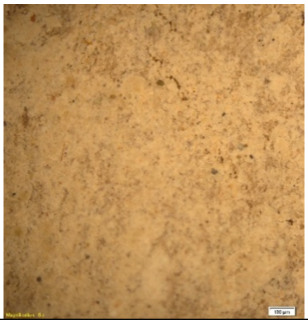	The sample was characterized by several black spots. Several red spots were also detected for sample 386. Due to the high fluorescence of the writing support, Raman spectra evidenced only the presence of carbon.
**4Q259**	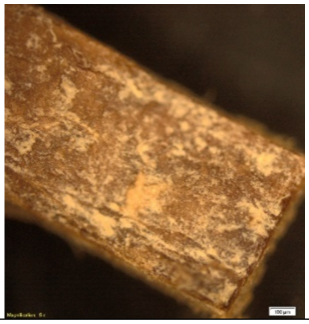	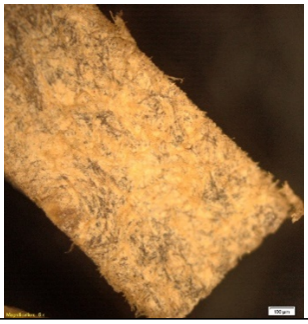	Almost clean, no significant spots were detected.
**4Q416**	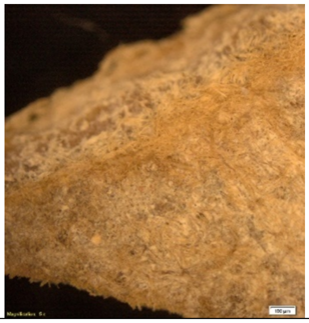	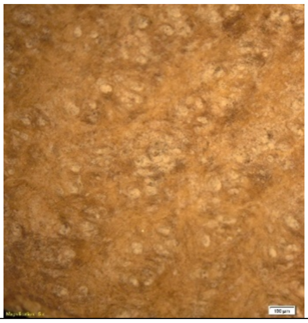	The sample was characterized by a rough surface scattering the laser Raman light. The MO observation did not show any significant presence of dark spots.
**4Q2**	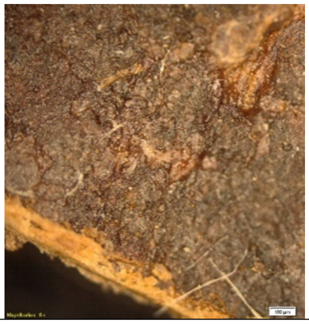	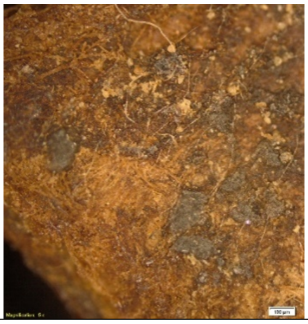	The sample was fully covered by a material that did not allow to perform a proper Raman analysis.

Table S4 continued from previous page

**4Q375**	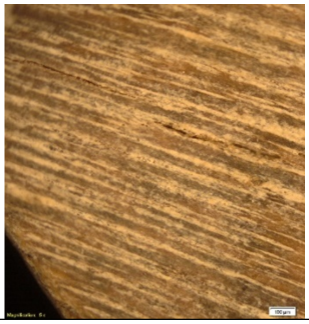	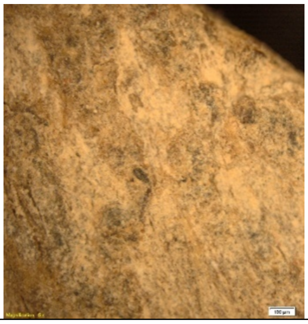	The sample was characterized by a few black spots with a diameter wider than 20 µm. Carbon was identified.
**XHev/ Se2**	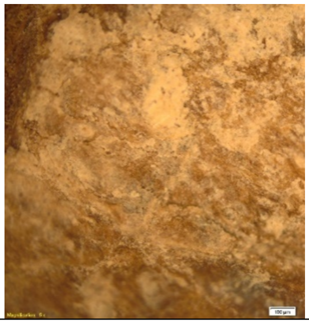	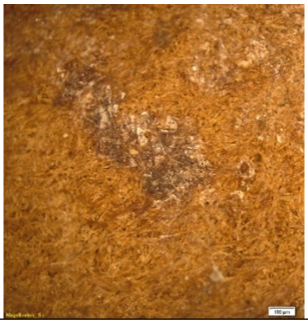	The sample was almost clean. Only two 10 µm in diameter black spots were detected. Carbon was identified.
**4Q541**	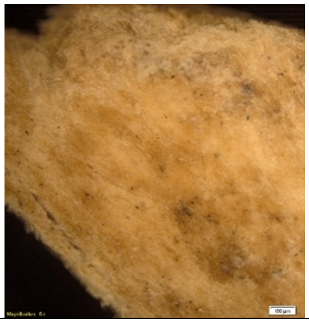	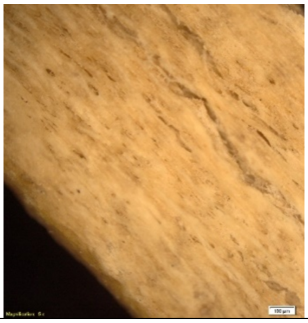	The sample was characterized by several black spots. Carbon was identified.
**4Q521**	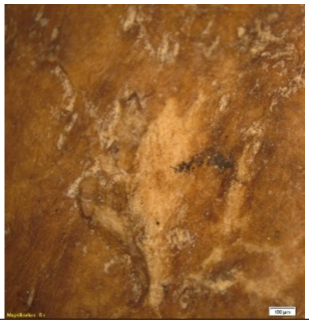	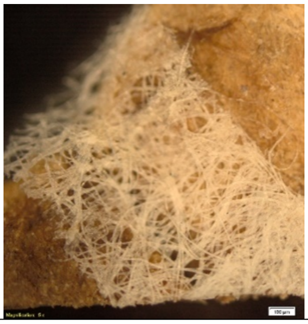	Only one big black spot (100-150 µm) was detected on the surface of the bigger fragment. Carbon was identified.

Table S4 continued from previous page

**4Q267**	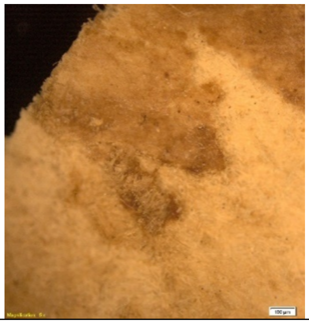	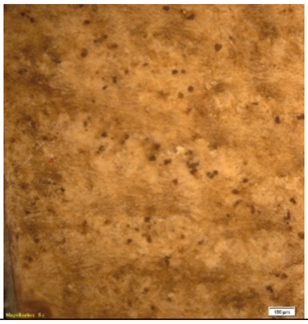	The sample was almost clean from dark spots. The size of the identified spots was too small to be investigated by Raman.
**Mur19**	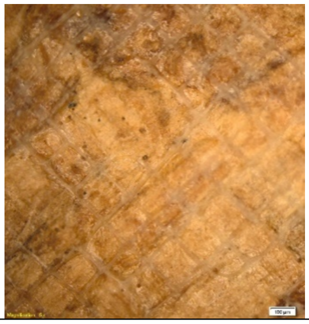	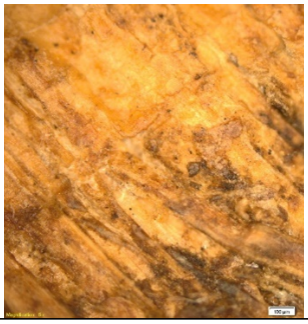	The sample was characterized by several black spots and an organic protective. Carbon was identified.
**4Q216**	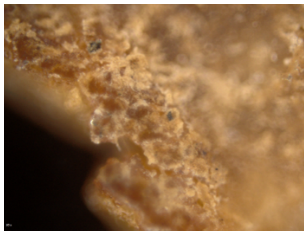	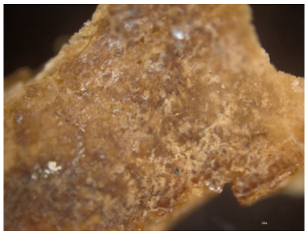	The sample was characterized by black spots. Carbon was identified.
**4Q114**	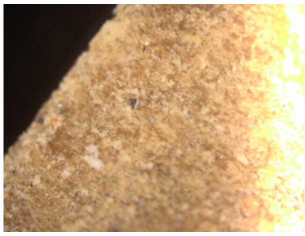	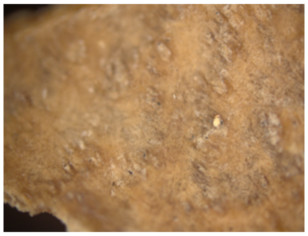	The sample was characterized by black spots. Carbon was identified.

S2.7.4 Results of the Py-GC/MS analysis performed on 17 samples

Py-GC/MS was used in order to evaluate the possible presence of synthetic materials used as consolidating materials on the scrolls and to characterize the original parchment material: the 17 samples (4Q2, 4Q3, 4Q27, 4Q30, 4Q114, 4Q201/4Q338, 4Q206, 4Q216, 4Q259, 4Q267, 4Q375, 4Q416, 4Q521, 4Q541, Mas1k, Mur19, XHev/Se2) were directly analyzed without any prior sample pretreatment using a multi-shot pyrolyzer EGA/PY-3030D (Frontier Lab, Japan) coupled with a 6890 N gas chromatography system with a split/splitless injection port, and with a 5973 mass selective single quadrupole mass spectrometer (Agilent Technologies). The complete instrumental conditions are reported in [32].

The pyrolytic profile of all these 17 samples featured molecular markers that can be related to the pyrolysis of animal hide or scroll (pyrrole and diketopiperazines), except for sample Mur19 that was instead characterized by the presence of anhydro sugars and levoglucosan, typical of a cellulose-based material [33]. This is consistent with the observation that Mur19 is a papyrus fragment. Fig S4 reports the chromatogram obtained for the sample from the parchment of 4Q521.

Samples 4Q3 and 4Q206 showed the presence of the markers of polyethylene glycol. The pyrograms of samples 4Q3 and 4Q30 also contain the peaks due to hexadecanonitrile and octadecanonitrile, which are the Py-GC/MS markers characteristic for egg. Samples 4Q521 and Mur19 were characterized by the presence of an acrylic resin. Finally, samples 4Q2, 4Q267, 4Q541, and Mur19 showed the presence of retene: this molecule is a marker characteristic of the combustion of resinous wood and can be indicative of the exposure of the scrolls to a fire in the space where writing took place or could be due to residues related to the illumination with torches. Table S5 summarizes the materials detected in the different parchment samples.

Pyrolysis allowed us to pinpoint the presence of exogenous materials, as consolidation synthetic materials (acrylic resin), or lipids. After disclosing the nature of the contamination, we were able to design the proper cleaning procedures to remove any unwanted consolidant.

The use of a further cleaning step using dichloromethane ensured the total removal of all the synthetic materials, as proven by pyrolysis analyses performed on a subsection of the samples after cleaning and prior to ^{14}C dating.

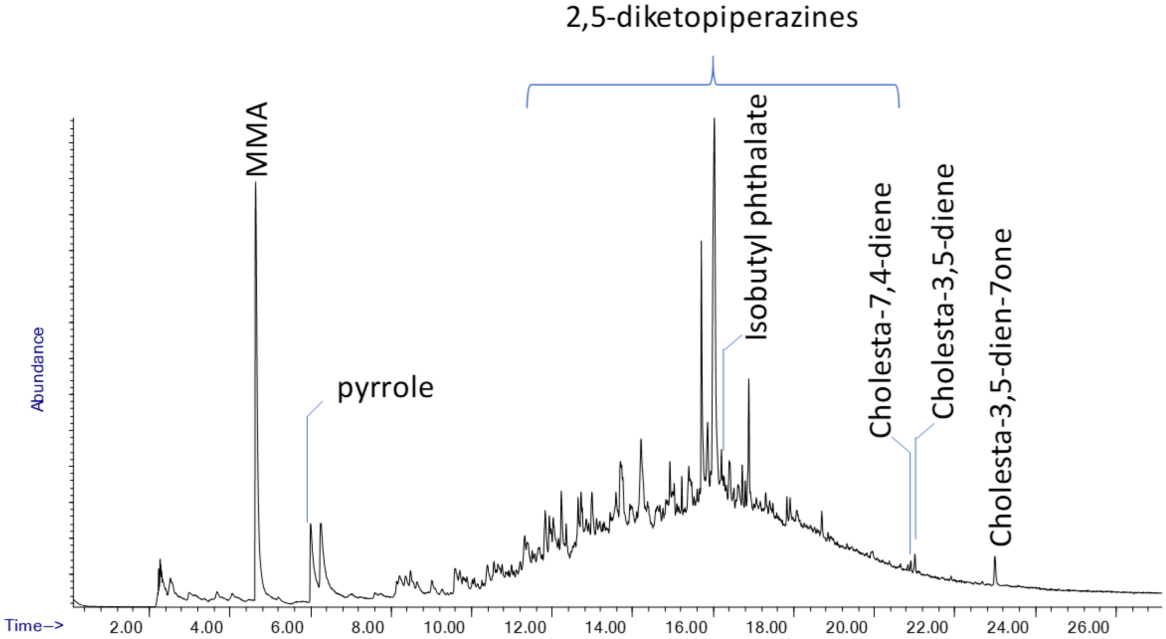

Fig S4. Py-GC-MS chromatogram of sample 4Q521: parchment sample with methyl-methacrylate, an acrylic resin.

S2.7.5 Liquid chromatography-mass spectrometry results of the analysis of residual lipids in the extracts from the 30 samples after cleaning

HPLC-MS was applied to evaluate the presence of lipid materials. The dried extracts were reconstituted in 150 µL of iso-propanol/methanol, 10:90, filtered (PTFE syringe, 0.45 µm pore size) and analyzed. HPLC-ESI-Q-ToF analyses were carried out using a 1200 Infinity HPLC, coupled with a Quadrupole-Time of Flight tandem mass spectrometer 6530 Infinity Q-ToF detector by a Jet Stream ESI interface (Agilent Technologies, USA). The complete instrumental conditions are reported in [34].

Table S5. Summary of the materials detected in the different parchment samples.

Samples	Identified organic materials
4Q216	proteinaceous material, lipid material
4Q3	proteinaceous material, lipid material, egg
4Q27	proteinaceous material, lipid material
Mas1k	proteinaceous material, lipid material
4Q206	proteinaceous material, lipid material
4Q30	proteinaceous material, lipid material, egg
4Q201/4Q338	proteinaceous material, lipid material
4Q259	proteinaceous material, lipid material
4Q416	proteinaceous material, lipid material
4Q2	proteinaceous material, lipid material, retene
4Q375	proteinaceous material, lipid material
XHev/Se2	proteinaceous material, lipid material
4Q541	proteinaceous material, lipid material, retene
4Q521	proteinaceous material, lipid material, acrylic resin
4Q267	proteinaceous material, lipid material, retene
Mur19	lignocellulose material, acrylic resin, retene
4Q114	proteinaceous material, lipid material

The analyses were performed on the extracts from the two different sample pretreatments by Soxhlet, i.e., the three-step and the four-step extraction.

The comparison of the results obtained on the extracts with reference blanks allowed us to highlight the effective performances of the cleaning procedures, showing that the glyceride content after the last step was below 7.0 micrograms for both the approaches. The cleaning procedure proved to be effective for removing the lipid materials from the scroll samples, since all the solutions obtained after the last extraction step were characterized by the presence of triglycerides and fatty acids at or below blank level. Fig S5 shows a comparison of all final cleaning steps with the respective blanks for both the fatty acids and the triacylglycerols.

In particular, the worst case encountered in the entire data set was 7.0 µg of acylglycerols detected in the fourth cleaning step of 4Q3. These triglycerides can originate from the original parchment, or they can originate from later contamination such as castor oil. There is no way to determine the origin; it can also be a mixture of ancient and recent materials. If we, as a worst-case scenario, assume that all the triglycerides detected in 4Q3 were modern contamination, then it would skew a 2000-years old parchment sample with only 12.6 years.

As stipulated, this is a worst-case scenario depending on all triglycerides to be modern, which is an unlikely assumption because triglycerides are a normal ingredient of animal skin [35]. Furthermore, all other samples are well below the 7.0 µg level.

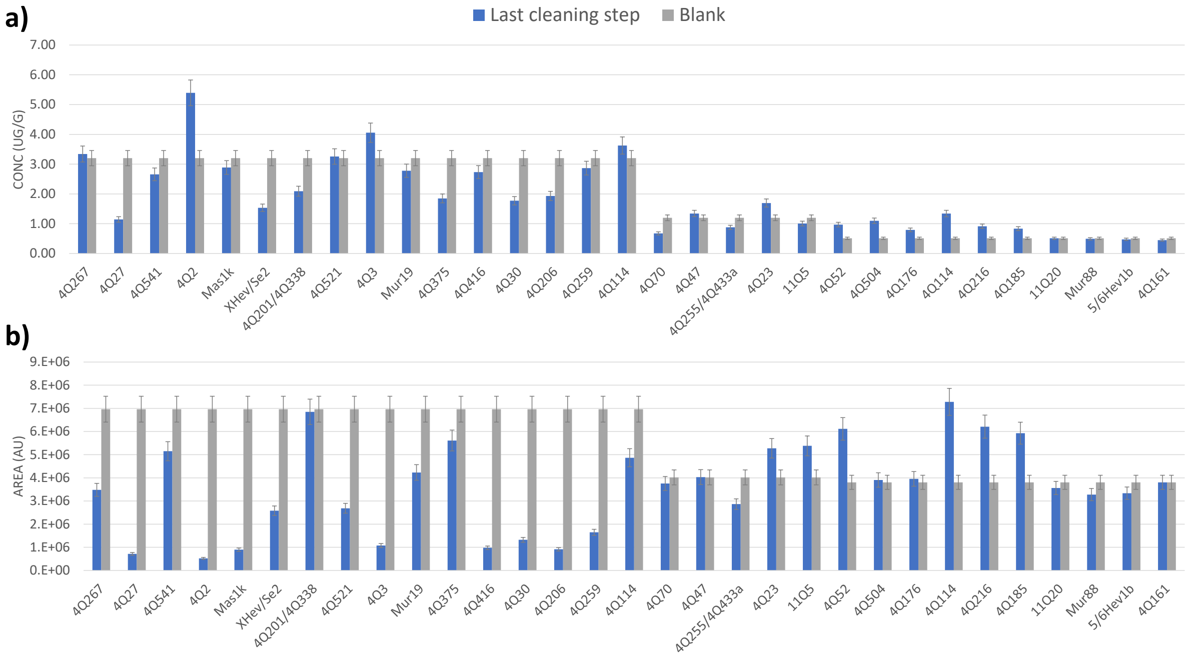

Fig S5. *Top (a)*: Comparison of the free fatty acid concentrations between the blank samples and the final cleaning steps; *Bottom (b)*: comparison of the abundances of TAGs (triacylglycerols) in the last cleaning step with those found in the blank samples (AU: arbitrary unit).

References

1. Bonani G, Broshi M, Carmi I, Ivy S, Strugnell J, Wölfli W. Radiocarbon Dating of the Dead Sea Scrolls. Atiqot. 1991;20:27–32.
2. Bonani G, Ivy S, Wölfli W, Broshi M, Carmi I, Strugnell J. Radiocarbon Dating of Fourteen Dead Sea Scrolls. Radiocarbon. 1992;34:843–849. doi:10.1017/s0033822200064158.
3. Jull AJT, Donahue DJ, Broshi M, Tov E. Radiocarbon Dating of Scrolls and Linen Fragments from the Judean Desert. Radiocarbon. 1995;37:11–19. doi:10.1017/s0033822200014740.
4. Brink A, Bulacu M, Schomaker L. How much handwritten text is needed for text-independent writer verification and identification. In: 2008 19th International Conference on Pattern Recognition (ICPR). Piscataway: IEEE; 2008.
5. Yardeni A. Textbook of Aramaic, Hebrew and nabataean documentary texts from the judaean desert and related material, 2 vols. Jerusalem: The Hebrew University; 2000.
6. Cross FM. The Development of the Jewish Scripts. In: Leaves from an Epigrapher's Notebook: Collected Papers in Hebrew and West Semitic Palaeography and Epigraphy. Winona Lake, IN: Eisenbrauns; 2003. p. 1–43.
7. Popović M. Book Production and Circulation in Ancient Judaea: Evidenced by Writing Quality and Skills in the Dead Sea Scrolls Isaiah and Serekh Manuscripts. In: Williams TB, Keith C, Stuckenbruck L, editors. The Dead Sea Scrolls in Ancient Media Culture. Leiden: Brill; 2023. p. 199–265.
8. Tigchelaar E. Seventy Years of Palaeographic Dating of the Dead Sea Scrolls. In: Drawnel H, editor. Sacred Texts and Disparate Interpretations: Qumran Manuscripts Seventy Years Later. Leiden: Brill; 2020. p. 258–278.
9. Longacre D. Disambiguating the Concept of Formality in Palaeographic Descriptions: Stylistic Classification and the Ancient Jewish Hebrew/Aramaic Scripts. Comparative Oriental Manuscript Studies Bulletin. 2019;5:101–128. doi:10.25592/UHHFDM.739.
10. Rasmussen KL, van der Plicht J, Cryer FH, Doudna G, Cross FM, Strugnell J. The effects of possible contamination on the radiocarbon dating of the Dead Sea Scrolls I: castor oil. Radiocarbon. 2001;43:127–132. doi:10.1017/S0033822200031702.
11. Rasmussen KL, van der Plicht J, Doudna G, Cross FM, Strugnell J. Reply to Israel Carmi (2002): "Are the 14C Dates of the Dead Sea Scrolls Affected by Castor Oil Contamination?". Radiocarbon. 2003;45:497–499. doi:10.1017/S0033822200032847.
12. Rasmussen KL, van der Plicht J, Doudna G, Nielsen F, Højrup P, Stenby EH, et al. The effects of possible contamination on the radiocarbon dating of the Dead Sea Scrolls II: empirical methods to remove castor oil and suggestions for redating. Radiocarbon. 2009;51:1005–1022. doi:10.1017/S0033822200034081.
13. Queffelec A, Bertran P, Bos T, Lemée L. Mineralogical and organic study of bat and chough guano: implications for guano identification in ancient context. Journal of Cave and Karst Studies. 2018;80:1–17. doi:10.4311/2017es0102.
14. Dee MW, Palstra SWL, Aerts-Bijma AT, Bleeker MO, de Bruijn S, Ghebru F, et al. Radiocarbon Dating at Groningen: New and Updated Chemical Pretreatment Procedures. Radiocarbon. 2019;62:63–74. doi:10.1017/rdc.2019.101.

15. Van der Plicht J, Wijma S, Aerts A, Pertuisot M, Meijer H. Status report: the Groningen AMS facility. Nuclear Instruments and Methods in Physics Research Section B: Beam Interactions with Materials and Atoms. 2000;172:58–65. doi:10.1016/S0168-583X(00)00284-6.

16. Synal HA, Stocker M, Suter M. MICADAS: A new compact radiocarbon AMS system. Nuclear Instruments and Methods in Physics Research Section B: Beam Interactions with Materials and Atoms. 2007;259:7–13. doi:10.1016/j.nimb.2007.01.138.

17. Mook WG, van der Plicht J. Reporting 14C activities and concentrations. Radiocarbon. 1999;41:227–239. doi:10.1017/S0033822200057106.

18. Reimer PJ, Austin WE, Bard E, Bayliss A, Blackwell PG, Ramsey CB, et al. The IntCal20 Northern Hemisphere radiocarbon age calibration curve (0–55 cal kBP). Radiocarbon. 2020;62:725–757. doi:10.1017/RDC.2020.41.

19. Ramsey CB. Development of the Radiocarbon Calibration Program. Radiocarbon. 2001;43:355–363. doi:10.1017/s0033822200038212.

20. van der Plicht J. Variations in atmospheric ^{14}C. In: Reference Module in Earth Systems and Environmental Sciences. Encyclopedia of Quaternary Science, 3rd Edition. Amsterdam: Elsevier; 2022. p. 1–10.

21. Tigchelaar E. Identification and Reidentification of Some Fragments of 4Q70 (4QJer^{a}). Textus. 2020;29:193–200. doi:10.1163/2589255x-02901006.

22. Benoit P, Milik JT, de Vaux R. Discoveries in the Judaean Desert: Volume II. Les grottes de Murabbaʿât, 2 Vols. Oxford: Clarendon Press; 1961.

23. Koffmahn E. Zur Datierung der aramäisch/Hebräischen Vertragsurkunden von Murabbaʿat. Wiener Zeitschrift für die Kunde des Morgenlandes. 1963;59:119–136.

24. Yadin Y. The excavation of Masada—1963/64: preliminary report. Israel Exploration Journal. 1965;15:1–120.

25. Goodblatt D. Dating Documents in Provincia Iudaea: A Note on Papyri Murabbaʿat 19 and 20. Israel Exploration Journal. 1999;49:249–259.

26. Eshel H. Documents of the First Jewish Revolt from the Judean Desert. In: Berlin AM, Overman JA, editors. The First Jewish Revolt: Archaeology, History, and Ideology. London: Routledge; 2003. p. 171–177.

27. Eshel H, Broshi M, Jull TAJ. Four Murabbaʿat Papyri and the Alleged Capture of Jerusalem by Bar Kokhba. In: Katzoff R, Schaps D, editors. Law in the Documents of the Judaean Desert. Leiden: Brill; 2005. p. 45–50.

28. Wise MO. Language and literacy in Roman Judaea. New Haven, CT: Yale University Press; 2015.

29. Pajunen M. 4QSapiential Admonitions B (4Q185): Unsolved Challenges Of The Hebrew Text. In: Brooke G, Høgenhaven J, editors. The Mermaid and the Partridge. Leiden: Brill; 2011. p. 191–220.

30. Degano I, Modugno F, Bonaduce I, Ribechini E, Colombini MP. Recent advances in analytical pyrolysis to investigate organic materials in heritage science. Angewandte Chemie International Edition. 2018;57:7313–7323. doi:10.1002/anie.201713404.

31. La Nasa J, Modugno F, Degano I. Liquid chromatography and mass spectrometry for the analysis of acylglycerols in art and archeology. Mass Spectrometry Reviews. 2021;40:381–407. doi:10.1002/mas.21644.

32. La Nasa J, Biale G, Sabatini F, Degano I, Colombini MP, Modugno F. Synthetic materials in art: a new comprehensive approach for the characterization of multi-material artworks by analytical pyrolysis. Heritage Science. 2019;7:1–14. doi:10.1186/s40494-019-0251-4.

33. Colombini MP, Modugno F. Organic mass spectrometry in art and archaeology. Hoboken, NJ: John Wiley & Sons; 2009.

34. La Nasa J, Ghelardi E, Degano I, Modugno F, Colombini MP. Core shell stationary phases for a novel separation of triglycerides in plant oils by high performance liquid chromatography with electrospray-quadrupole-time of flight mass spectrometer. Journal of Chromatography A. 2013;1308:114–124. doi:10.1016/j.chroma.2013.08.015.

35. Ghioni C, Hiller JC, Kennedy CJ, Aliev A, Odlyha M, Boulton M, et al. Evidence of a distinct lipid fraction in historical parchments: a potential role in degradation? Journal of lipid research. 2005;46:2726–2734. doi:10.1194/jlr.M500331-JLR200.
