# Supplementary material for: Dating ancient manuscripts using radiocarbon and AI-based writing style analysis
Source: PLoS One. 2025 Jun 4;20(6):e0323185. doi: 10.1371/journal.pone.0323185 (PMC12136314; doi:10.1371/journal.pone.0323185)
Supplement: S3 Appendix — (PDF) [file pone.0323185.s003.pdf]

S3 Appendix for the article:

Dating ancient manuscripts using radiocarbon and AI-based writing style analysis

Mladen Popović[1*], Maruf A. Dhali[1,2], Lambert Schomaker[2], Johannes van der Plicht[3], Kaare Lund Rasmussen[4], Jacopo La Nasa[5], Ilaria Degano[5], Maria Perla Colombini[5], Eibert Tigchelaar[6],

1 Qumran Institute, University of Groningen, 9712 GK, The Netherlands
2 Artificial Intelligence, Bernoulli Institute, University of Groningen, 9747 AG, The Netherlands
3 Center for Isotope Research, University of Groningen, 9747 AG, The Netherlands
4 Department of Physics, Chemistry, and Pharmacy, University of Southern Denmark, DK 5230, Denmark
5 Department of Chemistry and Industrial Chemistry, University of Pisa, 56126 Pisa PL, Italy
6 Faculty of Theology and Religious Studies, KU Leuven, 3000 Leuven, Belgium

* m.popovic@rug.nl

Data and materials: All data, code, and test film associated with this article are publicly available on Zenodo with the following DOIs:

- Data and prediction plots (v3): https://doi.org/10.5281/zenodo.10998958.
- Code and feature files (v6): https://doi.org/10.5281/zenodo.13319794.
- Film (see details in S7 Appendix: https://doi.org/10.5281/zenodo.8167946.

Please note that this article has 12 appendices in total, from **S1** to **S12**.

C

S3 OxCal plots: ^{14}C determinations and calibrated date plots

Here, we present the OxCal plots for the 26 valid samples. No plots were produced for the 3 technically rejected samples (see appendix S2.6), nor for the 1 sample of which the identity could not be ascertained (see appendix S2.5).

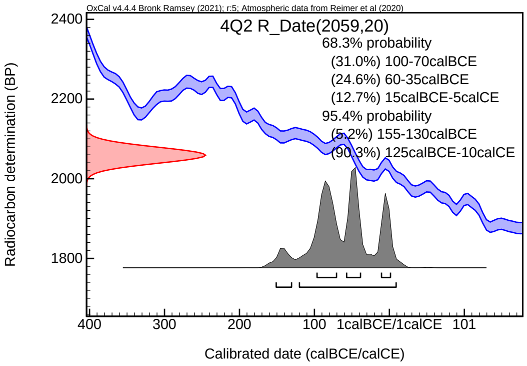

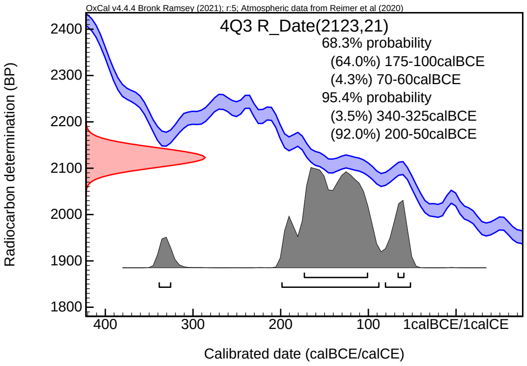

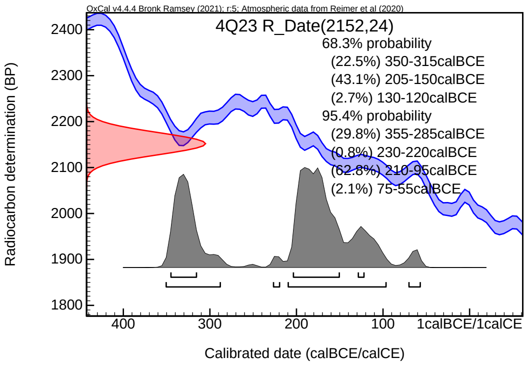

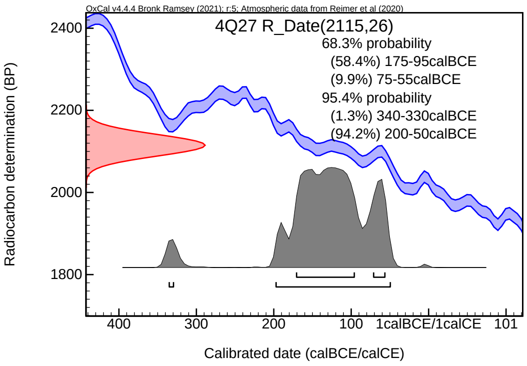

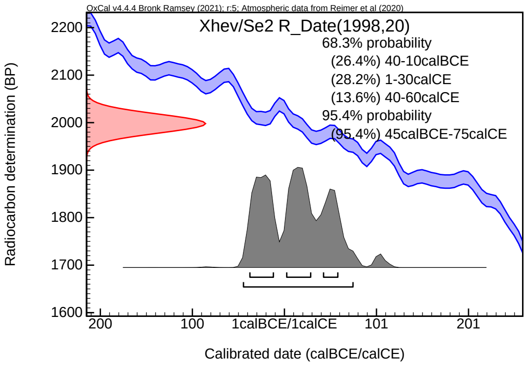

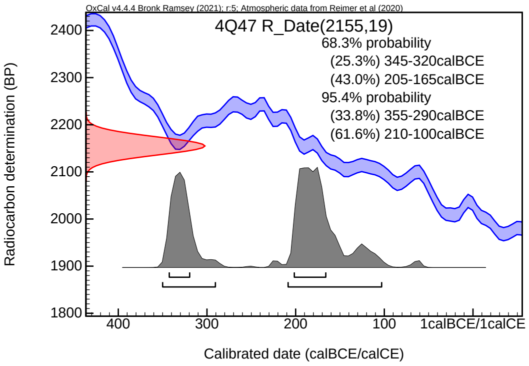

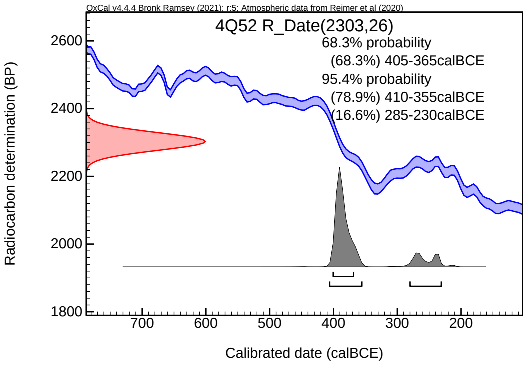
OxCal v4.4.4 Bronk Ramsey (2021); r:5; Atmospheric data from Reimer et al (2020)
4Q52 R_Date(2303,26)
68.3% probability
(68.3%) 405-365calBCE
95.4% probability
(78.9%) 410-355calBCE
(16.6%) 285-230calBCE
Radiocarbon determination (BP)
Calibrated date (calBCE)

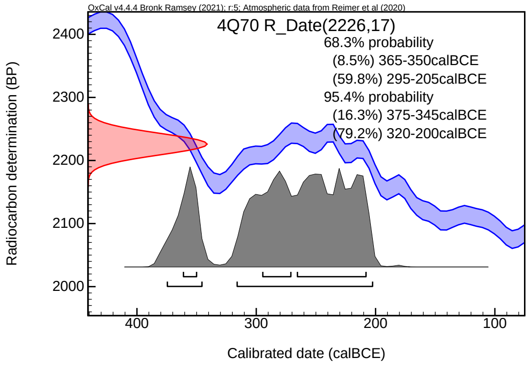
OxCal v4.4.4 Bronk Ramsey (2021); r:5; Atmospheric data from Reimer et al (2020)
4Q70 R_Date(2226,17)
68.3% probability
(8.5%) 365-350calBCE
(59.8%) 295-205calBCE
95.4% probability
(16.3%) 375-345calBCE
(79.2%) 320-200calBCE
Radiocarbon determination (BP)
Calibrated date (calBCE)

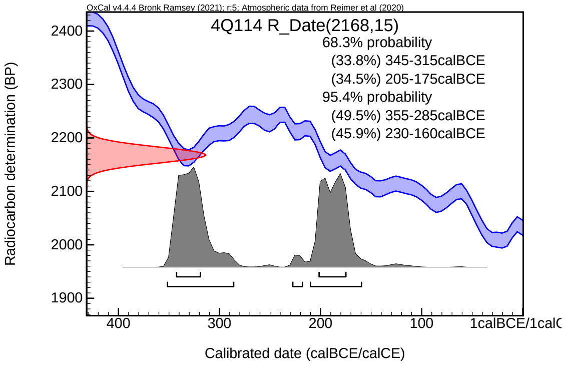
OxCal v4.4.4 Bronk Ramsey (2021); r:5; Atmospheric data from Reimer et al (2020)
4Q114 R_Date(2168,15)
68.3% probability
(33.8%) 345-315calBCE
(34.5%) 205-175calBCE
95.4% probability
(49.5%) 355-285calBCE
(45.9%) 230-160calBCE
Radiocarbon determination (BP)
Calibrated date (calBCE/calCE)

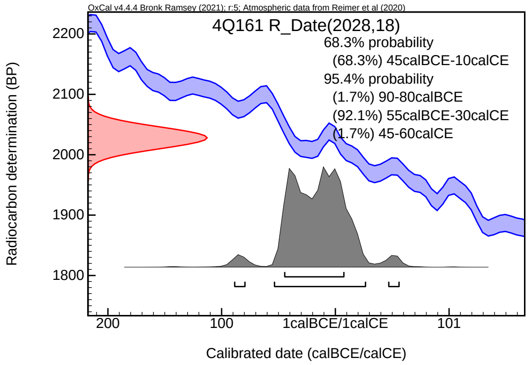
OxCal v4.4.4 Bronk Ramsey (2021); r:5; Atmospheric data from Reimer et al (2020)
4Q161 R_Date(2028,18)
68.3% probability
(68.3%) 45calBCE-10calCE
95.4% probability
(1.7%) 90-80calBCE
(92.1%) 55calBCE-30calCE
(1.7%) 45-60calCE
Radiocarbon determination (BP)
Calibrated date (calBCE/calCE)

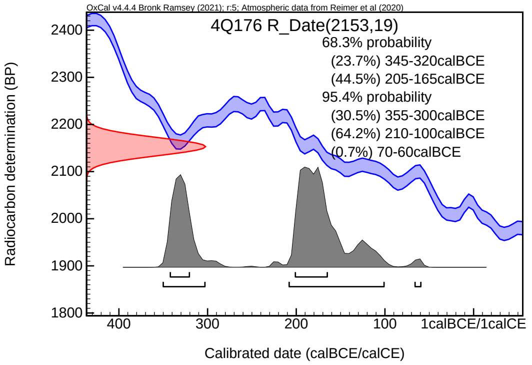
OxCal v4.4.4 Bronk Ramsey (2021); r:5; Atmospheric data from Reimer et al (2020)
4Q176 R_Date(2153,19)
68.3% probability
(23.7%) 345-320calBCE
(44.5%) 205-165calBCE
95.4% probability
(30.5%) 355-300calBCE
(64.2%) 210-100calBCE
(0.7%) 70-60calBCE
Radiocarbon determination (BP)
Calibrated date (calBCE/calCE)

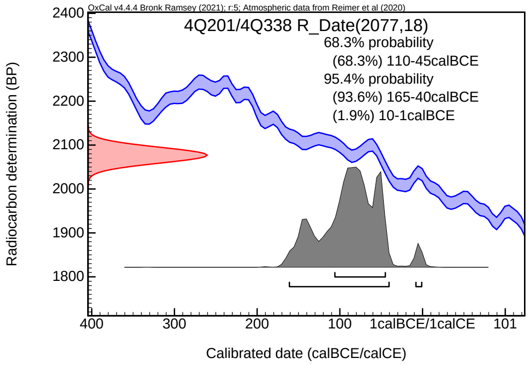
OxCal v4.4.4 Bronk Ramsey (2021); r:5; Atmospheric data from Reimer et al (2020)
4Q201/4Q338 R_Date(2077,18)
68.3% probability
(68.3%) 110-45calBCE
95.4% probability
(93.6%) 165-40calBCE
(1.9%) 10-1calBCE
Radiocarbon determination (BP)
Calibrated date (calBCE/calCE)

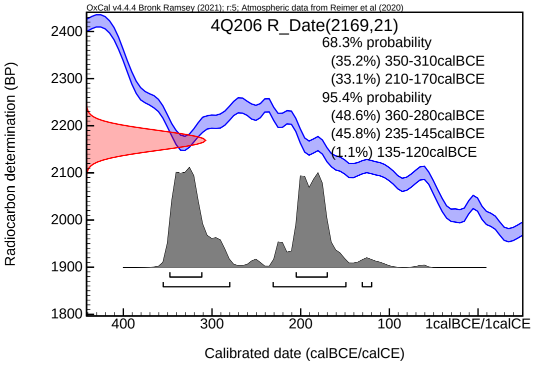
OxCal v4.4.4 Bronk Ramsey (2021); r:5; Atmospheric data from Reimer et al (2020)
4Q206 R_Date(2169,21)
68.3% probability
(35.2%) 350-310calBCE
(33.1%) 210-170calBCE
95.4% probability
(48.6%) 360-280calBCE
(45.8%) 235-145calBCE
(1.1%) 135-120calBCE
Radiocarbon determination (BP)
Calibrated date (calBCE/calCE)

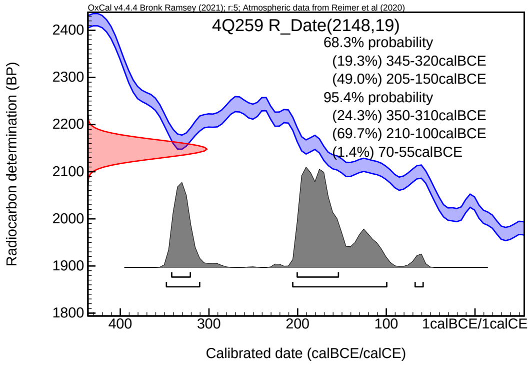
OxCal v4.4.4 Bronk Ramsey (2021); r:5; Atmospheric data from Reimer et al (2020)
4Q259 R_Date(2148,19)
68.3% probability
(19.3%) 345-320calBCE
(49.0%) 205-150calBCE
95.4% probability
(24.3%) 350-310calBCE
(69.7%) 210-100calBCE
(1.4%) 70-55calBCE
Radiocarbon determination (BP)
Calibrated date (calBCE/calCE)

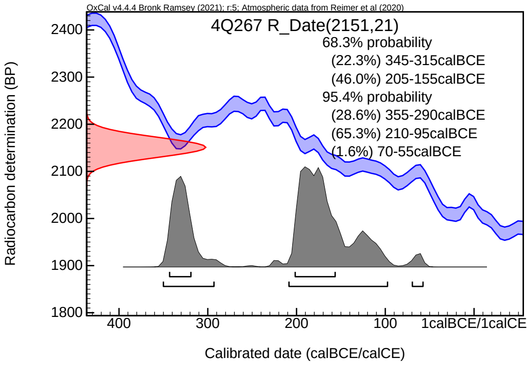
OxCal v4.4.4 Bronk Ramsey (2021); r:5; Atmospheric data from Reimer et al (2020)
4Q267 R_Date(2151,21)
68.3% probability
(22.3%) 345-315calBCE
(46.0%) 205-155calBCE
95.4% probability
(28.6%) 355-290calBCE
(65.3%) 210-95calBCE
(1.6%) 70-55calBCE
Radiocarbon determination (BP)
Calibrated date (calBCE/calCE)

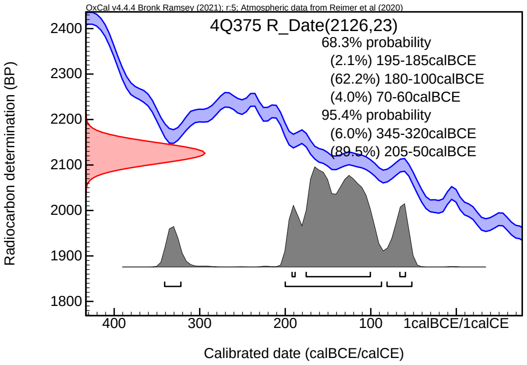
OxCal v4.4.4 Bronk Ramsey (2021); r:5; Atmospheric data from Reimer et al (2020)
4Q375 R_Date(2126,23)
68.3% probability
(2.1%) 195-185calBCE
(62.2%) 180-100calBCE
(4.0%) 70-60calBCE
95.4% probability
(6.0%) 345-320calBCE
(89.5%) 205-50calBCE
Radiocarbon determination (BP)
Calibrated date (calBCE/calCE)

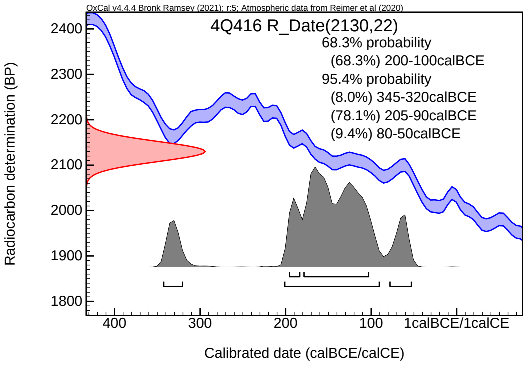
OxCal v4.4.4 Bronk Ramsey (2021); r:5; Atmospheric data from Reimer et al (2020)
4Q416 R_Date(2130,22)
68.3% probability
(68.3%) 200-100calBCE
95.4% probability
(8.0%) 345-320calBCE
(78.1%) 205-90calBCE
(9.4%) 80-50calBCE
Radiocarbon determination (BP)
Calibrated date (calBCE/calCE)

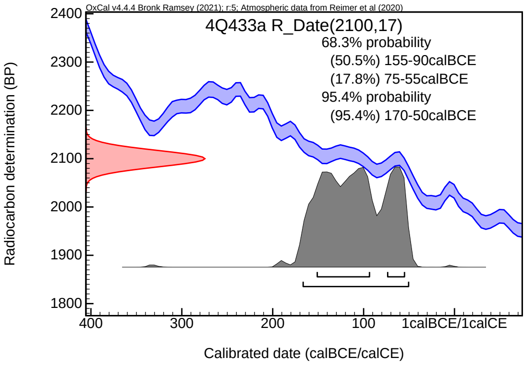
OxCal v4.4.4 Bronk Ramsey (2021); r:5; Atmospheric data from Reimer et al (2020)
4Q433a R_Date(2100,17)
68.3% probability
(50.5%) 155-90calBCE
(17.8%) 75-55calBCE
95.4% probability
(95.4%) 170-50calBCE
Radiocarbon determination (BP)
Calibrated date (calBCE/calCE)

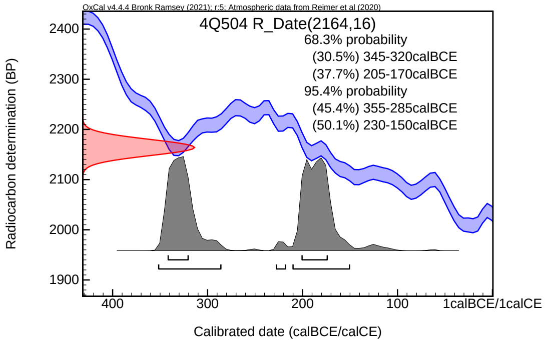
OxCal v4.4.4 Bronk Ramsey (2021); r:5; Atmospheric data from Reimer et al (2020)
4Q504 R_Date(2164,16)
68.3% probability
(30.5%) 345-320calBCE
(37.7%) 205-170calBCE
95.4% probability
(45.4%) 355-285calBCE
(50.1%) 230-150calBCE
Radiocarbon determination (BP)
Calibrated date (calBCE/calCE)

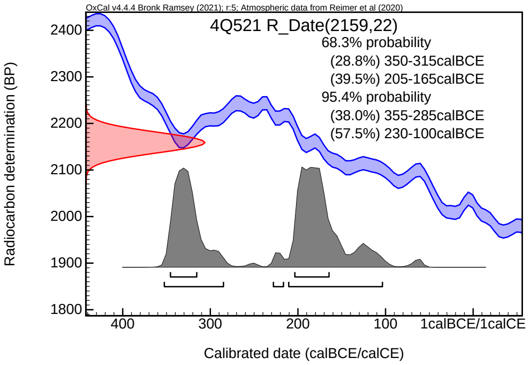
OxCal v4.4.4 Bronk Ramsey (2021); r:5; Atmospheric data from Reimer et al (2020)
4Q521 R_Date(2159,22)
68.3% probability
(28.8%) 350-315calBCE
(39.5%) 205-165calBCE
95.4% probability
(38.0%) 355-285calBCE
(57.5%) 230-100calBCE
Radiocarbon determination (BP)
Calibrated date (calBCE/calCE)

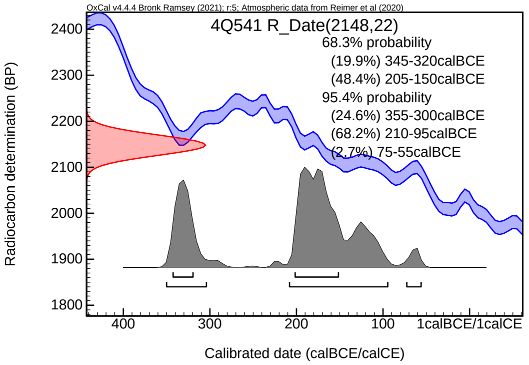
OxCal v4.4.4 Bronk Ramsey (2021); r:5; Atmospheric data from Reimer et al (2020)
4Q541 R_Date(2148,22)
68.3% probability
(19.9%) 345-320calBCE
(48.4%) 205-150calBCE
95.4% probability
(24.6%) 355-300calBCE
(68.2%) 210-95calBCE
(2.7%) 75-55calBCE
Radiocarbon determination (BP)
Calibrated date (calBCE/calCE)

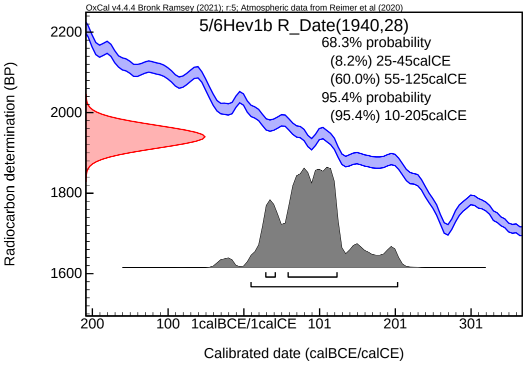
OxCal v4.4.4 Bronk Ramsey (2021); r:5; Atmospheric data from Reimer et al (2020)
5/6Hev1b R_Date(1940,28)
68.3% probability
(8.2%) 25-45calCE
(60.0%) 55-125calCE
95.4% probability
(95.4%) 10-205calCE
Radiocarbon determination (BP)
Calibrated date (calBCE/calCE)

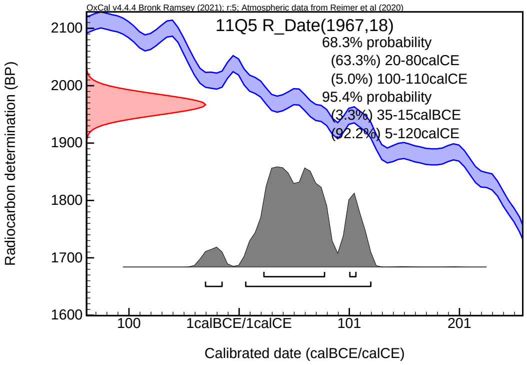
OxCal v4.4.4 Bronk Ramsey (2021); r:5; Atmospheric data from Reimer et al (2020)
11Q5 R_Date(1967,18)
68.3% probability
(63.3%) 20-80calCE
(5.0%) 100-110calCE
95.4% probability
(3.3%) 35-15calBCE
(92.2%) 5-120calCE
Radiocarbon determination (BP)
2100
2000
1900
1800
1700
1600
100
1calBCE/1calCE
101
201
Calibrated date (calBCE/calCE)

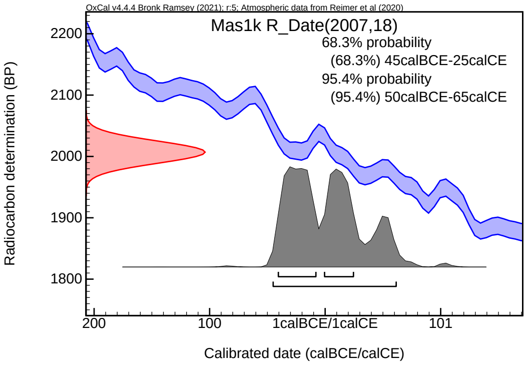
OxCal v4.4.4 Bronk Ramsey (2021); r:5; Atmospheric data from Reimer et al (2020)
Mas1k R_Date(2007,18)
68.3% probability
(68.3%) 45calBCE-25calCE
95.4% probability
(95.4%) 50calBCE-65calCE
Radiocarbon determination (BP)
2200
2100
2000
1900
1800
200
100
1calBCE/1calCE
101
Calibrated date (calBCE/calCE)

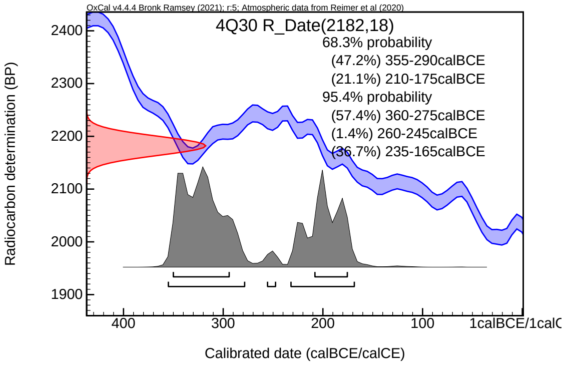
OxCal v4.4.4 Bronk Ramsey (2021); r:5; Atmospheric data from Reimer et al (2020)
4Q30 R_Date(2182,18)
68.3% probability
(47.2%) 355-290calBCE
(21.1%) 210-175calBCE
95.4% probability
(57.4%) 360-275calBCE
(1.4%) 260-245calBCE
(36.7%) 235-165calBCE
Radiocarbon determination (BP)
2400
2300
2200
2100
2000
1900
400
300
200
100
1calBCE/1calC
Calibrated date (calBCE/calCE)

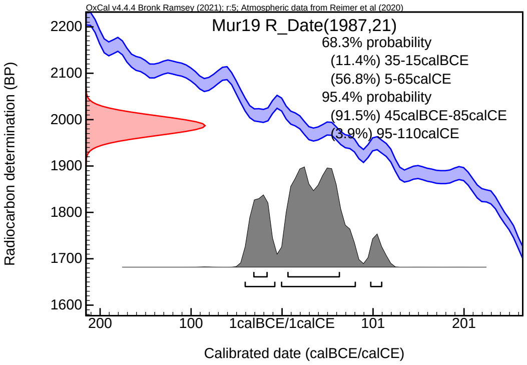
OxCal v4.4.4 Bronk Ramsey (2021); r:5; Atmospheric data from Reimer et al (2020)
Mur19 R_Date(1987,21)
68.3% probability
(11.4%) 35-15calBCE
(56.8%) 5-65calCE
95.4% probability
(91.5%) 45calBCE-85calCE
(3.9%) 95-110calCE
Radiocarbon determination (BP)
2200
2100
2000
1900
1800
1700
1600
200
100
1calBCE/1calCE
101
201
Calibrated date (calBCE/calCE)
