# Supplementary material for: Dating ancient manuscripts using radiocarbon and AI-based writing style analysis
Source: PLoS One. 2025 Jun 4;20(6):e0323185. doi: 10.1371/journal.pone.0323185 (PMC12136314; doi:10.1371/journal.pone.0323185)
Supplement: S4 Appendix — (PDF) [file pone.0323185.s004.pdf]

S4 Appendix for the article:

Dating ancient manuscripts using radiocarbon and AI-based writing style analysis

Mladen Popović[1*], Maruf A. Dhali[1,2], Lambert Schomaker[2], Johannes van der Plicht[3], Kaare Lund Rasmussen[4], Jacopo La Nasa[5], Ilaria Degano[5], Maria Perla Colombini[5], Eibert Tigchelaar[6],

1 Qumran Institute, University of Groningen, 9712 GK, The Netherlands
2 Artificial Intelligence, Bernoulli Institute, University of Groningen, 9747 AG, The Netherlands
3 Center for Isotope Research, University of Groningen, 9747 AG, The Netherlands
4 Department of Physics, Chemistry, and Pharmacy, University of Southern Denmark, DK 5230, Denmark
5 Department of Chemistry and Industrial Chemistry, University of Pisa, 56126 Pisa PL, Italy
6 Faculty of Theology and Religious Studies, KU Leuven, 3000 Leuven, Belgium

* m.popovic@rug.nl

Data and materials: All data, code, and test film associated with this article are publicly available on Zenodo with the following DOIs:

- Data and prediction plots (v3): https://doi.org/10.5281/zenodo.10998958.
- Code and feature files (v6): https://doi.org/10.5281/zenodo.13319794.
- Film (see details in S7 Appendix: https://doi.org/10.5281/zenodo.8167946.

Please note that this article has 12 appendices in total, from **S1** to **S12**.

S4 Palaeography and radiocarbon dating of the Dead Sea Scrolls

S4.1 Comparing radiocarbon results and palaeographic estimates

We make the comparison between the radiocarbon dates (Table S1) and previous palaeographic estimates on the basis of the estimates given in the official publication series, Discoveries in the Judaean Desert (DJD), as these are considered the standard in the field, but sometimes we include references to estimates of other scholars when relevant.

However, we also critically assess previous palaeographic estimates. We do that on two levels. First, we reason according to the relative typology of the so-called Cross model and assess its application to individual manuscripts. This leads occasionally to palaeographic assessments that correct previous ones. Second, we desist from translating a relative typology to an absolute chronology. Because of the lack of date-bearing documents for the time-period one cannot impose the traditional framework's unsubstantiated chronological limitations on when the so-called Hasmonaean and Herodian script features would have started to develop (see appendix S1.2.2). This also applies to chronological distinctions within the general indications of Hasmonaean-type and Herodian-type scripts. Cross suggested chronological ranges of 50 years, and sometimes even shorter ranges of 25–50 years, as he assumed a rapid development of the script from the Hasmonaean period onward, contrary to the presumed slow development in the third century BCE. However, this assumption of a rapid evolution remains unsubstantiated, too (see below). As more reliable time markers, our study's ^{14}C calibrated ranges demonstrate older date ranges than previously thought for individual manuscripts as well as for the beginnings of the Hasmonaean/Herodian scripts.

We can compare the radiocarbon dates with previous palaeographic estimates only in a general sense, not as a rigid application of these estimates. The early 1990s guideline that editors of manuscripts in the DJD series would date according to the typological specimens of Cross's 1961 article has proved unfortunate. One problem is that many of the palaeographic estimates offered in the DJD series since the 1990s suffer from an insufficient understanding of Cross's model, producing unreliable estimates [1]. This unreliability is further exacerbated by the problems within Cross's palaeographic model that conflates supposed historical and political developments with palaeographic style developments.

Cross [2] presented few specimens for other scholars to work with. This makes it difficult to substantiate style developments within, for example, Hasmonaean formal script, and to account for the complexity of script in individual manuscripts. Moreover, Cross also suggested mutual influences between formal, semiformal, and semicursive in such a way that sometimes a typological development of an individual letter is thought to have occurred earlier in, e.g., semicursive than in formal script (e.g., for *samek*).

For the Hasmonaean formal script Cross [2, 3] singled out only three manuscripts, assuming absolute dates between ca. 175–30 BCE: 4Q28, 4Q30, and 4Q51 (for a number of individual letters, Cross also referred to 1QIsa^{a} and 4Q1, as respectively middle and early Hasmonaean formal, as well as to 4Q109 and 4Q504 as early Hasmonaean semiformal). Thus, 4Q30 is said to be "a typical Hasmonaean" script, without explanation why that is so, from the middle of the period, 125–100 BCE (Cross might have used 1QIsa^{a} instead but because he deemed it to have more idiosyncratic forms he gave preference to 4Q30 which he understood to have been copied by a more conventional scribe; no further substantiation is provided for these claims). The other two manuscripts are at the outer ends of the Hasmonaean formal script spectrum, apparently for having script style elements in common with earlier and later periods. So 4Q28 is presented as transitional between Archaic and the beginning of the Hasmonaean development (175–150 BCE) and 4Q51 as a late transitional script from the end of the Hasmonaean period or the beginning of the Herodian period (50–25 BCE).

For the Herodian formal script Cross singled out seven manuscripts, assuming absolute dates between 30 BCE and 70 CE: 1QM, 4Q27, 4Q37, 4Q85, 4Q113, 5/6Hev1b, and Mur24. In fact, only four manuscripts are singled out for the Herodian formal script. 1QM is presented as "a typical early Herodian formal script" (ca. 30–1 BCE), while 4Q113 would represent "a developed Herodian formal script" (20–50 CE) and 4Q37 and 4Q85 late Herodian formal scripts from respectively ca. 50 CE and ca. 50–68 CE. 4Q27 is said to be "a typical exemplar of the extremely popular Round semiformal style" (also called rustic, and considered distinct from the Vulgar semiformal) from the early Herodian period, ca. 30 BCE–20

CE. The final two manuscripts are actually considered so-called post-Herodian, assuming absolute dates between 70–135 CE: 5/6Hev1b was estimated by Cross from 75–100 CE (Flint suggested 50–68 CE [4]) and Mur24 is a date-bearing document from 133 CE. Cross saw in some Herodian scripts the types of individual letters mixed so that semiformal can "invade" formal or Vulgar semiformal "makes its way into the formal character", e.g., *mem*,

Apart from evaluating individual letters, there is no method in the field for dating an entire manuscript on the basis of mixed evidence of 'older' and 'later' forms of individual letters. Perhaps some scholars apply a form of quantification, weighing the instances of 'older' and 'later' forms, but this is never explicated. Rather, the assumption generally seems to be that 'later' forms cannot have developed earlier but 'older' forms can still have been in use at a later time, whether or not as a case of 'archaizing'. While it may certainly be true that 'older' forms can have been in use for a long time, the claim that 'later' forms cannot have developed earlier remains unproven for lack of dated evidence. This means that what are perceived as, e.g., late Hasmonaean or early Herodian letter forms may have developed earlier than currently thought.

Even if one adopts Cross's typological development, the issue of the absolute dating or calibration of the types remains [1]. A mixture of 'older' (more ancient) and 'later' (more developed) forms can appear in one and the same manuscript. A focus on individual letters alone cannot be indicative for earlier or later chronology, whether relative or absolute. The study of individual manuscripts demonstrates a more complex development (see, e.g., for 4Q1 [5]). There are examples of experienced palaeographers coming up with widely diverging dates for the same scrolls. Thus, a range of individual manuscripts cannot be fitted precisely in a sequence on the basis of traditional palaeography.

The radiocarbon dates and the palaeographic estimates are two independent information sources about history, based on two different methodologies: one is a physically measured "yardstick of time", the other is a cultural and qualitative assessment. At present, in the absence of an abundance of date-bearing manuscripts between the third century BCE and the first century CE, radiocarbon dates (^{14}C) derived from manuscript samples are more reliable time markers. The palaeographic estimates do not provide absolute or fixed dates.

With these caveats in mind, Fig 1 in the main article shows the comparison between the (accepted) 2σ calibrated ranges and previous palaeographic estimates (see the worksheet in appendix S12 for the specific data and information). Additional plots can be found in appendix S8 where Fig S26 and S27 presents the effect of including or excluding minor peaks to the 2σ calibrated ranges and Fig S28 presents the outcome of selecting 1σ calibrated range.

S4.1.1 Whole or partial overlap

Comparing previously given palaeographic estimates and our ^{14}C 2σ results, shows that 17 of the 26 sampled manuscripts in our project have whole or partial overlap. This applies to: 4Q23, 4Q47, 4Q52, 4Q70, 4Q161, 4Q176, 4Q201/4Q338, 4Q255/4Q433a, 4Q259, 4Q504, 4Q521, 4Q541, 11Q5, Mas1k, Mur19, 5/6Hev1b, XHev/Se2.

4Q47 is a good example of how palaeographic estimates cannot be precise or clearly substantiated. Ulrich [6] reports that Cross had identified its script as Hasmonaean—thus dating it probably in the second half of the second century or the first half of the first century BCE—but refrained from offering a more precise estimate within the Hasmonaean period. Langlois [7] and Puech [8] favoured the first half of the first century BCE. Langlois referred to some letters showing a typologically older form (*bet*, *dalet*, *vav*, *khet*, *nun*), while others would have a form more in line with those seen in late Hasmonaean or early Herodian periods (*aleph*, *he*, *tet*, *samek*, *pe*). However, considering, e.g., *aleph* one can see two forms, one of them being a typologically older form where the left leg often connects to the middle of the diagonal instead of more toward the top of it; the same for *samek* that appears in both closed (younger) and open (older) form. Also, *ayin* is often small, considered an older form, while *yod* shows the triangular head, seen as typical for the late Hasmonaean period. Instead of trying to fit this manuscript overall into a linear date estimate, the mixed typological evidence can be better explained as demonstrating overlapping or partly adjacent style developments.

In appendix S2.4 we noted that our calibrated results are often bimodal, especially for 2σ distributions. 4Q47 is an example of such bimodal calibrated results, also for the 1σ distribution. The ^{14}C 2σ calibrated range of 210–100 BCE (61.6% probability) overlaps with the broad palaeographic estimate 'Hasmonaean'—but less with the more specific ones of Puech and Langlois—and also allows for dating the script style of 4Q47 to the first half of the second century BCE.

The older 2σ calibrated range of 355–290 BCE (33.8%) is far removed on the timeline from previous palaeographic estimates. Although the older 2σ peak represents a mathematically valid solution of the dating process, the younger calibrated peak must be preferred for 4Q47 over the older calibrated peak. Following the palaeographic principle to compare the script of an undated manuscript to that of dated writings with a similar script (see appendix S1.2.2), it should be noted that 4Q47 does not compare to the extant typological evidence from date-bearing Aramaic manuscripts from the fourth century BCE. Typologically, the script of 4Q47 does not correspond to that of the script in date-bearing documents from the Persian period such as those from Bactria or from Wadi Daliyeh from the same region as the Dead Sea Scrolls. So, from a palaeographic perspective, 4Q47 is clearly younger than where the older calibrated peaks appear on the timeline.

Prior to the discovery of the Wadi Daliyeh documents, 4Q52 was argued by Cross to be the oldest manuscript among the Dead Sea Scrolls, and it certainly has the best cards for being the oldest biblical manuscript. In the official publication, Cross et al. estimated 4Q52 to ca. 250 BCE [9].

The ^{14}C evidence is bimodal for the 2σ distribution. The younger peak of 285–230 BCE (16.6% probability) agrees well with the palaeographic estimate. The older calibrated range is 410–355 (78.9%). Although it cannot be ruled out completely from a palaeographic perspective, this older date seems typologically slightly too early for the script in 4Q52 in comparison to date-bearing documents from Elephantine from the late fifth century BCE and date-bearing documents from Bactria from 353 to 324 BCE, although it is difficult to factor in consequences of geographical variance for script variations. A date range in the second half of the fourth century BCE would seem more suitable for 4Q52. Following palaeographic principle, 4Q52 would have to be dated chronologically nearer to the Wadi Daliyeh manuscripts, especially WDSP 1 from 335 BCE (see appendix S1.2.2). But for that date range there is no ^{14}C result.

Hence, from a palaeographic perspective a clear preference for one of the two peaks in the probability distribution cannot be substantiated. The 2σ range of 410–355 is perhaps only a few decades too old and not one to two centuries as for most other bimodal results of our ^{14}C measurements. So, in the case of 4Q52, the older peak cannot be rejected as a possible solution with as much confidence as for most other ^{14}C samples with bimodal evidence.

4Q176 has two script styles (plausibly from two scribes): the script of fragment 1–2 i looks entirely different from 1-2 ii. The ^{14}C sample in this study was taken from fragment 1-2 ii. Strugnell [10] and Tigchelaar [11] characterized its script as 'middle Hasmonaean', i.e., ca. 125–75 BCE. Strugnell's palaeographic analysis can be easily misunderstood. He explains that many of the letter forms of the second script style seem to be Herodian, such as *bet*, *tet*, *mem*, and *qoph*. Yet, because the script is not formal but semiformal these forms must be dated to the middle Hasmonaean period. Fragment 1-2 ii shows less uniformity in size than fragment 1-2 i, e.g., *kaph* or medial *mem*. This can be understood as a typologically older feature where *kaph* and medial *mem* are still larger other than letters. The ideal of a base line seems not yet well developed. The three-stroke *he* and the small-sized *ayin* seem archaic. On the other hand, the *bet* has a broad base stroke and protrudes to the right, in formal script generally typologically connected to late Hasmonaean or early Herodian. But if the distinction between formal and semiformal cannot be clearly made, 4Q176 is another example of mixed evidence.

4Q176 is another example of bimodal calibrated results, having in addition also minor peaks of low probability. The 2σ calibrated range of 210–100 BCE (64.2% probability) and the minor peak of 70–60 BCE (0.7%) are consistent with previous palaeographic assessments. The 2σ calibrated range of 210–100 BCE also makes an older dating of the script style possible.

These assessments for 4Q47, 4Q52, and 4Q176 also apply to 4Q23, 4Q70, 4Q161, 4Q255/4Q433a, 4Q259, 4Q504, 4Q521, 4Q541, Mas1k, and XHev/Se2. Only in the case of 4Q201 and 11Q5 do the ^{14}C results indicate a date range that goes in the direction of a younger possible date, whereas in almost all

cases the direction is toward an older possible date range.

Regarding 4Q201, Milik's edition [12] suggested the first half of the second century BCE, and most scholars have accepted this estimate. He considered its script to be quite archaic and connected to the third and second-century BCE semicursive or semiformal scripts, perhaps more dependent on the Aramaic writing of northern Syria or Mesopotamia than on those of Judaea or Egypt. Similar comparisons with northern Syria have been made for 4Q17 and 4Q109, but concrete connections cannot be substantiated. Puech [13] also saw the script as semiformal/semicursive, dating from ca. 200 BCE, while Langlois [7] gave an estimate of ca. 150 BCE.

4Q201 has a 2σ calibrated range of 165–40 BCE (93.6%) and a minor peak of 10–1 BCE (1.9%). This overlaps with the palaeographic estimates, but instead of an older date, a younger date than previously considered is also possible.

The script of 4Q201 is hard to assess, in part because the scribe used a pen with a thick, worn nib to write small letters, which may account for the atypical *aleph*. Yet, apart from archaic forms of *samek* and *shin* nothing is typologically incongruent with the early Hasmonaean script.

As for 11Q5, Sanders [14] understood its script as transitional from early to late Herodian, comparing it to 4Q113 and also 1QM, 4Q27, 4Q37, and 4Q51. He estimated its script to the first half of the first century CE, possibly slightly earlier than 4Q113, Cross's specimen for "a developed Herodian formal script". However, clear typological distinctions on the level of individual letters between 'early', 'developed', and 'late' Herodian according to Cross's specimens are not that easily made. For example, one may consider *aleph* which from early to late Herodian would advance to an inverted "v" form of the left leg and oblique axis, or *dalet*, where the horizontal stroke breaks through the right leg, and see that there is no difference here between the 'developed' and 'late' Herodian specimens of 4Q113, 4Q37, and 4Q85. On the other hand, the sharp bent in the right leg of *ayin* and *sin*/*shin* may be seen in early as well as developed and late Herodian exemplars, whereas in some manuscripts that are considered to be late Herodian the sharp bent is not clearly shown, e.g., Mur88 and 5/6Hev1b. As to more general features of the Herodian formal script, one may consider a generally uniform letter size, a base line, ligatures, and the development of *keraiai* or serifs. But beyond a general impression, these features are difficult to use for a clear typological differentiation of manuscripts within the Herodian formal script.

11Q5 has a 2σ calibrated range of 5–120 CE (92.2%) and a minor peak of 35–15 BCE (3.3%), showing clear overlap with the different presumed Herodian palaeographic periods, even post-Herodian. The measurement has a standard deviation of only 18 in ^{14}C years (BP). The length of the 2σ calibrated range, 35 BCE–120 CE, is caused by the shape of the calibration curve in this period when converting the BP dates to calendar dates. Scholars of the Dead Sea Scrolls may consider a date later than 70 CE for 11Q5 unlikely because the scrolls found in the Qumran caves are assumed to have been hidden in the summer of 68 CE [15].

4Q259 is notorious for its widely varying palaeographic estimates in the second-first centuries BCE. Cross [16] described 4Q259 as written in an unusual semicursive with mixed semicursive and semiformal script features. He gave 50–25 BCE as a date estimate. Earlier, Milik [12] had suggested the second half of the second century BCE (Milik used the older reference number 4Q260), while later Puech [17] suggested the first half of the first century BCE, preferably shortly after 100 BCE. Puech argued for this date on a combined basis of a palaeographic analysis of Cryptic A script (compared to 4Q298 and especially to 4Q249 and 4Q317) and the ^{14}C dating of 4Q317 [18].

4Q259 has a 2σ calibrated range of 210–100 BCE (69.7%) and a minor peak of 70–55 BCE (1.4%). The 2σ calibrated range of 210–100 BCE agrees with the two older palaeographic estimates of Milik and Puech, whereas the minor peak of 70–55 BCE is nearer to Cross's estimate. The bimodal evidence for 4Q259 shows an older 2σ calibrated range of 350–310 BCE (24.3%), but, as for 4Q47, this older peak can be rejected as possible solutions based on typological comparison with date-bearing Aramaic manuscripts from the fourth century BCE.

Following Cross's typology, Puech [19] analysed 4Q521 as a Hasmonaean formal script and estimated it between 100–80 BCE. This manuscript was also radiocarbon dated in the 1990s [18]. That BP date (1984 $\pm$ 33) now has to be recalibrated according to the IntCal20 calibration curve ([20]), which results in a 2σ date range of 45 BCE–120 CE. According to the bimodal evidence of our study, the younger 2σ

calibrated range is 230–100 BCE (57.5%), while the older peak in the 2σ range of 355–285 BCE (38.0%) can be rejected as a possible solution due to comparative typological evidence from date-bearing Aramaic manuscripts from that period. The difference in age between the two radiocarbon tests may be due to the Soxhlet procedure cleaning castor oil from the sample, but it is not possible to quantify or ascertain that. The palaeographic estimate of 100–80 BCE and our 2σ calibrated range of 230–100 BCE connect in the year 100 BCE. So, considering measurement uncertainties, 4Q521 can be taken as a partial overlap.

The script of 5/6Hev1b was considered by Cross [2] to be a post-Herodian formal, estimated from 75–100 CE (Flint [4] suggested 50–68 CE). This sample was the least precise ^{14}C result in our study, with a standard deviation of 28 years in BP, and calibrated in 2σ to 10–205 CE. The large calibrated date range, caused by the shape of the calibration curve in this period, clearly encompasses previous palaeographic estimates, but also moves in both a much older and a much younger direction of possible dates.

S4.1.2 No overlap

Nine out of 26 samples yield (accepted) 2σ calibrated ages that do not overlap with previous palaeographic estimates. In all 9 cases, the ^{14}C results give calibrated age ranges that are older than previous palaeographic estimates. Yet, in light of our critical assessment, the older ^{14}C age ranges are in most cases also palaeographically possible and realistic. This applies to: 4Q2, 4Q3, 4Q27, 4Q30, 4Q114, 4Q206, 4Q267, 4Q375, 4Q416.

4Q30 was Cross's "typical Hasmonaean" script specimen from the middle of the period, 125–100 BCE, like 1QIsa[a] [2]. The calibrated result for this sample in our study is bimodal. According to the ^{14}C measurement, 4Q30 has a 2σ calibrated range of 235–165 BCE (36.7%) and a minor peak of 260–245 BCE (1.4%). The older 2σ peak of 360–275 BCE (57.4%) can be rejected as a possible solution based on palaeographic comparison with date-bearing manuscripts in Aramaic script from the period. Though Cross gave the more narrow estimate from 125–100 BCE, White Crawford estimated more broadly from 150–100 BCE [6]. An earlier date range, say in the first half of the second century BCE, as indicated by ^{14}C, is realistic and possible. In general, there is no reason to chronologically limit the script identified as Hasmonaean to the upper range of the political-historical period of the same name in the mid-second century BCE (see appendix S1.2.2). The sequence of relative typology can chronologically easily be moved to an older age range. Though in general 4Q30 shows a more uniform letter size, at the level of individual letters, the often not yet 'standard' letter size of *aleph* and the often small *ayin* point to earlier typology in the Hasmonaean script. As we also argued for, for example, 4Q47 and 4Q176 (appendix S4.1.1), 4Q30 shows mixed typological evidence.

4Q27 was Cross's "typical exemplar of the extremely popular Round semiformal style", initially estimated by him to be early Herodian (ca. 30 BCE–20 CE) but later slightly revised by Jastram and Cross to the latter half of the first century BCE [21]. 4Q27 has a 2σ calibrated range of 200–50 BCE (94.2%) and a minor peak of 340–330 BCE (1.3%) that can be rejected as a possible solution for palaeographic reasons. The 2σ calibrated range of 200–50 BCE comes near the revised palaeographic estimate. The calibrated date has a large range. This is caused by the measurement's standard deviation of 26 ^{14}C years (BP) in combination with the shape of the calibration curve in this period.

Interestingly, the 2σ calibrated range for another specimen of the Herodian round semiformal, 4Q161, is 55 BCE–30 CE (92.1%), with two minor peaks of 90–80 BCE (1.7%) and 45–60 CE (1.7%). This may suggest a longer and somewhat older age range for this Herodian-type script than only the latter half of the first century BCE. Palaeographically, there are also many differences between 4Q27 and 4Q161. In 4Q161 the long extending base strokes of *kaph*, broad *dalet*, ligatures, and strikingly penned *tet* and *shin* stand out, whereas 4Q27 shows less tendency to broadening of letters. Although possible from a ^{14}C perspective, a date in the first half of the second century BCE for 4Q27 seems unlikely from a typological perspective in comparison with other manuscripts. Nonetheless, there are four more Herodian-type manuscripts dated to that range by ^{14}C in our study: 4Q3, 4Q267, 4Q375, and 4Q416.

4Q267 is another example of Cross's early Herodian round semiformal. Yardeni related 4Q267 to 4Q397 as possibly written by the same scribe and estimated it from 30 BCE–20 CE [22] (Yardeni did not take over Cross's round semiformal categorization and understood its script as formal). 4Q267 was also

radiocarbon dated in the 1990s [18]. That BP date (2094 ± 29) now has to be recalibrated according to the IntCal20 calibration curve ([20]), which results in a 2σ calibrated range of 200–40 BCE (94.0%) and a minor peak of 10 BCE–5 CE (1.5%). According to our study, 4Q267 has a 2σ calibrated range of 210–95 BCE (65.3%) and a minor peak of 70–55 BCE (1.6%), whereas the older 2σ range of 355–290 BCE (28.6%) can be rejected as a possible solution due to comparative typological evidence from date-bearing Aramaic manuscripts from that period. The difference in age between the two radiocarbon tests may by due to the Soxhlet procedure cleaning castor oil from the sample, but it is not possible to quantify or ascertain that.

From a typological perspective it is difficult to understand the script of 4Q267 being chronologically so near to quite different typological specimens in the second century BCE. We may have to reckon with overlapping or partly adjacent style developments but in this case it would severely impact the relative typology dominant in the field, not just moving it chronologically and keeping the relative typology intact.

4Q267 might be an outlier, yet this ^{14}C result raises the fundamental issue of how the absolute, chronological dating of typological differences in a linear sequence has been substantiated. Cross assumed a slow development of the Aramaic/Hebrew script in the third century BCE and he assumed a rapid evolution of the script in the Hasmonaean and Herodian eras, but he could not substantiate either assumption, due to the lack of date-bearing documents. He assumed but did not demonstrate that the finer typological distinctions had to be chronologically sequenced one after the other instead of existing partially next to each other (see, similarly, [23]).

Cross wavered with his palaeographic estimate of the 'semicursive' 4Q114 from the late second century BCE (125–100) to ca. 100–50 BCE, and, under influence of the finds of Wadi Daliyeh, back to the late second century BCE [24], "no more than about a half century younger than the autograph", Cross said [25]. Interestingly, Cross dated 4Q114 contemporary to the formal hand of 4Q30. 4Q114 preserves Daniel 8–11, a part of the book which scholars argue on literary-historical grounds to have been composed in the 160s BCE. 4Q114 has a 2σ calibrated range of 230–160 (45.9%) and an older 2σ range of 355–285 (49.5%) that can be rejected as a possible solution based on comparative typological evidence from date-bearing Aramaic manuscripts from that period.

Because of its scribal errors, it is unlikely that the scribe of 4Q114 was the author. But the early date and low scribal quality of 4Q114 shed new light on the production and circulation of literature in ancient Judaea: its date is indicative for the speed of the text's spread, and the low quality of the manuscript may indicate it originated in a social context close to the original author [26]; future research may further validate this. 4Q114 would then have been copied very soon after the assumed composition of Daniel 8–11. The ^{14}C 2σ date of 230–160 BCE for 4Q114 is matched by a very much comparable older ^{14}C date of 4Q30.

For 4Q206, Milik [12] gave an estimate from the first half of the first century BCE, and simply referred to four of the exemplary Hasmonaean manuscripts given by Cross (4Q30, 4Q51, 4Q114, 4Q398), apparently with no concern for their differences in style and for Cross dating these quite differently. In his recent edition in consultation with Puech, Drawnel [27] estimated 4Q206 to be from the middle of the first century BCE. It is interesting that two of Milik's typological comparanda, 4Q30 and 4Q114, have ^{14}C results in our study similar to 4Q206: the 2σ calibrated range for 4Q206 is 235–145 BCE (45.8%) with a minor peak of 135–120 BCE (1.1%); the older 2σ range of 360–280 BCE (48.6%) can be rejected as a possible solution for palaeographic reasons. In each of these cases the ^{14}C results indicate an earlier chronological date than the palaeographic estimates. But typologically some letters are slightly different and commonly seen as a later development of the letter form, e.g., *bet*, *mem*, and *ayin*. Yet, other letters show varied forms within 4Q206 and some compare well with instances from 4Q30, e.g., *aleph*, *he*. So 4Q206 may be another example of mixed typological evidence.

Then there are four Herodian-type manuscripts whose ^{14}C dates extend into the second century BCE: 4Q2, 4Q3 and 4Q375 and 4Q416.

The script of 4Q2 has been described as late Herodian or even post-Herodian (ca. 50–68+ CE), in part because of the increasing use of *keraiai* [21]. While the script is typologically certainly Herodian, the assumption that calligraphic features are typical for its latest period cannot be substantiated. 4Q2 has a

2σ calibrated range of 125 BCE–10 CE (90.3%) with a minor peak of 155–130 BCE (5.2%), providing a date range up to 10 CE, which seems realistic to us.

The case of 4Q3 is more difficult. Its script was tersely described as "an Herodian formal hand dating from the middle to end of that period (c. 20-68 CE)" [21]. Indeed, the script of 4Q3 features several letters and elements generally regarded to be developed Herodian, like the small tick above the crossbar of the final *mem*. Yet, some letters have older shapes which are uncommon in those developed Herodian formal hands, such as the 'horned' *dalet*. 4Q3 has a 2σ calibrated range of 200–50 BCE (92.0%) and a minor peak of 340–325 BCE (3.5%) that can be rejected as a possible solution for palaeographic reasons. The palaeographic rule of thumb that the latest forms are indicative for its age would militate against the 2σ range of 200–50 BCE. Yet, the exact moment when those latest forms have arisen has not been substantiated in the field. Future evidence may further validate this.

Strugnell provided a judicious analysis of the palaeography of 4Q375, comparing its style to that of the round or rustic semiformal series which is generally associated with early Herodian, but also arguing that, typologically, it must be an early exemplar since some letters do not yet have the typically Herodian forms [28]. True to his custom, he did not translate this typological assessment into a calendar date, but in Cross's correspondence between hand and style this would amount to ca. 50–25 BCE, which would nearly agree with the 2σ calibrated range of 205–50 BCE (89.5%). Considering the uncertainties in the palaeographic estimate, this is acceptable. The 2σ peak of 340–320 BCE (6.0%) can be rejected as a possible solution for palaeographic reasons.

Also for 4Q416, Strugnell carefully analysed its individual letters, arguing that in most cases these should be placed between 4Q51 and 1QM, hence "in a date transitional between the late Hasmonaean and the earliest Herodian hands" [29]. He judged the script of 4Q416 to be earlier than those of 4Q415, 4Q417, and 4Q418 by some twenty-five years so that a palaeographic estimate of 50–25 BCE presents itself. 4Q416 has a 2σ calibrated range of 205–90 BCE (78.1%) and a smaller peak of 80–50 BCE (9.4%). Considering the uncertainties in the palaeographic estimate, this is acceptable. The 2σ peak of 345–320 BCE (8.0%) can be rejected as a possible solution for palaeographic reasons.

S4.1.3 Concluding the comparison between radiocarbon results and palaeographic estimates

Based on this comparison between (accepted) 2σ calibrated dates and previous palaeographic estimates we make the following concluding observations.

Overall, the ^{14}C results indicate older date ranges for individual manuscripts. Only two manuscripts, 4Q201 and 11Q5, have date ranges that go in the direction of a younger possible range (5/6Hev1b has a range both a bit older and much younger). Thus, Hasmonaean-type manuscripts have ^{14}C date ranges that allow for older dates in the first half of the second century BCE, and sometimes also up to the latter part of the third century BCE, instead of the late second century or early first century BCE. There are no compelling palaeographic or historical reasons that preclude these older dates as reliable time markers for the Hasmonaean script (this also applies to the solid third-century BCE range for 4Q70 and its Archaic-type script).

The ^{14}C results for most manuscripts confirm the basic distinction between older Hasmonaean-type manuscripts and younger Herodian-style manuscripts, and, for that matter, also between Archaic-type (4Q52 and 4Q70) and Hasmonaean-type manuscripts. However, the ^{14}C date ranges for manuscripts that are traditionally considered Hasmonaean and Herodian are quite differently distributed across the timeline.

As can be seen in Fig 1 in the main article, the twelve Hasmonaean-type manuscripts in our sample set have (accepted) 2σ calibrated date ranges from the second and first century BCE, as expected, and most extend also into the late third century BCE. Three Herodian-type manuscripts (4Q161, Mas1k, XHev/Se2) have (accepted) 2σ calibrated date ranges from the latter half of the first century BCE and the first century CE, as expected. Two Herodian-type manuscripts have date ranges in the first century CE, as expected, but also extend into the second century CE (11Q5 and 5/6Hev1b, the latter even into the early third century CE). 4Q2 has a date range extending from the early first century CE back to the early second century BCE. And five Herodian-type manuscripts have (accepted) 2σ calibrated date

ranges in the second century BCE (4Q3, 4Q27, 4Q267, 4Q375, and 4Q416), though 4Q27 extends into the first century BCE.

This adds a third component to our critical assessment. In addition to critiquing the application of traditional typology to individual manuscripts and dismantling unsubstantiated historical suppositions and chronological limitations, the results of this study also question the validity of the relative typology as such. The traditional relative typology can be maintained but not in all cases. The spread of the Hasmonaean-type manuscripts over the timeline does not affect Cross's relative typology in a major way but the older, second-century BCE date ranges of the Herodian-type manuscripts do affect the relative typology potentially in a major way.

Individual manuscripts frequently show mixed typological evidence: a manuscript can have different forms of individual letters that are considered 'older' or 'younger' according to the traditional palaeographic framework. There is, however, not a good method to assess what this means in terms of relative placement of the individual manuscript, nor, for that matter, what it means for relative typology in general. The rule of thumb that the typologically latest forms should determine one's palaeographical estimate of a manuscript, presupposes existing palaeographical date markers and a decision which features are typologically important or indicative. The dated Wadi Daliyeh discoveries showed that features that were supposed to be significantly later, already appeared many decades earlier than previously expected. Moreover, it is assumed but not substantiated that typological differences must be translated to chronological linear sequences. Instead of a linear development, as Cross and others have assumed, the possibility of overlapping or partly adjacent style developments must be considered.

So, the so-called Hasmonaean script can indeed be regarded as older than the so-called Herodian script but the ^{14}C results of this study indicate that the Herodian script was present earlier than previously thought. This suggests that these scripts were not transitioning from the mid-first century BCE onward (the so-called late Hasmonaean/early Herodian category of manuscripts) but that much earlier they already existed partially next to each other.

This study shows that there are no cogent reasons for limiting the palaeographic dating of style developments to political-historical periods such as Hasmonaean or Herodian. The terms 'Hasmonaean' and 'Herodian' might still be employed for types of script, but these cannot be converted to specific date ranges. For date estimations of individual manuscripts, one should rather use concrete age ranges.

S4.2 Applying palaeography to determine usable bimodal radiocarbon data input to train the artificial intelligence-based date-prediction model

In this study, only in the case of bimodal ^{14}C evidence have we used palaeographic knowledge to determine the usable radiocarbon dating input for the training of our date-prediction model (see 5.6). It should be stressed that this means a combination of qualitative and quantitative approaches and methods. Palaeography is a qualitative approach, based on expert knowledge, which is similar to the role of, for example, epigraphy as expert knowledge in [30]. This is different from, for example, an archaeological or geological stratigraphy that often can be quantified on the timeline. But the Dead Sea Scrolls do not have a similar stratigraphy in the archaeological record so that archaeological stratified sequence information is lacking, which means that we do not have the type of chronologically ordered data that normally goes into the OxCal program to perform further chronological modelling within that program (see appendix S5.6.2). From a palaeographic approach alone it is not possible to pinpoint an exact date, date range, or date limit on the timeline of the period of the Dead Sea Scrolls, because palaeographic dates are estimates and do not provide absolute or fixed dates. For example, in our research, palaeography tells us that most scrolls cannot date to the fourth century BCE but we cannot assign a limiting number like 300 BCE. Moreover, typological script development is a gradual process, not necessarily following a linear time trajectory.

Most Dead Sea Scrolls are typologically younger when compared to the script in Aramaic date-bearing documents from the fourth century BCE. Therefore, when any knowledgeable palaeographer is presented with the bimodal evidence in the 2σ range as is the case with our study, then they certainly will reject the older peak as a possible solution, as we have already explained (appendix S4.1.1 and S4.1.2), a

principle also applied by [18, 31]. Hence, in these cases of bimodal evidence typologically younger is also chronologically younger.

However, it is an open research question whether some of the oldest Dead Sea Scrolls might be older than their palaeographic estimated date in the mid-third century BCE. Following the palaeographic principle to compare the script of an undated manuscript to that of dated writings with a similar script (see appendix S1.2.2), we have already argued that 4Q52 would have to be dated chronologically nearer to the Wadi Daliyeh manuscripts, especially WDSP 1 from 335 BCE (see appendix S4.1.1). This may also apply to 4Q70, which has 2σ calibrated ranges of 320–200 BCE (79.2%) and 375–345 BCE (16.3%). The older 2σ peak can be rejected as a possible solution based on typological comparison but the younger 2σ peak's extending into the fourth century BCE cannot be completely ruled out, although 4Q70 is typologically further removed from the date-bearing Aramaic manuscripts from the fourth century BCE than 4Q52 and a date in the third century BCE for 4Q70 is more likely from a palaeographic perspective. However, such a qualitative assessment cannot be characterised as a specific quantitative prior (an expected date with mean and standard deviation) in the timeline. Therefore, palaeographers cannot give an exact date such as 280, 300, or 320 BCE as the year before which none of the Dead Sea Scrolls can be dated.

In order to train our artificial intelligence-based date-prediction model, we use the accepted 2σ calibrated data from 24 of the 26 valid ^{14}C results (Table S1). For the training of Enoch, the data from two manuscript samples are not used: Mur19 and 4Q52. Because of its cursive script, the papyrus fragment Mur19 is, at the moment, not relevant for Enoch. We also leave 4Q52 out of consideration because, in this case, we cannot decide between the two peaks in the probability distribution (see appendix S4.1.1). This is why we work with the tentative addition or deletion of 4Q52 in the training of our algorithm. This is how we get from 26 valid ^{14}C results to 24 manuscripts used as the primary training set for Enoch.

References

1. Tigchelaar E. Seventy Years of Palaeographic Dating of the Dead Sea Scrolls. In: Drawnel H, editor. Sacred Texts and Disparate Interpretations: Qumran Manuscripts Seventy Years Later. Leiden: Brill; 2020. p. 258–278.

2. Cross FM. The Development of the Jewish Scripts. In: Leaves from an Epigrapher's Notebook: Collected Papers in Hebrew and West Semitic Palaeography and Epigraphy. Winona Lake, IN: Eisenbrauns; 2003. p. 1–43.

3. Cross FM. Palaeography and the Dead Sea Scrolls. In: Flint PW, VanderKam JC, editors. The Dead Sea scrolls after fifty years: A comprehensive assessment, Volume one. Leiden: Brill; 1998. p. 379–402.

4. Charlesworth J, Cotton H, Flint P. Discoveries in the judaean desert: Volume XXXVIII. Miscellaneous texts from the judaean desert. Oxford: Clarendon Press; 2000.

5. Tigchelaar E. 4Q1 (4QGen-Exod^{a}): Identification of Fragments and Comments. Textus. 2023;32:19–38. doi:10.1163/2589255X-bja10028.

6. Ulrich E, Cross FM. Discoveries in the Judaean Desert: Volume XIV. Qumran Cave 4.IX. Deuteronomy, Joshua, Judges, Kings. Oxford: Clarendon Press; 1995.

7. Langlois M. Le premier manuscrit du Livre d'Hénoch: Étude épigraphique et philologique des fragments araméens de 4Q201 à Qumrân. Paris: Cerf; 2011.

8. Puech E. Les copies du livre de Josué dans les manuscrits de la mer Morte: 4Q47, 4Q48, 4Q123 et XJosué. Revue biblique. 2015;122:481–506. doi:10.2143/RBI.122.4.3149591.

9. Cross FM, Parry DW, Saley RJ, Ulrich E. Discoveries in the Judaean Desert: Volume XVII. Qumran Cave 4.XII. 1–2 Samuel. Oxford: Clarendon Press; 2005.

10. Strugnell J. Notes en marge du volume V des “Discoveries in the Judaean Desert of Jordan”. Revue de Qumran. 1970;7:163–276.

11. Tigchelaar E. Lamentations 4:21-22 as Another Word of Consolation in 4Q176. Revue de Qumran. 2019;31:3–9. doi:10.2143/RQ.31.1.3286503.

12. Milik JT. The books of Enoch: Aramaic fragments of Qumrân Cave 4. London: Clarendon Press; 1976.

13. Puech E. La paléographie des manuscrits de la mer Morte. In: Fidanzio M, editor. The Caves of Qumran. Leiden: Brill; 2017. p. 96–105.

14. Sanders JA. Discoveries in the judaean desert: Volume IV. The psalms scroll of Qumran cave XI. Oxford: Clarendon Press; 1965.

15. Popović M. Qumran as Scroll Storehouse in Times of Crisis? A Comparative Perspective on Judaean Desert Manuscript Collections. Journal for the Study of Judaism. 2012;43:551–594. doi:10.1163/15700631-12341239.

16. Charlesworth JH, Milgrom J, Qimron E, Schiffmann LH, Stuckenbruck LT, Whitaker RE, editors. The dead sea scrolls. Hebrew, Aramaic, and Greek texts with English translations, Volume 1: Rule of the Community and Related Documents. Tübingen: JCB Mohr (Paul Siebeck); 1994.

17. Puech E. L’alphabet cryptique A en 4QS^{e} (4Q259). Revue de Qumran. 1998;18:429–435.

18. Jull AJT, Donahue DJ, Broshi M, Tov E. Radiocarbon Dating of Scrolls and Linen Fragments from the Judean Desert. Radiocarbon. 1995;37:11–19. doi:10.1017/s0033822200014740.

19. Puech E. Discoveries in the Judaean Desert: Volume XXV. Qumrân Grotte 4.XVIII. Textes hébreux: 4Q521-4Q528, 4Q576-4Q579. Oxford: Clarendon Press; 1998.

20. Reimer PJ, Austin WE, Bard E, Bayliss A, Blackwell PG, Ramsey CB, et al. The IntCal20 Northern Hemisphere radiocarbon age calibration curve (0–55 cal kBP). Radiocarbon. 2020;62:725–757. doi:10.1017/RDC.2020.41.

21. Ulrich E, Cross FM, Davila JR. Discoveries in the judaean desert: Volume XII. Qumran cave 4.VII. Genesis to Numbers. Oxford: Clarendon Press; 1995.

22. Baumgarten JM. Discoveries in the judaean desert: Volume XVIII. Qumran cave 4.XIII. The Damascus Document (4Q266-273). Oxford: Clarendon Press; 1996.

23. Sirat C. Les manuscrits en caractères hébraïques: Réalités d’hier et histoire d’aujourd’hui. Scrittura e civiltà. 1986;10:239–288.

24. Ulrich E. Discoveries in the judaean desert: Volume XVI. Qumran Cave 4.XI. Psalms to chronicles. Oxford: Clarendon Press; 2000.

25. Cross FM. The ancient library of Qumran. 2nd ed. Garden City, NY: Doubleday; 1961.

26. Popović M. Book Production and Circulation in Ancient Judaea: Evidenced by Writing Quality and Skills in the Dead Sea Scrolls Isaiah and Serekh Manuscripts. In: Williams TB, Keith C, Stuckenbruck L, editors. The Dead Sea Scrolls in Ancient Media Culture. Leiden: Brill; 2023. p. 199–265.

27. Drawnel H. Qumran cave 4: The Aramaic Books of Enoch, 4Q201, 4Q202, 4Q204, 4Q205, 4Q206, 4Q207, 4Q212. Oxford: Oxford University Press; 2019.

28. Broshi M, Eshel E, Fitzmyer J. Discoveries in the judaean desert: Volume XIX. Qumran cave 4.XIV. Parabiblical Texts, part 2. Oxford: Clarendon Press; 1996.

29. Strugnell J, Harrington D, Elgvin T. Discoveries in the judaean desert: Volume XXXIV. Qumran cave 4.XXIV. Sapiential Texts, Part 2: 4QInstruction (Mûsār lĕ Mēvîn): 4Q415 ff. Oxford: Clarendon Press; 1999.

30. Assael Y, Sommerschield T, Shillingford B, Bordbar M, Pavlopoulos J, Chatzipanagiotou M, et al. Restoring and attributing ancient texts using deep neural networks. Nature. 2022;603:280–283. doi:10.1038/s41586-022-04448-z.

31. Bonani G, Ivy S, Wölfli W, Broshi M, Carmi I, Strugnell J. Radiocarbon Dating of Fourteen Dead Sea Scrolls. Radiocarbon. 1992;34:843–849. doi:10.1017/s0033822200064158.
