# Supplementary material for: Dating ancient manuscripts using radiocarbon and AI-based writing style analysis
Source: PLoS One. 2025 Jun 4;20(6):e0323185. doi: 10.1371/journal.pone.0323185 (PMC12136314; doi:10.1371/journal.pone.0323185)
Supplement: S5 Appendix — (PDF) [file pone.0323185.s005.pdf]

S5 Appendix for the article:

Dating ancient manuscripts using radiocarbon and AI-based writing style analysis

Mladen Popović[1*], Maruf A. Dhali[1,2], Lambert Schomaker[2], Johannes van der Plicht[3], Kaare Lund Rasmussen[4], Jacopo La Nasa[5], Ilaria Degano[5], Maria Perla Colombini[5], Eibert Tigchelaar[6],

1 Qumran Institute, University of Groningen, 9712 GK, The Netherlands
2 Artificial Intelligence, Bernoulli Institute, University of Groningen, 9747 AG, The Netherlands
3 Center for Isotope Research, University of Groningen, 9747 AG, The Netherlands
4 Department of Physics, Chemistry, and Pharmacy, University of Southern Denmark, DK 5230, Denmark
5 Department of Chemistry and Industrial Chemistry, University of Pisa, 56126 Pisa PL, Italy
6 Faculty of Theology and Religious Studies, KU Leuven, 3000 Leuven, Belgium

* m.popovic@rug.nl

Data and materials: All data, code, and test film associated with this article are publicly available on Zenodo with the following DOIs:

- Data and prediction plots (v3): https://doi.org/10.5281/zenodo.10998958.
- Code and feature files (v6): https://doi.org/10.5281/zenodo.13319794.
- Film (see details in S7 Appendix: https://doi.org/10.5281/zenodo.8167946.

Please note that this article has 12 appendices in total, from **S1** to **S12**.

S5 Artificial intelligence (AI) in dating the scrolls

In this project, we do use deep learning for image processing (binarization) but have refrained from properly using it for the date prediction. See appendix S6, explaining the objections to the use of deep learning for the date prediction, including an analysis of an experiment we executed, using transfer learning starting with a state-of-the-art foundational deep-learning model.

S5.1 Data preparation

Our first step is to collect and prepare the data for the date prediction model. We collect the images of the manuscripts for each of the ^{14}C samples with valid dates. We have used 24 manuscripts as the primary training set for our date-prediction model (a complete list can be found in appendix S9 in the supplementary materials). The physical 24 radiocarbon-dated manuscripts are visually spread out on many individual fragment images of the IAA's Leon Levy Dead Sea Scrolls Digital Library collection [1]. In addition to this primary training set, we have created different combinations of training data to perform comparative analyses and further check the robustness of the model (see appendix S5.9 for details). We obtained a data set of 75 images from the 24 radiocarbon-dated manuscripts. We use 62 of these images to train our model (Fig S6 shows the size distribution of the training images after the preprocessing steps). The remaining 13 images, chosen deliberately and randomly, are passed as unseen test data to validate the robustness and reliability of the model's performance. We also select a large number of images to perform tests on the date prediction model. Once the images are selected, we start with the preprocessing task, where we use BiNet, the neural network architecture, to extract the characters. The binarization, along with alignment correction and fragment arrangement, provides better-quality images (see appendix S5.1.1). It is extremely important to obtain the highest quality of binarized images. This is because the image quality determines the success of the feature computation and the ultimate date regression model.

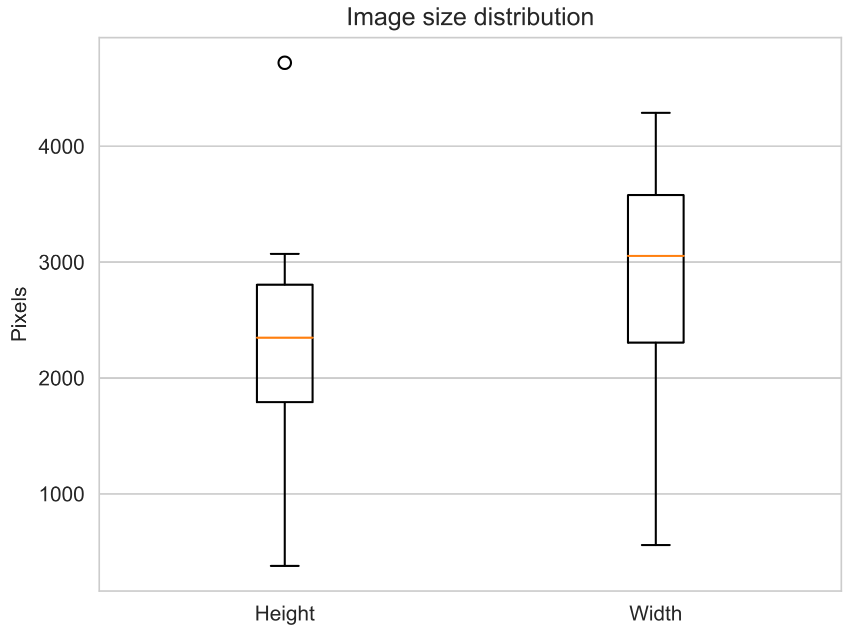

Fig S6. Box-plot showing the height and width spreads of the 62 training images.

S5.1.1 Preprocessing: binarization, alignment and arrangement correction

We start with the multispectral band images for each fragment to use an image fusion technique designed in-house [2] to create pseudo-colour images from a weighted combination of the band images (see Fig S7). The resultant pseudo-colour images offer high contrast and facilitate better separation of ink from backgrounds, a task commonly known as binarization. Both training and test images go through the same preprocessing techniques. It is important to note that although many modern deep-learning methods can be trained directly using the colour/grayscale without binarization, this approach is not suitable for dating the scrolls. Direct end-to-end solutions, i.e., classification or clustering of the training images with testing images, may seem feasible, but there is a risk of obtaining completely inaccurate results. Artificial neural networks, for instance, may make decisions based on superficial correlations with the texture of the parchment, leading to erroneous outcomes. Therefore, isolating only the ink traces (foreground) and excluding any other material features in the images (background) is crucial. BiNet is a deep-learning-based method specially designed to binarize scroll images. Instead of using a simple filtering technique, BiNet uses a neural network architecture for the binarization task and therefore yields better output [2] (see Fig S8).

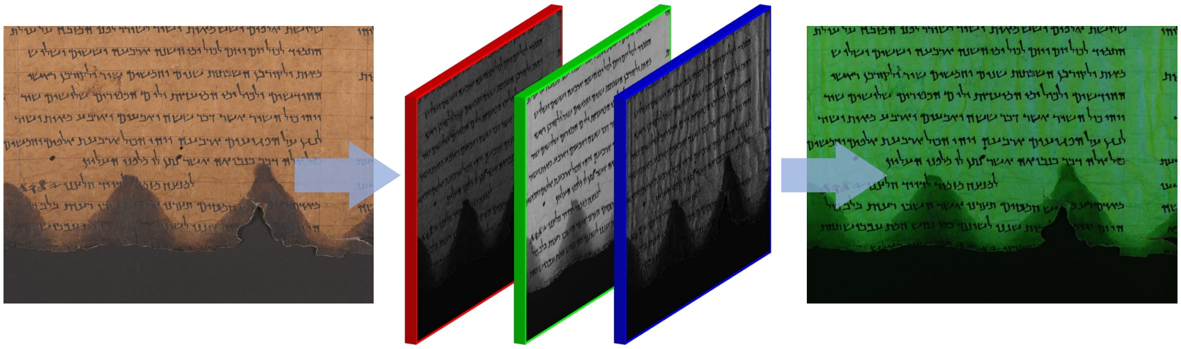

Fig S7. An illustration of creating a pseudo-colour image. The first image from the left is the full spectrum colour image of 11Q5 from plate 974. The next three are the band images (for the formation of three channels using multispectral images) with wavelengths of 595nm, 924nm, and 638nm, respectively. On the right side is the resultant pseudo-colour image (fused image).

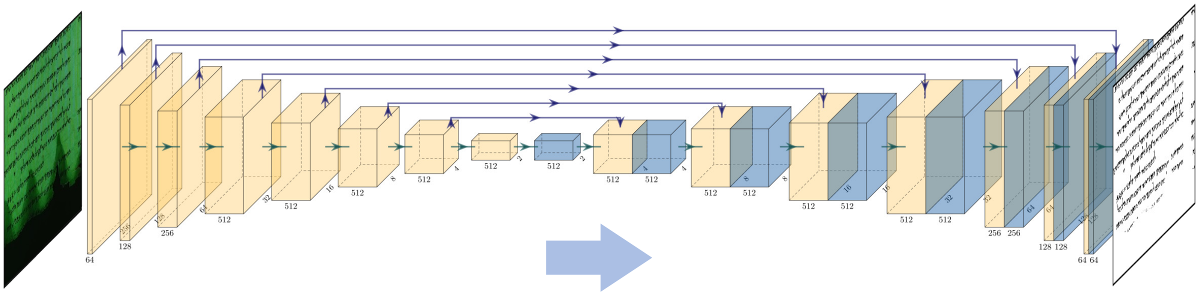

Fig S8. The BiNet architecture shows the encoder (contracting path) at the left half and the decoder (expanding path) at the right half of the image. Each step in the decoder part receives a concatenation with the corresponding feature map from the encoder part through the skip connections. This concatenation circumvents the bottleneck issue at the deepest layers of the encoder and ensures the precise localization of the foreground-background pixels. The example shows a pseudo-colour image of 11Q5 (Plate 974) as input on the left and the output binarized image on the right.

Once the binarization process is complete, additional cleaning of the images is performed. This

cleaning step aims to remove any extra noise or speckles that were not completely removed by the binarization technique. This is a crucial procedure to ensure that features are extracted only from the characters of each image. Subsequently, rotation and alignment correction are also performed. If the images are rotated at some angle to the horizontal axis, it can affect feature calculations that rely on rotation invariance. Therefore, rotation correction is applied to align the text lines horizontally. In some cases, a minor affine transformation and stretching correction are executed in a selective manner. These corrections are specifically intended to align the twisted text lines caused by the degradation of the parchment. In many cases, one manuscript contains multiple fragments. In these cases, we put the fragments together and arrange them into a single image (see Fig S9). The GIMP tool, a free and open-source graphics editor, is used for rotation and arrangement correction [3]. It is important to note that the alignment and arrangement corrections are mostly done with the training images to obtain accurate feature extractions for the style periods represented by those images. However, these corrections are only done for some of the test images due to the limitation of time and resources. Most test images are used directly after binarization, sometimes leading to an unrealistic prediction due to damaged and deformed characters (see Fig S10). If any test images need special attention in the future, extra steps can be performed to obtain a better image for a better prediction from Enoch.

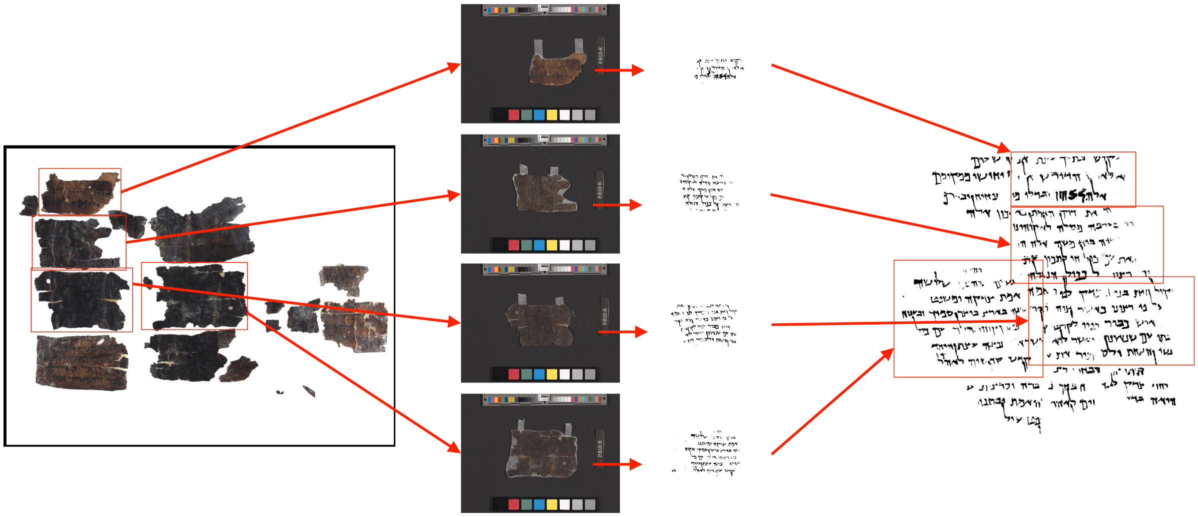

Fig S9. An example of image preparation for 4Q319: a full plate image from IAA is shown on the left side (Plate 810). Then in the middle column, four different fragment images (full spectrum colour images) and their binarized outputs are presented. Finally, further cleaning, alignment correction, and arrangement are performed to produce the final image of 4Q319 on the right.

S5.2 Data augmentation

We have a very limited number of radiocarbon-dated manuscripts from which we derive the training images. During the writing process of any document, writers naturally introduce variability even within the same time period. In order to address both issues of data scarcity and writing variations within a period, we perform data augmentation by introducing acceptable variation to the data. The small random shape perturbations will, on the one hand, ensure the system's robustness and, on the other hand, consider variations of writing styles within a particular period. In machine learning, augmentation is an often-used method to counteract the effects of lack of data and imbalance in sampling [4]. We augment training and testing data by generating synthetic images using random geometric distortions [5].

We perform data augmentation using applying random elastic 'rubber-sheet' transforms. For each pixel (i, j) of the column images, a random displacement vector $(\Delta x, \Delta y)$ is generated. The complete

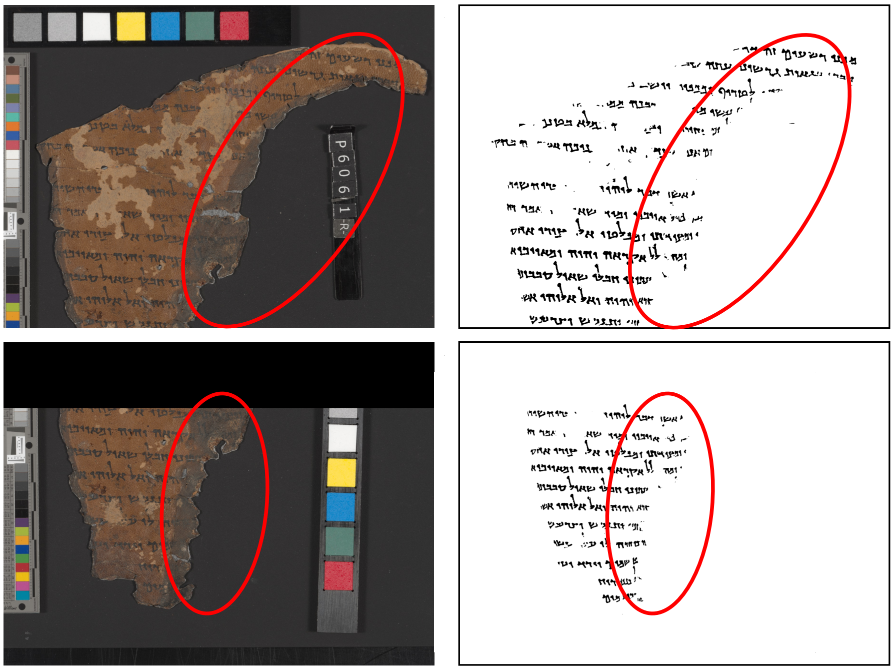

Fig S10. Characters are deformed (marked in red) near the edges of the physical fragments from one of the test manuscripts, 11Q7 (IAA plate 606-1)—these deformities in the binarized images (with slanted or skewed characters) affect the textural and allographic feature calculations.

image's displacement field is smoothed using a Gaussian convolution kernel with a standard deviation σ. We then rescale the field to an average amplitude A. The new morphed image (i', j') is generated using the displacement field and bilinear interpolation:

$$i' = i + \Delta x, j' = j + \Delta y. \quad (1)$$

Two parameters control this morphing process: the smoothing radius σ and the average pixel displacement A. Both parameters are measured in units of pixels. In our experiment, we empirically chose a displacement value of 1.0 and a smoothing radius of 8.0 (see Fig S11).

S5.3 Allographic codebook with neural networks

After binarization with BiNet [2], connected components of ink were fragmented on Y-minima, to prevent large blobs of multi-character components, yielding 'fraglets'. For each fraglet, the contour curve was determined, running over the edge of a connected component in a counter-clockwise manner. Each contour-pixel sequence is 'time' normalized to 200 samples, (cosine, sine) pairs, yielding a feature vector of 400 values. Using the Kohonen [6] self-organizing map neural network, codebooks of 70×70 and 80×80 prototypical contours were computed [7] (see Fig S12). As a proof of concept, 590 manuscripts from the Dead Sea Scrolls collection were manually labeled as 'Hasmonaean' ($Nhas = 307$) or 'Herodian' ($Nher = 283$) by a palaeographer. During *training* on half of the data, each codebook element obtained the counts for its occurrence in 'Hasmonaean' or 'Herodian' manuscripts, respectively. During *testing*, each manuscript is characterized by its relative occurrence of Herodian-like vs. Hasmonaean-like fraglets. Using the 80x80 map and applying a linear SVM [8] on this 2D feature representation, a classification accuracy of 93 ($\pm$ 2.3%) was obtained, computed over 20 random odd/even splits of the 590 manuscripts. Individual accuracy test results: 90.9, 89.2, 93.9, 91.2, 94.2, 90.2, 93.2, 94.2, 91.2, 95.3, 92.9, 96.3, 94.6, 92.2, 95.3, 92.5, 96.6, 88.8, 94.9, 93.2 (%). On the basis of this pilot experiment and earlier work [7], the allographic feature was deemed a usable candidate for the more fine-grained manuscript-dating algorithm using the carbon-dated training samples.

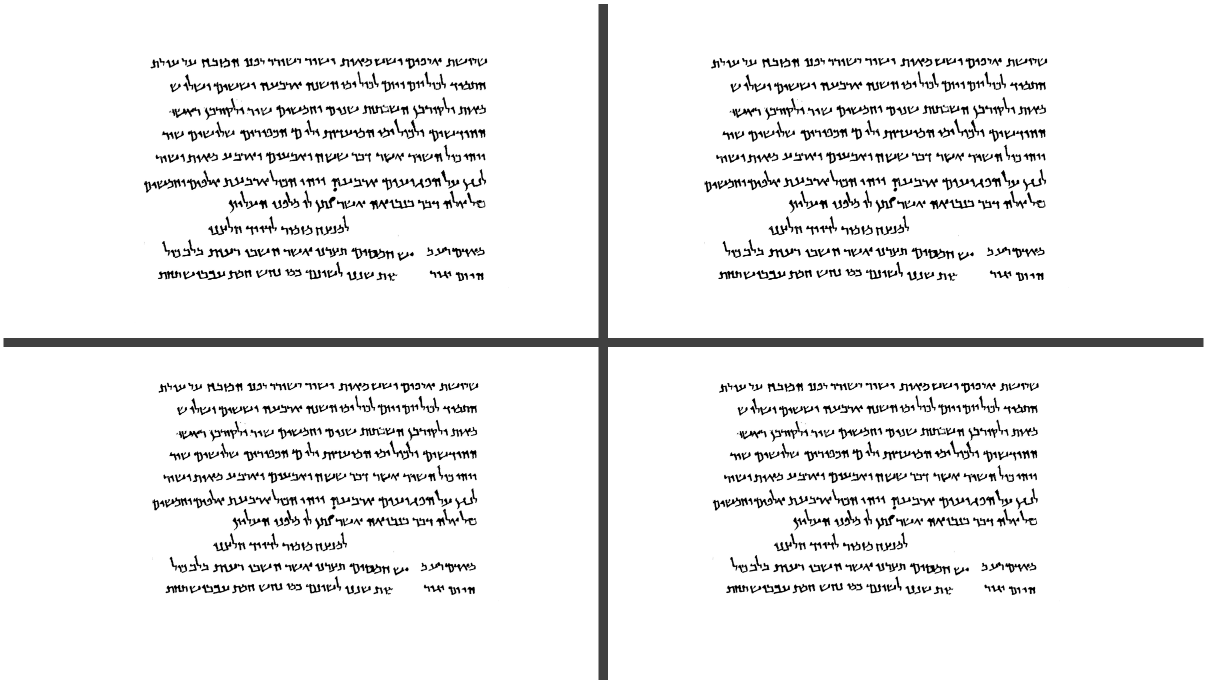

Fig S11. The original binarized image of 11Q5 (Plate 974) on top left and three randomly augmented morphed images. A close inspection of the images shows small geometric distortion introduced to the characters using the elastic-morphing technique.

S5.4 Textural-level features

Similarly, the 'Hinge' feature [7,9] was chosen because of its ability to capture curvature-related differences between different samples of handwriting (see Fig S13). It addresses the occurrence of different degrees of roundness or sharpness of the path described by the edge between ink traces and paper. Its ability to classify between 'Hasmonean' and 'Herodian' styles is less powerful than in the case of allographic fraglets. Using the nearest mean and the Chi-square distance on a 195-dim hinge feature delivers 63.5% accuracy (±2.9%). This dimensionality is still high, and collinearity problems due to feature correlation need to be avoided. Subsequently using PCA, selecting the 15 largest eigenvectors and applying a linear SVM for this binary classification task yields 73.1% accuracy (± 0.24%). Still, on the basis of the complementary nature of the allographic and textural feature methods, it was decided to include the Hinge feature for the manuscript dating problem.

S5.5 Adjoined feature

As shown in [10], the combination of a fraglet codebook and the hinge feature proved to be very effective in writer identification. The assumption in the current study is that different historical style periods are revealed by the statistical characteristics both of allographic shape fragments and of angular distributions. Consider, for instance, a manuscript with predominantly vertical and horizontal strokes ('formal') and a manuscript written in a more informal ('cursive') style, both containing their characteristic shape elements in individual characters. The use of the two feature methods together will capture the underlying shape differences. The feature combination is realized by an adjoining of the two feature vectors: the arrays of feature values are combined in a single array containing the combined descriptor. Adjoined features are the weighted combination of both Hinge and Fraglet. The adjoining results in a feature vector of 5365 dimensions, preserving the handwriting style description from both feature levels.

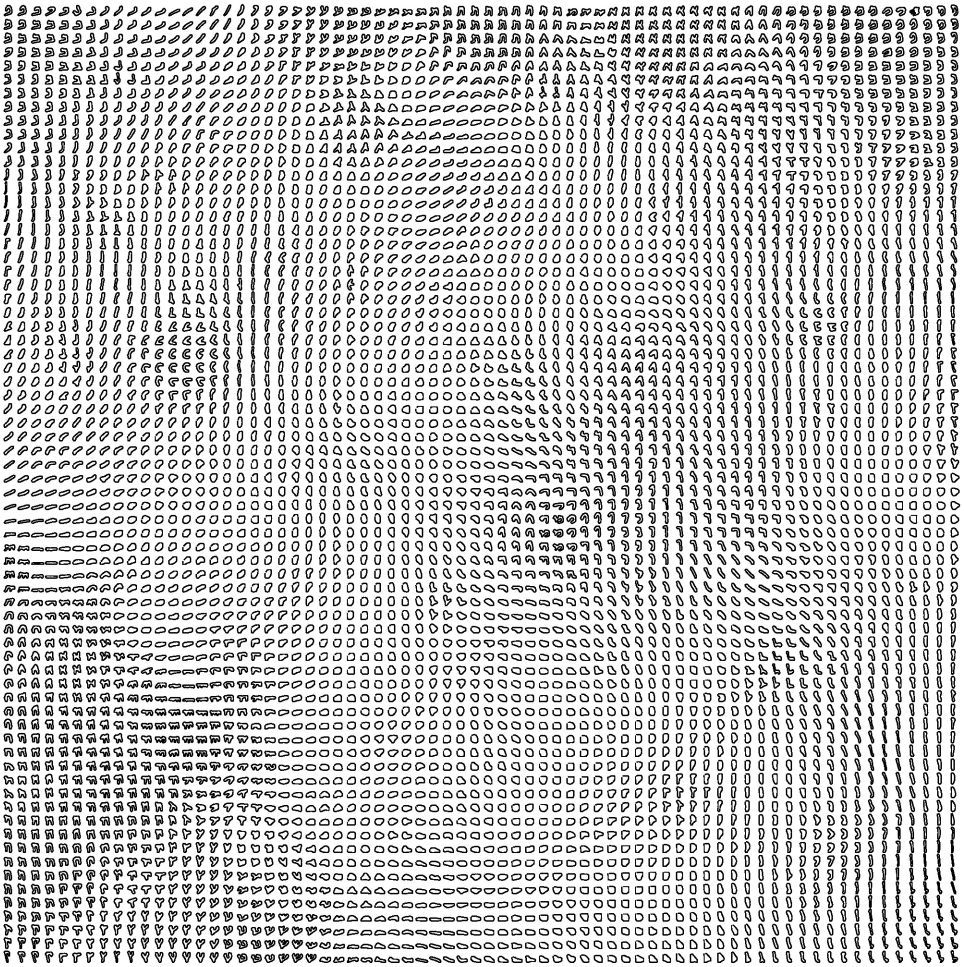

Fig S12. A visualization of 70x70 Kohonen map of fragmented connected components (200 x,y points per contour centroid) from the Dead Sea Scrolls collection. Image adapted from [7].

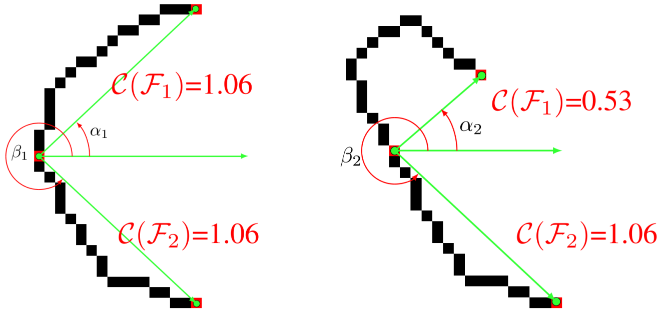

Fig S13. Hinge kernel; the angles and leg-lengths for two different character shapes. Image adapted from [7].

S5.6 Date-prediction model

We employ our date-prediction model once the features are calculated from the images. Given, for each manuscript, a style feature vector, we now address the transformation of this representation into an OxCal-type curve, i.e., a vector containing the estimated date probabilities for the sample. Because of

the small size of the data set, high-parametric models such as period-specific temporal codebooks [11] cannot be used here. We use conditional modeling using Bayesian Ridge regression [12] that applies Bayesian inference to estimate the model parameters for date prediction. First, a prior distribution is placed on the model parameters, which expresses known constraints on the values of the parameters. The prior distribution is then updated with the observed data using Bayes' rule to obtain the parameters' posterior distribution and predicted dates.

We propose the Bayesian approach due to the nature of our target output data: the ^{14}C data are not a single point on the timeline but are given as a distribution of probable dates within sigma (σ) ranges. Hence, the probabilistic approach allows the use of all available information while remaining explainable. Furthermore, we can observe a full posterior distribution, which is used to assess the uncertainty of the estimated dates. Finally, the Bayesian approach also allows the model to indicate error margins for predictions on unseen data.

S5.6.1 Unmodelled values from OxCal

The input of the date prediction model is the feature vectors of the training images, along with their probability distribution from radiocarbon dating as labels. We obtain the probability distribution as unmodelled raw values of 5-year resolution after the radiocarbon calibration was performed using OxCal v4.4.2 [13,14]. We created a new project code for the 26 sample manuscripts using the ^{14}C age (BP) and sigma (BP) values (see Table S1 for the BP values. For the code, please check the Zenodo repository (https://doi.org/10.5281/zenodo.13319794)). Using the measured BP values, the code (entirely reproducible) uses the simple format:

```
Plot()
  {
  R_Date("Q-number", age(BP), sigma(BP));
   };
```

OxCal generates the clean unmodelled (BCE/CE) probability values in a `.csv` file once the code is run. We obtain these values for each individual sample from OxCal options: *View ≫ Raw output.* Please note that we do not specify any resolution in our code. Hence, our raw data are in the default resolution of 5 years, which is the same as the resolution of the IntCal curves, so no interpolation or binning is needed. It is possible to set the resolution to less than 5. Then, the curve will be interpolated by a cubic (or linear if that option is set) function by OxCal (as explained in appendix S2.4).

S5.6.2 Calibrated dates from 2-sigma ranges

Having performed the radiocarbon dating in its entirety (appendix S2) and then the palaeographic evaluation of the calibrated dates (appendix S4), as was also done in [15,16], we use the calibrated dates from the 2σ range for firmer grounding of our date-prediction model. In the case of bimodal evidence, palaeography determines that in most cases the younger 2σ peak should be used for analysis. But palaeography cannot be characterised as a specific quantitative prior (an expected date with mean and standard deviation) in the timeline. The issue is that we cannot assume a single point-spread density (Gaussian) along the time axis. Palaeography, in this sense, does not deliver a point-wise prior. What palaeography can deliver, is the identification of a point on the timeline that represents the historical impossibility of a range of dates on the left or right. This knowledge is based on intersubjective expert knowledge (appendix S4.2). Therefore, the use of expert knowledge in our case is not based on the usual Bayesian-plus-Gaussian method but on a more direct use of existing palaeographic domain knowledge, which allows splitting the 2σ calibrated date range into a 'left-half' vs. 'right-half' time region of interest. Although not Gaussian (there exists no list of dated objects of which the mean and standard deviation can be used for Bayesian/Gaussian stratigraphy), this procedure is still probabilistic in the sense that it entails the multiplication of the OxCal output distribution with a step function.

Specifically, the palaeographic knowledge (appendix S4.2) allows to make a binary split in the OxCal distribution of bimodal 2σ ranges using the Heaviside function with the position of the step being

placed at an innocuous low-probability point on the curve, where the probability has a plateau around zero. Applying a Heaviside multiplicative bias on the empirical density function is a valid Bayesian approach to perform peak selection. Another, e.g., smooth logistic variant of the step function or a cumulative Gaussian could have been used. In either case this would require the specification of a steepness parameter, the value of which is unknown. Similarly, from the palaeographic constraints, the standard deviation which would be needed under a Gaussian, i.e., point-localized density assumption in the Bayesian reasoning process, is not known. Note that apart from the Gaussian, many other distribution functions exist, e.g., Poisson, Weibull, Gamma, etc., for other applications in Bayesian reasoning. Such distribution functions could be used, *if* there are reasons to assume them. We do not have any reason for a detailed distribution choice and can only choose to disregard 'impossible time regions'. In our case, for most bimodal 2σ calibrated ranges we assume that the palaeographic evaluation that a left-most (or right-most) region is impossible is correct (see appendix S4.2), leading to a collapse of the probabilities in that range. The shape of the remaining distribution reflects the likelihood of dates.

Thus, the procedure we use is as follows:

1. We perform the radiocarbon dating in its entirety, with the calibrated dates having been generated using OxCal data with only ^{14}C (BP) and σ (BP), see appendix S2.

2. We use the calibrated dates from the 2σ range for firmer grounding of our date-prediction model. Only in the case of bimodal evidence in these 2σ ranges do we apply the Heaviside-function at a near-zero probability point on the curve to reject older peaks and accept younger/'right-hand' peaks as a possible solution, on the basis of expert palaeographic knowledge (appendix S4).

3. From OxCal, we obtain the raw data of the probability densities of the 2σ ranges, which are used as input in our date-prediction model.

4. We work with the inclusion and exclusion of so-called minor or smaller probability peaks, which in 10 out of 12 instances have a probability of less than 3.5%; in the remaining two cases, it is 5.2% and 9.4% (see Table S8). The inclusion or exclusion of these peaks has minimal and insignificant consequences for the interpretation of the results (see appendix S5.7).

5. Because applying the Heaviside function for bimodal evidence leaves less than 95.4% of the entire 2-σ probability for each sample, we normalise the accepted 2σ calibrated probabilities. The output probability predictions of the dating model are also balanced and normalised using both weights and data augmentation (see appendix S5.7).

6. Within the accepted part of the 95.4% confidence range, the points in the probability distribution curve as calculated by OxCal are used as target values in the training of Enoch. The output distribution delivered by the Enoch model is a mixture of Gaussians approximating the shape of the OxCal curve. From those outputs, we select the 1σ range for a clear, narrow visualization of the predicted date ranges (see Fig 1 in the main article). This choice is independent of the original selection of OxCal calibration ranges because the two methods are fundamentally different.

We emphasise that we do not perform modelling within the OxCal programme as is commonly used in radiocarbon dating practice. This does not compromise the transparency and reproducibility of our procedure. Using the ^{14}C dates in BP and their measurement uncertainties (σ), all plots can be reproduced using OxCal. Our reasoning for rejecting part of the bimodal data (see appendix S4) and the exact 2σ probabilities we use (see Table S5.6.2) are provided. Yet, other researchers can also use all our ^{14}C data instead of following our reasoning for accepting part of the bimodal data, and justify their reasoning.

Table S7. Unmodelled radiocarbon calibrated dates for 2σ ranges. Please note that the 2σ values are the same as Table S1 in Appendix S2. However, in this table, the highlighted date ranges indicate each sample's accepted 2σ intervals for the Enoch model.

Q-number	2σ range		2σ range		2σ range		2σ range	
4Q504	-355	-285	-230	-150				
4Q52	-410	-355	-285	-230				
4Q176	-355	-300	-210	-100	-70	-60		
4Q114	-355	-285	-230	-160				
5_6Hev1b	10	205						
4Q161	-90	-80	-55	30	45	60		
4Q70	-375	-345	-320	-200				
4Q47	-355	-290	-210	-100				
4Q23	-355	-285	-230	-220	-210	-95	-75	-55
4Q255_4Q433a	-170	-50						
11Q5	-35	-15	5	120				
4Q3	-340	-325	-200	-50				
4Q27	-340	-330	-200	-50				
Mas1k	-50	65						
4Q206	-360	-280	-235	-145	-135	-120		
4Q30	-360	-275	-260	-245	-235	-165		
4Q201_4Q338	-165	-40	-10	-1				
4Q259	-350	-310	-210	-100	-70	-55		
4Q416	-345	-320	-205	-90	-80	-50		
4Q2	-155	-130	-125	10				
4Q375	-345	-320	-205	-50				
Xhev_Se2	-45	75						
4Q541	-355	-300	-210	-95	-75	-55		
4Q521	-355	-285	-230	-100				
4Q267	-355	-290	-210	-95	-70	-55		
Mur19	-45	85	95	110				

In the following subsections, we present the mathematical derivation of the Bayesian regression from simple linear regression as used in Enoch, our date prediction model.

S5.6.3 Linear regression

Given a set of training data $\{(\mathbf{x_n}, t_n)\}_{n=1}^{N}$ comprising N observations of dimensionality M, where $\mathbf{x}_n \in \mathbb{R}^M$, $t_n \in \mathbb{R}$, the goal in a regression model is to find a linear mapping $f : \mathbb{R}^M \to \mathbb{R}$ which approximates t_n given $\mathbf{x_n}$ as close as possible. Furthermore, the mapping should generalize to values outside the training data. From a probabilistic perspective, the aim is to model the *predictive distribution* $p(t_n \mid \mathbf{x}_n)$. In a linear regression model, the assumption is that the target variable t is given by a deterministic function $f(\mathbf{x}_n, \mathbf{w})$ with added Gaussian noise, such that:

$$t_n = f(\mathbf{x}_n, \mathbf{w}) + \epsilon \tag{2}$$

where ϵ is a Gaussian random variable with mean 0 and inverse variance parameter β, also called the *precision*. In a linear model where $f(\mathbf{x}_n, \mathbf{w}) = \mathbf{w}^T\mathbf{x}$, the predictive distribution takes the form

$$p(t_n \mid \mathbf{x}_n) = \mathcal{N}(\mathbf{w}^T\mathbf{x}_n, \beta^{-1}). \tag{3}$$

Even though the predictive distribution is indirectly used to optimize the parameters of the linear regression model, we do not explicitly model this distribution. We will see in the next section that in the Bayesian interpretation of linear regression, we instead stay within a probabilistic framework and model the full predictive distribution, leading to several advantages over the standard linear regression model.

We first turn to parameter estimation for a linear model. This means estimating a value for the weight vector $\mathbf{w}$ that fits the data well. Most commonly, the *least-squares* criterion is used to estimate the weight vector $\mathbf{w}$:

$$\mathbf{w}^* = \arg\min_{\mathbf{w}^*} \sum_{n=1}^{N} (t_n - \mathbf{w}^T\mathbf{x}_n)^2 \tag{4}$$

This can be justified using maximum likelihood estimation if we assume that the training data is independent and identically distributed (i.i.d.). This works as follows. Let $\mathbf{X} = (\mathbf{x}_1, \ldots, \mathbf{x}_N)^T$ and $\mathbf{t} = (t_1, \ldots, t_N)^T$. The log-likelihood of the training data can then be written as

$$\begin{aligned}\ln p(\mathbf{t} \mid \mathbf{X}, \mathbf{w}, \beta) &= \ln \prod_{n=1}^{N} p(t_n \mid \mathbf{x}_n, \mathbf{w}, \beta) \\ &= \ln \prod_{n=1}^{N} \mathcal{N}(t_n \mid \mathbf{w}^T \mathbf{x}_n, \beta^{-1}) \\ &= \sum_{n=1}^{N} \ln \mathcal{N}(t_n \mid \mathbf{w}^T \mathbf{x}_n, \beta^{-1}) \\ &= \sum_{n=1}^{N} \ln\{(2\pi)^{-1/2} \beta^{1/2} \exp(-\frac{\beta}{2}(t_n - \mathbf{w}^T \mathbf{x}_n)^2)\} \\ &= \frac{N}{2} \ln \beta - \frac{N}{2} \ln 2\pi - \beta E_D(\mathbf{w}), \end{aligned} \tag{5}$$

where we make use of (3). The $E_D(\mathbf{w})$ term represents a sum-of-squares function, defined as

$$E_D(\mathbf{w}) = \frac{1}{2} \sum_{n=1}^{N} (t_n - \mathbf{w}^T \mathbf{x}_n)^2. \tag{6}$$

Considering that maximizing the likelihood function with respect to $\mathbf{w}$ only depends on $E_D(\mathbf{w})$, expression (5) can be maximized by maximizing $-E_D(\mathbf{w})$, or equivalently, minimizing $E_D(\mathbf{w})$. This corresponds to the least-squares objective shown in (4).

S5.6.4 Ridge regression

We now turn to the ridge regression model, an extension of the linear regression model with more desirable properties, such as mitigating over-fitting. Concretely, we add a prior distribution over the weights $\mathbf{w}$, leading to the following log-likelihood function:

$$\ln p(\mathbf{t} \mid \mathbf{X}, \mathbf{w}, \beta) + \ln p(\mathbf{w} \mid \alpha). \tag{7}$$

The prior distribution over the weights can be interpreted with the Bayes rule, showing the relationship to a posterior distribution over $\mathbf{w}$:

$$p(\mathbf{w} \mid \mathbf{t}) \propto p(\mathbf{t} \mid \mathbf{w}) p(\mathbf{w}), \tag{8}$$

where we omit the $\mathbf{X}$, α, and β terms to keep the notation uncluttered. In other words, maximizing (7) corresponds to maximizing a posterior distribution over $\mathbf{w}$. The question now arises what is a suitable form of the prior distribution $p(\mathbf{w})$? To ensure that $p(\mathbf{w} \mid \mathbf{t})$ has the same functional form as $p(\mathbf{t} \mid \mathbf{w})$, we choose $p(\mathbf{w})$ to be a conjugate prior of $p(\mathbf{t} \mid \mathbf{w})$, namely a multivariate isotropic Gaussian distribution, taken to be zero-centered with precision parameter α. The log-likelihood then becomes

$$\ln p(\mathbf{t} \mid \mathbf{X}, \mathbf{w}, \beta) + \ln p(\mathbf{w} \mid \alpha) =$$

$$\sum_{n=1}^{N} \{\ln \mathcal{N}(t_n \mid \mathbf{w}^T \mathbf{x}_n, \beta^{-1})\} + \ln \mathcal{N}(\mathbf{w} \mid 0, \alpha^{-1}\mathbf{I}) \tag{9}$$

$$= \tag{10}$$

$$\frac{N}{2} \ln \beta - \frac{N}{2} \ln 2\pi - \beta E_D(\mathbf{w}) + \frac{M}{2} \ln \alpha - \frac{M}{2} \ln 2\pi - \alpha E_W(\mathbf{w}), \tag{11}$$

where M denotes the number of dimensions of the weight parameter $\mathbf{w}$ and I denotes the identity matrix. Note that the first three summands of (11) correspond to (5). The $E_W(\mathbf{w})$ term represent a regularization term, defined by

$$E_W(\mathbf{w}) = \frac{1}{2}\mathbf{w}^T\mathbf{w}. \tag{12}$$

By removing terms from (11) that do not depend on $\mathbf{w}$, we end up minimizing the sum of two terms, $E_D(\mathbf{w})$ and $E_W(\mathbf{w})$, denoting the data-dependent error and the regularization error, respectively. The relative importance of both terms is controlled by the α and β hyperparameters. Equivalently, we minimize:

$$\beta E_D(\mathbf{w}) + \alpha E_W(\mathbf{w}). \tag{13}$$

If we combine α and β into one hyperparameter $\lambda = \alpha/\beta$, we can equivalently write

$$E_D(\mathbf{w}) + \lambda E_W(\mathbf{w}), \tag{14}$$

Which corresponds to

$$\frac{1}{2}\sum_{n=1}^{N}(\mathbf{w}^T\mathbf{x}_n - t_n)^2 + \frac{\lambda}{2}\mathbf{w}^T\mathbf{w}, \tag{15}$$

Which forms the ridge-regression objective function. The λ hyperparameter can control the degree of *parameter shrinkage* [17], whereby the weight parameters are shrunk by imposing a penalty on their size. This brings the additional task of setting λ, generally done using cross-validation. By setting the gradient of (15) with respect to $\mathbf{w}$ to 0 and solving for $\mathbf{w}$, the approximate solution can be expressed in closed form using the standard equations:

$$\mathbf{w} = (\mathbf{X}^T\mathbf{X} + \lambda\mathbf{I})^{-1}\mathbf{X}^T\mathbf{t}. \tag{16}$$

S5.6.5 Bayesian regression

We now turn to a Bayesian treatment of the ridge regression model discussed in the previous subsection. First, consider the relationship we established between the posterior over $\mathbf{w}$ and the product of the likelihood and prior, as shown in (8). This is the point at which the ridge and Bayesian regression models diverge in their approach. For the ridge regression model, a point estimate for the weight vector $\mathbf{w}$ is obtained by using maximum a posteriori estimation (MAP), which involves maximizing the right-hand side of (8). We now discuss the alternative, fully Bayesian treatment, which explicitly models the posterior distribution on the left-hand side of (8).

Recall that we defined the prior distribution $p(\mathbf{w})$ as a conjugate prior to the likelihood function, leading to a multivariate Gaussian distribution. The result is that the posterior $p(\mathbf{w} \mid \mathbf{t}, \mathbf{X})$ also will have a Gaussian distribution. We can thus rewrite (8) to:

$$\mathcal{N}(\mathbf{m}_N, \mathbf{S}_N) \propto \mathcal{N}(\mathbf{X}\mathbf{w}, \beta^{-1}\mathbf{I})\mathcal{N}(0, \alpha^{-1}\mathbf{I}), \tag{17}$$

where the posterior is a Gaussian with mean $\mathbf{m}_N$ and covariance $\mathbf{S}_N$. We can use the Bayes theorem for Gaussian random variables to find $\mathbf{m}_N$ and $\mathbf{S}_N$. From this, it follows:

$$\mathbf{m}_N = \beta\mathbf{S}_N\mathbf{X}^T\mathbf{t} \tag{18}$$

$$\mathbf{S}_N^{-1} = \beta\mathbf{X}^T\mathbf{X} + \alpha\mathbf{I}. \tag{19}$$

It is worth noting the correspondence between the point estimate of $\mathbf{w}$ obtained in the ridge regression solution (16) and the mean of the posterior $\mathbf{m}_N$. If we fully write out $\mathbf{m}_N$, we see that

$$
\begin{aligned}
\mathbf{m}_N &= \beta(\beta\mathbf{X}^T\mathbf{X} + \alpha\mathbf{I})^{-1}\mathbf{X}^T\mathbf{t} \\
&= \beta(\beta(\mathbf{X}^T\mathbf{X} + \lambda\mathbf{I}))^{-1}\mathbf{X}^T\mathbf{t} \\
&= \beta(\beta^{-1}(\mathbf{X}^T\mathbf{X} + \lambda\mathbf{I})^{-1})\mathbf{X}^T\mathbf{t} \\
&= (\mathbf{X}^T\mathbf{X} + \lambda\mathbf{I})^{-1}\mathbf{X}^T\mathbf{t},
\end{aligned}
$$

which corresponds to (16). This means that the mode of the posterior distribution corresponds to the ridge regression solution. However, we use the full posterior distribution over $\mathbf{w}$ in the Bayesian regression approach rather than taking the mean $\mathbf{m}_N$ as a point estimate. This works as follows. We first note that once we obtained the posterior distribution over $\mathbf{w}$, the predictive distribution informed by the training data can now be written as

$$p(t \mid \mathbf{x}, \mathbf{t}, \mathbf{X}) = \int p(t \mid \mathbf{x}, \mathbf{w}) p(\mathbf{w} \mid \mathbf{t}, \mathbf{X})\, d\mathbf{w} \tag{20}$$

for an input $\mathbf{x}$, where we once again omit the α and β terms for readability. Noting that (20) is a marginal distribution and a convolution of two Gaussians, we can once again make use of Bayes theorem for Gaussian variables, resulting in the predictive distribution

$$p(t \mid \mathbf{x}, \mathbf{t}, \mathbf{X}, \alpha, \beta) = \mathcal{N}(t \mid \mathbf{m}_N^T\mathbf{x}, \beta^{-1} + \mathbf{x}^T\mathbf{S}_N\mathbf{x}). \tag{21}$$

The mean of this distribution is simply the mean of the posterior distribution multiplied by the input vector. As can be seen from the variance parameter of this equation, the predictive variance associated with an input $\mathbf{x}$ consists of a sum of two terms, which can be understood as follows. The first term expresses variance due to the noise in the training data. The second term describes the uncertainty associated with $\mathbf{w}$, which varies according to the input $\mathbf{x}$.

Given this predictive distribution, we can make predictions for new input values by calculating the conditional expectation,

$$\mathbb{E}[t \mid \mathbf{x}, \mathbf{t}, \mathbf{X}] = \int t\, p(t \mid \mathbf{x}, \mathbf{t}, \mathbf{X})\, dt. \tag{22}$$

An alternative is to directly take the mean $\mathbf{m}_N$ of the posterior as an estimate for $\mathbf{w}$, which is used in some implementations of the Bayesian regression model [18].

S5.6.6 Hyperparameter selection

In a Bayesian framework, defining a prior distribution over one or both hyperparameters, also known as a *hyperprior*, can be used in finding hyperparameters using cross-validation. We can then marginalize all the parameters, which leads to a predictive distribution of the form.

$$p(t \mid \mathbf{x}, \mathbf{t}, \mathbf{X}) = \iiint (t \mid \mathbf{x}, \mathbf{w}) p(\mathbf{w} \mid \mathbf{t}, \mathbf{X}, \alpha, \beta) p(\alpha, \beta \mid \mathbf{t}, \mathbf{X})\, d\mathbf{w}\, d\alpha\, d\beta. \tag{23}$$

Unfortunately, this expression is analytically intractable. Nevertheless, a framework for calculating an approximation, named *evidence approximation* [19], can compute estimates for α and β. This framework is also referred to as *type II maximum likelihood*, which involves maximizing the *marginal likelihood function* $p(\mathbf{t} \mid \alpha, \beta)$, where $\mathbf{w}$ has been integrated out. We will not go into depth into the evidence approximation framework. For more extensive treatment, the reader is referred to the statistical book [19]. It should be noted that this approach, where we include the hyperparameters as part of the training process by regarding them as random variables, does not necessarily lead to better estimates than those obtained with cross-validation. Nevertheless, automatically finding hyperparameters as part of the training process can be helpful in certain situations, for example, if cross-validation is not feasible.

The output of the date prediction model is a probability estimate for each 10-year bin in our timeline, along with error margins to estimate uncertainty. Within the Bayesian regression, we apply parameter

constraints to restrict the uncertainties to non-negative values as they do not impact the probability estimation of our model, and the final results and interpretation. However, future research can explore the feasibility of an asymmetric error estimation above the x-axis. The choice of the 10-year bin is made empirically, and we keep the option of changing the bins to 5 or 15 for either thinner or thicker plots.

S5.7 Data balancing

In addition to our original training data and the date prediction model described in the previous sections, we also employ two types of data balancing techniques to help reduce the time-axis bias in the training data. As can be seen in Figs S14 and S15, the training data is biased in the sense that there are many more high probabilities in the -200 to the -150 region. This creates much higher priors in that region. However, this bias is caused only by the samples that were chosen to be radiocarbon-dated and are not representative of the actual prior probability for the whole Dead Sea Scrolls collection. In order to make the predictions less dependent on the priors within the training data, two data-balancing strategies were implemented.

The first method concerns *balancing using weights*, where the output probabilities from the model are dampened or boosted based on the weights provided by the overall accumulated distribution seen in Figs S14 and S15.

The other data-balancing implementation was through *augmentation*, where underrepresented training data was compensatorily oversampled based on the overall accumulated distribution. The technical details for both implementations will be described in the following subsections.

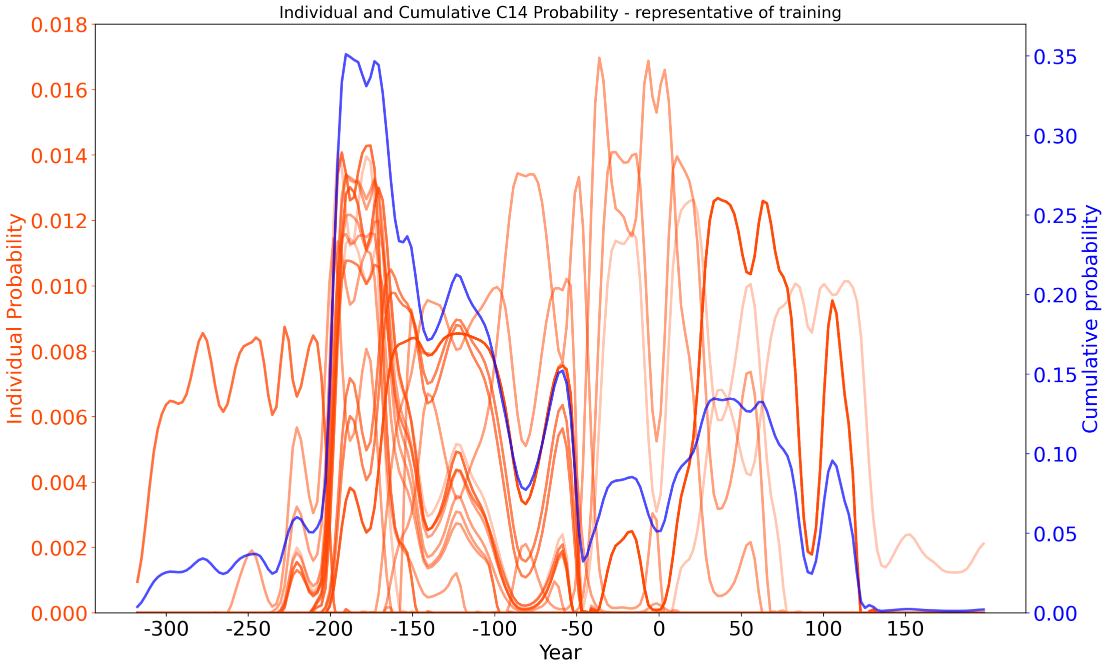

Fig S14. Distributions in the training data (orange) and total accumulated distribution of the C14 training data (blue), **including** all minor peaks of the (accepted) 2-sigma ranges.

Please note that in addition to cross-validation and leave-one-out statistical tests (see appendix S5.9.1), we also check the sensitivity of the model with the inclusion and exclusion of minor peaks on the (accepted) 2σ ranges. Table S8 gives the minor peak ranges and percentages for the 13 manuscripts for which the minor peaks can be included or excluded. Figs S14 and S15 already show minimum changes over the

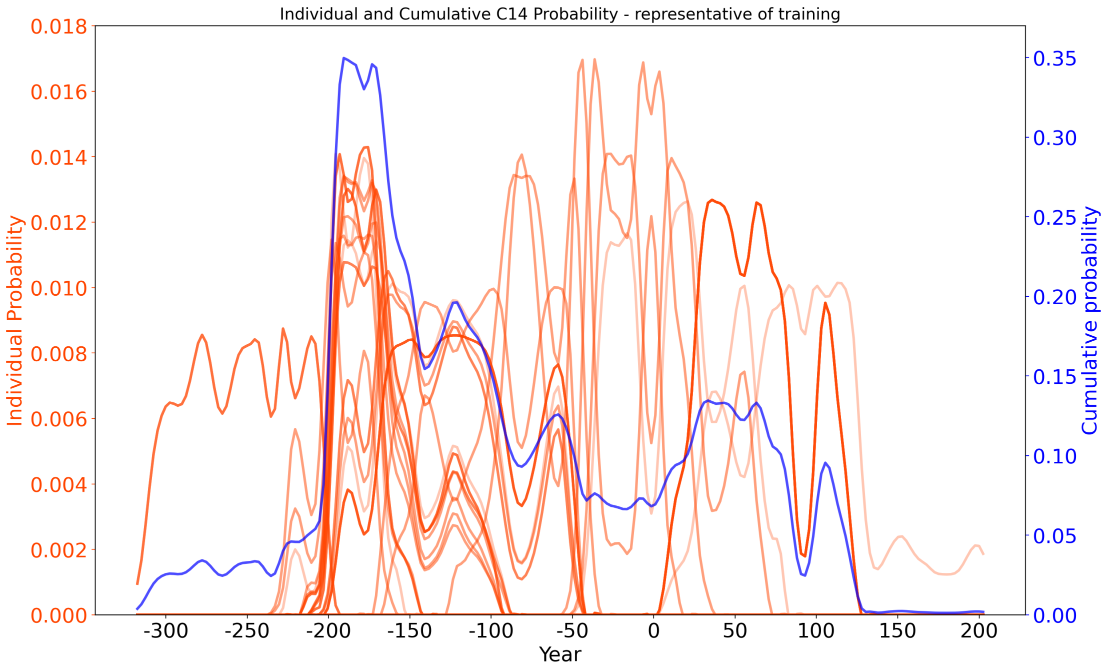

Fig S15. Distributions in the training data (orange) and total accumulated distribution of the C14 training data (blue), **excluding** minor peaks.

overall probability distribution. This can be better visualized from Fig S16. The Euclidean distance calculated over the whole range sampled by five years is 0.104 between the two (accepted) ranges (with and without minor peaks). The chi-square and Bhattacharyya distances are 0.124 and 0.044, respectively, showing no significant changes in the overall probability distribution. The predicted test results also remain unchanged. It is important to note, that incorporating the minor peaks did not lead to horizontal time shifts in existing high-probability peaks, at all (see Fig S16).

S5.7.1 Balance using weights

Given probability p_i where i is a given year, threshold T, (binned) accumulated C14 probability cum_c14, maximum accumulated C14 probability $M = \max(cum\_c14)$, and the number of summations that generated the accumulated probability in a bin $n_{cum\_c14_i}$. The weighted probability of each bin w_{p_i} is calculated as:

$$w_{p_i} = \begin{cases} \frac{p_i}{cum\_c14_i} & \text{if } (p_i > T \cdot M) \text{ and } (n_{cum\_c14_i} > 2) \\ p_i & \text{otherwise} \end{cases} \tag{24}$$

The weighted probabilities are then normalized to ensure that the scale of the weighted predictions is consistent with the original predictions. The process is described below.

Given the global maximum probability values in the original predictions, max_p, and in the weighted predictions, $max\_weighted\_p$, the normalization process for each probability value w_{p_i} in the weighted predictions is described as follows:

Calculate the normalized probability $w_{p\_norm_i}$:

$$w_{p\_norm_i} = \frac{w_{p_i}}{max\_weighted\_p} \cdot max\_p \tag{25}$$

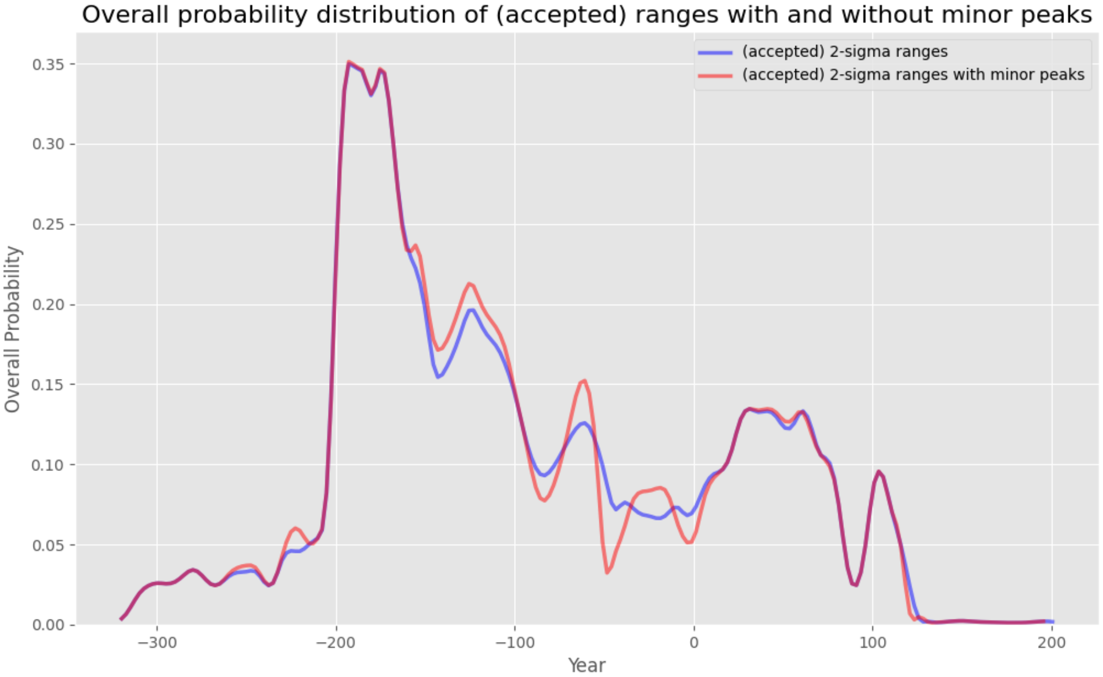

Fig S16. Comparison between overall probability distributions of (accepted) ranges with the inclusion and exclusion of minor peaks, as mentioned in the procedure in appendix S5.6.2.

Table S8. The minor peak ranges and percentages for the 12 manuscripts for which the minor peaks can be included or excluded. Please note that 4Q161 and 4Q23 have minor peaks on both sides of the (accepted) 2σ ranges. The OxCal figures in Appendix S3 clearly illustrate these minor peaks. See also appendix S5.7.

Q-number	Minor peak range		Percentage (%)
**4Q176**	-70	-60	0.7
**4Q161**	-90	-80	1.7
	45	60	1.7
**4Q23**	-230	-220	0.8
	-75	-55	2.1
**11Q5**	-35	-15	3.3
**4Q206**	-135	-120	1.1
**4Q30**	-260	-245	1.4
**4Q201_4Q338**	-10	-1	1.9
**4Q259**	-70	-55	1.4
**4Q416**	-80	-50	9.4
**4Q2**	-155	-130	5.2
**4Q541**	-75	-55	2.7
**4Q267**	-70	-55	1.6

S5.8 Balance using augmentation

For augmentation, five scrolls were chosen to be duplicated in the training data in order to boost the underrepresented prior probabilities within the training data. Table S9 details the scrolls and number

of fragments after the augmentation was applied, and Fig S17 shows the effect this had on the overall accumulated probabilities.

Table S9. Table detailing number of duplications after augmentation procedure

Scroll	Number of fragments originally in the training data	Number of fragments after augmentation
4Q2	2	12
4Q161	2	12
5_6Hev1b	1	6
11Q5	9	54
Mas1k	2	12
XHev_Se2	1	6

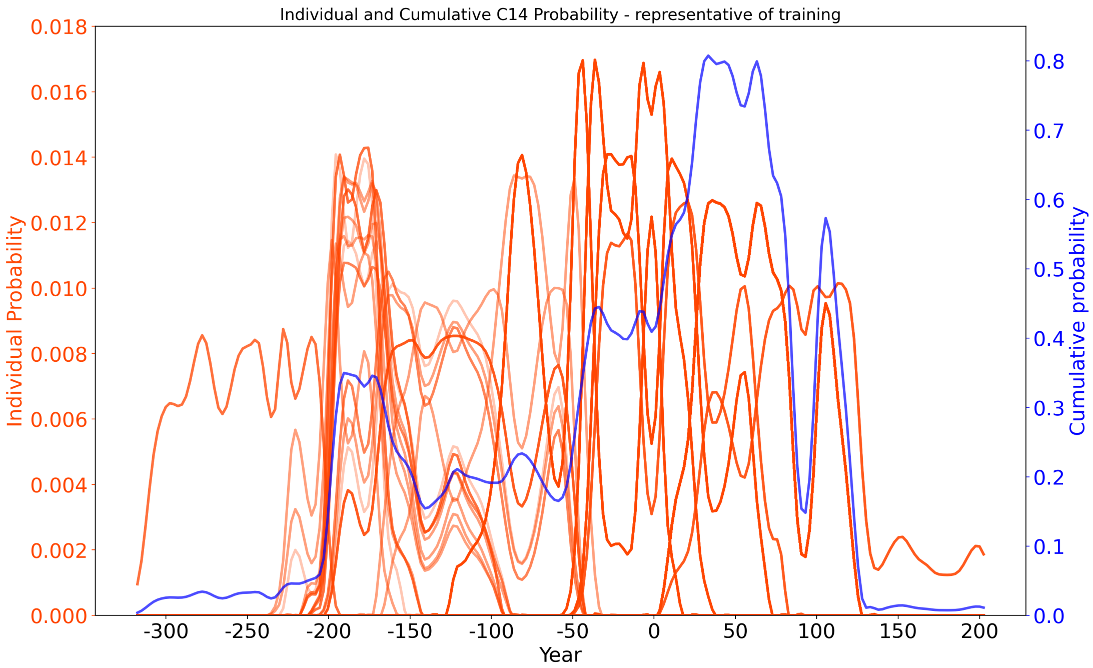

Fig S17. Distributions in the training data (orange) and overall accumulated distribution (blue) of the C14 training data **after augmentation**.

S5.9 Training options

For training, we create three main pools of data:

- ^{14}C dated manuscripts
- ^{14}C dated manuscripts with the addition of the old ^{14}C from the 1990s
- ^{14}C dated manuscripts with augmentation

Within each of these training datasets, there are different possible subsets of the training data that are usable (all the following subsets include the ^{14}C dated manuscripts):

- 4Q52 can be included or excluded
- Internally dated scrolls can be included or excluded
- Maresha Ostracon can be included or excluded

S5.9.1 Leave-one-out statistical test

In order to test whether the model predictions are robust, we performed a leave-one-out statistical test. The 'leave-one-out' (LOO) statistical test is a resampling technique used to evaluate the performance or robustness of a statistical model. In LOO, each sample in our training data is sequentially removed (left out), and the model is then trained on the remaining data points. The left-out observation is then used to evaluate the model's performance or make predictions. This process is repeated for each observation in the training data. The goal of this statistical test is to get an indication of the variability of the model predictions by checking whether the model is overfitting the training data and the impact of outliers on the model's performance.

LOO is commonly employed when the dataset is small (which is the case for our dataset) or when it is important to assess the model's performance on each individual observation. The LOO test is a valuable tool for assessing the reliability and generalizability of a statistical model by iteratively evaluating its performance on subsets of the training data while systematically excluding each sample. Given a training set of size N, we train N models by leaving out one different data point for each model and training with the remaining $N-1$ data points. Then, on the test set, we make predictions using all N models and overlap the resulting predictions obtained from each model. This gives a visual representation of the amount of variation between the predictions of the different models.

S5.9.2 Gaussian of Gaussian

We obtain the probability and error margin for each 10-year bin from the date-prediction model. This gives us vertical Gaussian for each 10-year over the entire timeline. To convert them into a single Gaussian on the horizontal time axis, we perform a tool, dubbed 'Gaussian of Gaussian' on the predicted dates.

This program generates 1000 iterative attempts of randomly drawing a wave shape instance from the $n - bins$ distribution (over the entire timeline), assuming Gaussians (μ, σ) per bin. The max $y - peak$ position of the wave shape can be detected along the $x - axis$: For our manuscript dating problem, x represents the year value. In this manner, it becomes possible to estimate the uncertainty of peak detection in the style-based OxCal approximation, and its effect on the date estimation. This addition to the Enoch method allows to obtain an estimate of date variability, similar to the output of OxCal itself. We explored whether smoothed distribution shapes were needed for this, but a detailed analysis fortunately revealed that the method could be kept simple: Smoothing of the shape often led to an $x - axis$ shift and an increase of the x variability. The shape asymmetry of the (assumed) peak shape causes this time bias. Hence, we avoided any smoothing and *used the raw, unfiltered generated histograms*. The implicit assumption is that the 'maximum' co-occurs with a peak. Comparative plots for different information sources are obtained using the 'Gaussian of Gaussian' (see appendix S8).

References

1. Israel Antiquities Authority. The Leon Levy Dead Sea Scrolls Digital Library;. `https://www.deadseascrolls.org.il/explore-the-archive`.
2. Dhali MA, de Wit JW, Schomaker L. Binet: Degraded-manuscript binarization in diverse document textures and layouts using deep encoder-decoder networks. arXiv preprint. 2019;doi:10.48550/arXiv.1911.07930.
3. Kimball S, Mattis P. GNU Image Manipulation Program - GIMP (version 2.8.6); 2023. `https://www.gimp.org/`.

4. Mumuni A, Mumuni F. Data augmentation: A comprehensive survey of modern approaches. Array. 2022;16:100258. doi:https://doi.org/10.1016/j.array.2022.100258.

5. Bulacu M, Brink A, van der Zant T, Schomaker L. Recognition of Handwritten Numerical Fields in a Large Single-Writer Historical Collection. In: 2009 10th International Conference on Document Analysis and Recognition; 2009. p. 808–812.

6. Kohonen T. Self-organized formation of topologically correct feature maps. Biological Cybernetics. 1982;43:59–69. doi:10.1007/bf00337288.

7. Popović M, Dhali MA, Schomaker L. Artificial intelligence based writer identification generates new evidence for the unknown scribes of the Dead Sea Scrolls exemplified by the Great Isaiah Scroll (1QIsa^{a}). PLOS ONE. 2021;16:e0249769. doi:10.1371/journal.pone.0249769.

8. Joachims T. Learning to classify text using support vector machines. 2002nd ed. The Springer International Series in Engineering and Computer Science. Dordrecht: Springer; 2002.

9. Bulacu M, Schomaker L. Text-Independent Writer Identification and Verification Using Textural and Allographic Features. IEEE Transactions on Pattern Analysis and Machine Intelligence. 2007;29:701–717. doi:10.1109/tpami.2007.1009.

10. Bulacu M, Schomaker L. Combining Multiple Features for Text-Independent Writer Identification and Verification. In: Lorette G, editor. Tenth International Workshop on Frontiers in Handwriting Recognition. Université de Rennes 1. La Baule (France): Suvisoft; 2006.Available from: `https://hal.inria.fr/inria-00104189`.

11. He S, Samara P, Burgers J, Schomaker L. Image-based historical manuscript dating using contour and stroke fragments. Pattern Recognition. 2016;58:159–171. doi:10.1016/j.patcog.2016.03.032.

12. Hoerl AE, Kennard RW. Ridge Regression: Biased Estimation for Nonorthogonal Problems. Technometrics. 2000;42:80–86. doi:10.1080/00401706.2000.10485983.

13. Ramsey CB. Development of the Radiocarbon Calibration Program. Radiocarbon. 2001;43:355–363. doi:10.1017/s0033822200038212.

14. Ramsey CB, van der Plicht J, Weninger B. ‘Wiggle Matching’ Radiocarbon Dates. Radiocarbon. 2001;43:381–389. doi:10.1017/s0033822200038248.

15. Bonani G, Ivy S, Wölfli W, Broshi M, Carmi I, Strugnell J. Radiocarbon Dating of Fourteen Dead Sea Scrolls. Radiocarbon. 1992;34:843–849. doi:10.1017/s0033822200064158.

16. Jull AJT, Donahue DJ, Broshi M, Tov E. Radiocarbon Dating of Scrolls and Linen Fragments from the Judean Desert. Radiocarbon. 1995;37:11–19. doi:10.1017/s0033822200014740.

17. Hastie T, Tibshirani R, Friedman J. The elements of statistical learning: data mining, inference, and prediction. Berlin/Heidelberg: Springer Science & Business Media; 2009.

18. scikit-learn developers. scikit-learn: Bayesian Ridge Regression;. `https://scikit-learn.org/stable/modules/generated/sklearn.linear_model.BayesianRidge.html`.

19. Bishop CM. Pattern recognition and machine learning. Berlin/Heidelberg: Springer; 2006.
