# Supplementary material for: Dating ancient manuscripts using radiocarbon and AI-based writing style analysis
Source: PLoS One. 2025 Jun 4;20(6):e0323185. doi: 10.1371/journal.pone.0323185 (PMC12136314; doi:10.1371/journal.pone.0323185)
Supplement: S6 Appendix — (PDF) [file pone.0323185.s006.pdf]

S6 Appendix for the article:

Dating ancient manuscripts using radiocarbon and AI-based writing style analysis

Mladen Popović[1*], Maruf A. Dhali[1,2], Lambert Schomaker[2], Johannes van der Plicht[3], Kaare Lund Rasmussen[4], Jacopo La Nasa[5], Ilaria Degano[5], Maria Perla Colombini[5], Eibert Tigchelaar[6],

1 Qumran Institute, University of Groningen, 9712 GK, The Netherlands
2 Artificial Intelligence, Bernoulli Institute, University of Groningen, 9747 AG, The Netherlands
3 Center for Isotope Research, University of Groningen, 9747 AG, The Netherlands
4 Department of Physics, Chemistry, and Pharmacy, University of Southern Denmark, DK 5230, Denmark
5 Department of Chemistry and Industrial Chemistry, University of Pisa, 56126 Pisa PL, Italy
6 Faculty of Theology and Religious Studies, KU Leuven, 3000 Leuven, Belgium

* m.popovic@rug.nl

Data and materials: All data, code, and test film associated with this article are publicly available on Zenodo with the following DOIs:

- Data and prediction plots (v3): https://doi.org/10.5281/zenodo.10998958.
- Code and feature files (v6): https://doi.org/10.5281/zenodo.13319794.
- Film (see details in S7 Appendix: https://doi.org/10.5281/zenodo.8167946.

Please note that this article has 12 appendices in total, from **S1** to **S12**.

S6 On the use of pre-trained deep learning methods for image-based dating

A straightforward approach is to use machine-learning algorithms that are able to learn from a *small* set of labeled, i.e., dated, examples. This requirement is in conflict with the need for labeled data in current supervised deep learning, typically a thousand examples per class [1]. Recent work on Greek literary papyri uses 2,774 annotated examples from 242 dated images to train a CNN for dating task [2]. An abundance of data points is needed to warrant the stable estimation of, e.g., a neural-network model with millions of coefficients, in order to minimize the risk of arriving at a seemingly 'good' model [3]. A simple example is a linear function that has two coefficients and consequently needs a minimum of two data points to be determined. It may be appreciated that there exists a fundamental problem if the number of manuscript-date reference points is in the order of two dozen, while a computational model requires hundreds of thousands of coefficients or more. We address this challenge by applying methods that can (a) operate under sparse data conditions, (b) be explainable, and (c) not require (pre)training from an extraneous, alien image collection.

S6.1 Considerations on the use of training deep learning neural networks on a problem with only 24 examples

Since the mathematical proof by Hornik [4], it took some time but today, deep multilayer neural networks have excelled in many applications, especially since the advent of large data sets and the increase in computational power. However, as observed in the introduction of this article, the likelihood of success is low when training a deep network with too many parameters on a tiny data set. There is a serious risk of an 'overfit', i.e., a computed solution that appears to be performant on a training data set but fails to generalize (interpolate) properly when presented with unseen data [3]. We have looked at a list of 44 modern deep-learning vision models that were published since 2010 and were cited minimally 100 times. Such models have, on average, 454 million weights (coefficients) which are computed from 715 million data points in training, on average, i.e., per single model. A 'data point' is a tuple of an image and its corresponding desired model output vector for classification, regression or generative task. The meta-analysis table is kindly provided by [5, 6]. The most recent, transformer-based, models will even have billions of parameters and data points. It is evident that such large modern models can never be trained from scratch on a data set with just a few dozen, i.e., 24, radiocarbon-dated images as data points, for our problem.

An alternative approach would be the use of deep *transfer* learning [7, 8] where an existing deep neural network, trained on a sufficiently large image data set [1], is fine-tuned on a smaller set of ^{14}C-based dated images. In such a case, a hidden layer from a frozen pre-trained network is chosen as the shape-feature vector, and a new post-processing multilayer perceptron or dense network layer is trained to transform that feature vector to produce the output vector required by the actual task, for a given input image sample. We will mention five objectionable points to the use of deep transfer learning for the date prediction task.

Point 1. It is questionable whether currently common networks that are trained and designed for natural full-colour RGB photographic image classification will deliver a shape feature vector in their penultimate layer that is optimal for writer identification in bi-tonal manuscript images. Bitonal manuscript images have a flat-white background, and the interesting patterns are in the ink traces only. Such material is rarely present in generic photographic image collections. At the very least, there will be serious worries concerning efficiency, because about two-thirds of the connection weights are likely to be superfluous.

Point 2. Even if the colour-channel argument is dismissed, end users may argue that an opaque neural [1] network or vision transformer [9] method that is pretrained on non-representative image material ('photos of cats, dogs and urban scenes, etc.') would not be acceptable for answering scholarly questions. To put this in comparison, current deep foundation models are not considered a good basis for the serious

application in medical diagnostics in radiology yet, and massive data would need to be collected in order to achieve such a status [10].

Point 3. Current deep-learning methods rely on images that are often very small, i.e., 224x224 or 512x512 pixel images. Only recently, with increased memory capacity in GPUs, images of 768x768 pixels can be used. This leads to many problems in the real-world application of deep learning, e.g., in a medical context [11]. Our manuscript-image sizes are large and of variable aspect ratio, with widths of $\mu_w = 3871$ ($\pm\sigma_w = 1069$) pixels and heights of $\mu_h = 3857$ ($\pm\sigma_h = 740$) pixels. On top of the other restrictions mentioned here, using current deep learning would require a downscaling of the high-quality manuscript images with a factor of 5 to 7, with considerable loss of information. Whereas recent vision transformers [9] are better suited to deal with large images, they are based on extraneous very large image and photographic collections (cf. Point 2).

Point 4. Alternatively, tiling [12] would unnecessarily complicate the analyses because of the likely imbalance of character content between the tiles and damage to the original character appearance at the tile margins. In spite of the success and allure of deep learning, dealing with large, variable-sized images has not been fundamentally solved. This puts a limit on their applicability in several scientific domains. In microscopy [13] and astronomy [14], multi-gigapixel, terabyte images are already common. As in our case, current convolutional neural networks, per se, cannot process an original whole image in its unscaled entirety without information loss, e.g., for a prediction task.

Point 5. Regardless of the methodological problems in the face of sparse data, an end-to-end deep learning approach, i.e., transforming image pixels into a date prediction directly, has the disadvantage of limited explainability. If dedicated features can be used that are explainable and a regression model can be trained that requires limited data, such a modular approach has a distinct advantage. Still, it is worthwhile to explore a deep-learned variant for date prediction, as more (radiocarbon-)dated samples will become available.

S6.2 An attempt in using state-of-the-art deep-learning methods, PNASNet

However, in order to empirically illustrate the problems with current deep learning, even when used in a transfer-learning setup, we have used a common foundational model (PNASNet) and used its output to estimate a date probability distribution. Using a pretrained PNASNet [15], we rescaled each high-resolution manuscript image to the 'passport-photo' size, which is customary for these models, i.e., 331×331 pixels. We then used the penultimate layer ($N_{hidden} = 4320$) of this existing network as a pretrained feature vector for a date-estimation output layer with an 'OxCal' format probability target function. Figs S18 and S20 show the evolution of the loss curve in a typical training session. Although there is some variance present, the model seems to converge more or less after the presentation of 32,000 batches. However, when looking at the validation curve (Fig S19 and S21), we can see that the loss remains highly irregular. The most likely reason for this behavior is that, in spite of the frozen pre-trained mass of PNASNet, the number of fluid weights that need to be estimated for the transfer task is still in the order of 457k ($N_{hidden} = 4320$ x $N_{OxCal} = 106$). This number is irresponsibly high, in relation to the small number of images in the data set. The subpar loss level in comparison to training, the horizontality and irregularity of the validation loss curves gives us very strong support for the decision to avoid this pathway. At the very least, it can be concluded that considerable additional research would be needed to improve the DL-transfer performance, from here. Given the particular conditions of this study, we have avoided the use of deep learning for the regression task, waiting for the data sparsity to be solved.

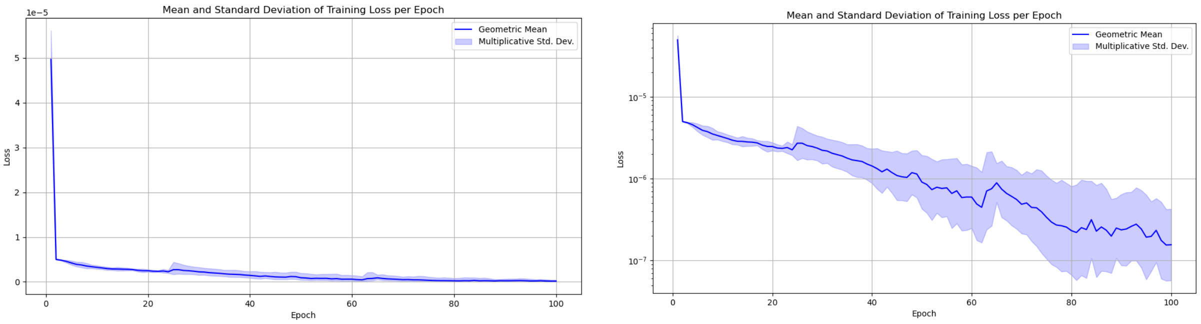

Fig S18. PNASNet training loss per epoch for 4-fold cross-validation (log-scale on the right side).

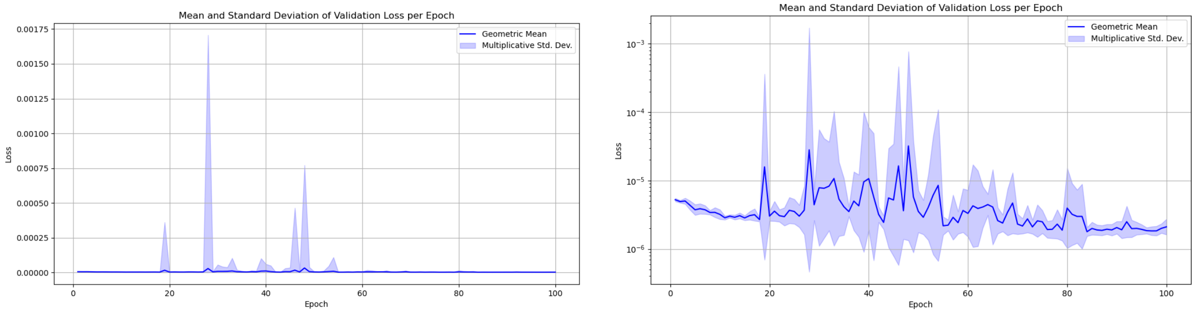

Fig S19. PNASNet validation loss per epoch for 4-fold cross-validation (log-scale on the right side).

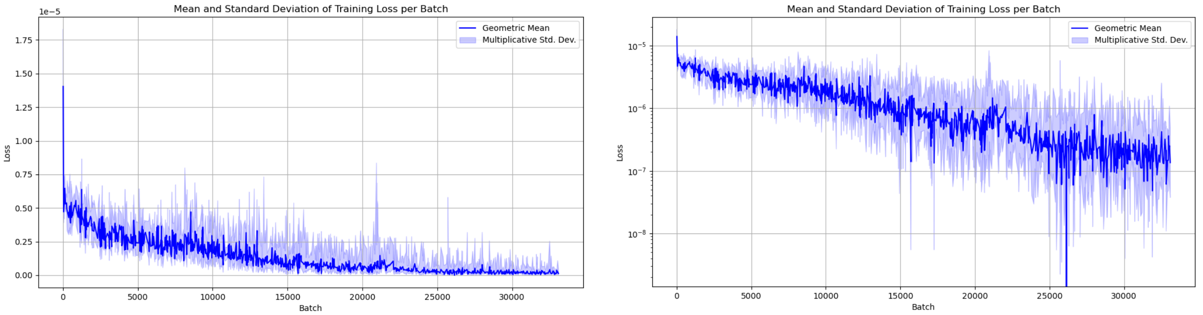

Fig S20. PNASNet training loss per batch for 4-fold cross-validation (log-scale on the right side).

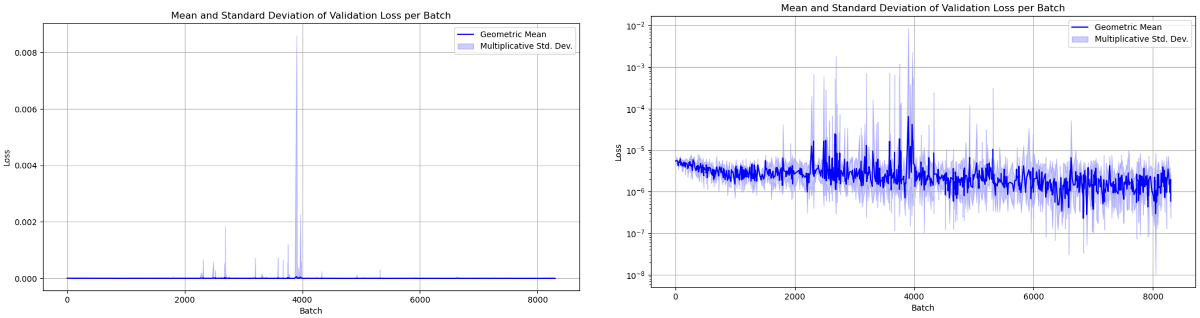

Fig S21. PNASNet validation loss per batch for 4-fold cross-validation. Validation is performed after every fourth epoch, so the curve is aligned with figure S20 (log-scale on the right side).

References

1. Krizhevsky A, Sutskever I, Hinton GE. ImageNet classification with deep convolutional neural networks. Communications of the ACM. 2017;60:84–90. doi:10.1145/3065386.

2. Pavlopoulos J, Konstantinidou M, Perdiki E, Marthot-Santaniello I, Essler H, Vardakas G, et al. Explainable dating of greek papyri images. Machine Learning. 2024;113(9):6765–6786.

3. Vapnik VN. The Nature of Statistical Learning Theory. Springer New York; 2000.

4. Hornik K, Stinchcombe M, White H. Multilayer feedforward networks are universal approximators. Neural Networks. 1989;2(5):359–366. doi:10.1016/0893-6080(89)90020-8.

5. Villalobos P, Sevilla J, Heim L, Besiroglu T, Hobbhahn M, Ho A. Will we run out of data? An analysis of the limits of scaling datasets in Machine Learning. arXiv preprint arXiv:221104325. 2022;.

6. Epoch. Parameter, Compute and Data Trends in Machine Learning; 2022. https://epochai.org/data/pcd.

7. Zhuang F, Qi Z, Duan K, Xi D, Zhu Y, Zhu H, et al. A Comprehensive Survey on Transfer Learning. Proceedings of the IEEE. 2021;109:43–76. doi:10.1109/jproc.2020.3004555.

8. Ribani R, Marengoni M. A survey of transfer learning for convolutional neural networks. In: 2019 32nd SIBGRAPI conference on graphics, patterns and images tutorials (SIBGRAPI-T). IEEE; 2019. p. 47–57.

9. Dosovitskiy A, Beyer L, Kolesnikov A, Weissenborn D, Zhai X, Unterthiner T, et al.. An Image is Worth 16x16 Words: Transformers for Image Recognition at Scale; 2020.

10. Willemink MJ, Roth HR, Sandfort V. Toward Foundational Deep Learning Models for Medical Imaging in the New Era of Transformer Networks. Radiology: Artificial Intelligence. 2022;4(6):e210284. doi:10.1148/ryai.210284.

11. Thambawita V, Strümke I, Hicks SA, Halvorsen P, Parasa S, Riegler MA. Impact of Image Resolution on Deep Learning Performance in Endoscopy Image Classification: An Experimental Study Using a Large Dataset of Endoscopic Images. Diagnostics. 2021;11(12):2183. doi:10.3390/diagnostics11122183.

12. Haja A, Schomaker LRB. A Fully Automated End-to-End Process for Fluorescence Microscopy Images of Yeast Cells: From Segmentation to Detection and Classification. In: Lecture Notes in Electrical Engineering. Springer Singapore; 2021. p. 37–46.

13. Campanella G, Hanna MG, Geneslaw L, Miraflor A, Silva VWK, Busam KJ, et al. Clinical-grade computational pathology using weakly supervised deep learning on whole slide images. Nature Medicine. 2019;25(8):1301–1309. doi:10.1038/s41591-019-0508-1.

14. Ivezić Ž, Kahn SM, Tyson JA, Abel B, Acosta E, Allsman R, et al. LSST: from science drivers to reference design and anticipated data products. The Astrophysical Journal. 2019;873(2):111.

15. Liu C, Zoph B, Neumann M, Shlens J, Hua W, Li LJ, et al. Progressive Neural Architecture Search. In: Computer Vision – ECCV 2018. Springer International Publishing; 2018. p. 19–35.
