# Supplementary material for: Dating ancient manuscripts using radiocarbon and AI-based writing style analysis
Source: PLoS One. 2025 Jun 4;20(6):e0323185. doi: 10.1371/journal.pone.0323185 (PMC12136314; doi:10.1371/journal.pone.0323185)
Supplement: S7 Appendix — (PDF) [file pone.0323185.s007.pdf]

S7 Appendix for the article:

Dating ancient manuscripts using radiocarbon and AI-based writing style analysis

Mladen Popović[1*], Maruf A. Dhali[1,2], Lambert Schomaker[2], Johannes van der Plicht[3], Kaare Lund Rasmussen[4], Jacopo La Nasa[5], Ilaria Degano[5], Maria Perla Colombini[5], Eibert Tigchelaar[6],

1 Qumran Institute, University of Groningen, 9712 GK, The Netherlands
2 Artificial Intelligence, Bernoulli Institute, University of Groningen, 9747 AG, The Netherlands
3 Center for Isotope Research, University of Groningen, 9747 AG, The Netherlands
4 Department of Physics, Chemistry, and Pharmacy, University of Southern Denmark, DK 5230, Denmark
5 Department of Chemistry and Industrial Chemistry, University of Pisa, 56126 Pisa PL, Italy
6 Faculty of Theology and Religious Studies, KU Leuven, 3000 Leuven, Belgium

* m.popovic@rug.nl

Data and materials: All data, code, and test film associated with this article are publicly available on Zenodo with the following DOIs:

- Data and prediction plots (v3): https://doi.org/10.5281/zenodo.10998958.
- Code and feature files (v6): https://doi.org/10.5281/zenodo.13319794.
- Film (see details in S7 Appendix: https://doi.org/10.5281/zenodo.8167946.

Please note that this article has 12 appendices in total, from **S1** to **S12**.

S7 Enoch's date predictions for 135 previously undated manuscripts

Before we discuss the results of the palaeographic post-hoc evaluation of the 135 unseen samples (appendix S7.4), we elaborate on the physical and image quality of the data, as well as explain how to read Enoch's prediction plots and elaborate on how Enoch differs from traditional palaeography.

S7.1 On the physical and image quality of the data

In order to appreciate how the Enoch model works, it should be noted what challenges the data pose, physically and image-wise.

As we have mentioned before, the Dead Sea Scrolls are extremely delicate material (see appendix S2.2). In a few cases, the physical evidence consists of largely intact bookrolls of several meters in length, such as the Great Isaiah Scroll (see section 4 in the main article). But in most cases, what were once large and small bookrolls are now only extant as fragmentary, deteriorated remains of various sizes and shapes. This means that the Enoch model has to deal with very diverse material remains that are available as digital images (see 2.2.1 in the main article).

The physical state of the data affects the image quality in various manners. For example, papyrus fragments often have damage patterns that affect the ink remains of the letters differently than fragments of animal skin remains do. Or, some manuscripts are represented by large, relatively well-preserved fragments, whereas others only have one small, badly damaged fragment left. Our binarized images for Enoch sometimes combine different fragments of a manuscript that are available on separate image plates of the IAA (e.g., 4Q86). Thus, the data for Enoch consists of diverse image types. Image preparation treatment is important to further improve Enoch's prediction results. The model does not change its prediction with the same set of training and testing data, but predictions can change (read "improve") because of better-cleaned images.

This also means that two or more predictions for the same manuscript can have different results because the underlying data consists of diverse image types that warrant a diverse spread in the plots. Unlike MPS [1] (or other historical manuscripts), the Dead Sea Scrolls images are all different in shape, orientation, number of characters, ink thickness, etc. Considering 4Q57, for example, there are nine "curves" (plots), which the palaeographers used in the first evaluation, because there are nine images. It should be noted that these are nine different individual images, due to improved/updated preprocessing performed over time, of 4Q57, often of different fragments. As the model receives different images (features), it produces different curves (plots). Each image represents a different set of evidence (from character shapes/features) for each bin. If all nine plots of 4Q57 were exactly the same, then that would be problematic because each image fragment is different even though they are from the same manuscript.

S7.2 How to read a prediction plot

Each prediction plot produced by Enoch presents the output of the Bayesian regression model and pertains to an individual manuscript test sample. In the plot, the X-axis delineates the chronological timeline, partitioned into 10-year bins, while the Y-axis conveys probability values in the form of means with error bars. This representation encapsulates the model's endeavour to infer the probable dating of the manuscripts, each within a 10-year interval, across a temporal expanse from 310 BCE to 200 CE years.

Within each 10-year bin, a pair of values is obtained: the mean and its corresponding error usually expressed as standard deviation. The mean is a point estimate, indicating the central tendency of the predicted manuscript dates for the given bin, thereby proposing an approximation of the most plausible date within the specific timeframe. The associated error, or standard deviation, serves as a critical metric showing the magnitude of variability inherent in the predicted dates and concurrently serves as a measure of uncertainty. An 'ideal' date prediction has a high probability and a low error.

The plot looks like a series of bars, like a histogram. By looking at these bars, we look for any patterns in the dates over time and gauge how confident or uncertain we are about these estimates. So,

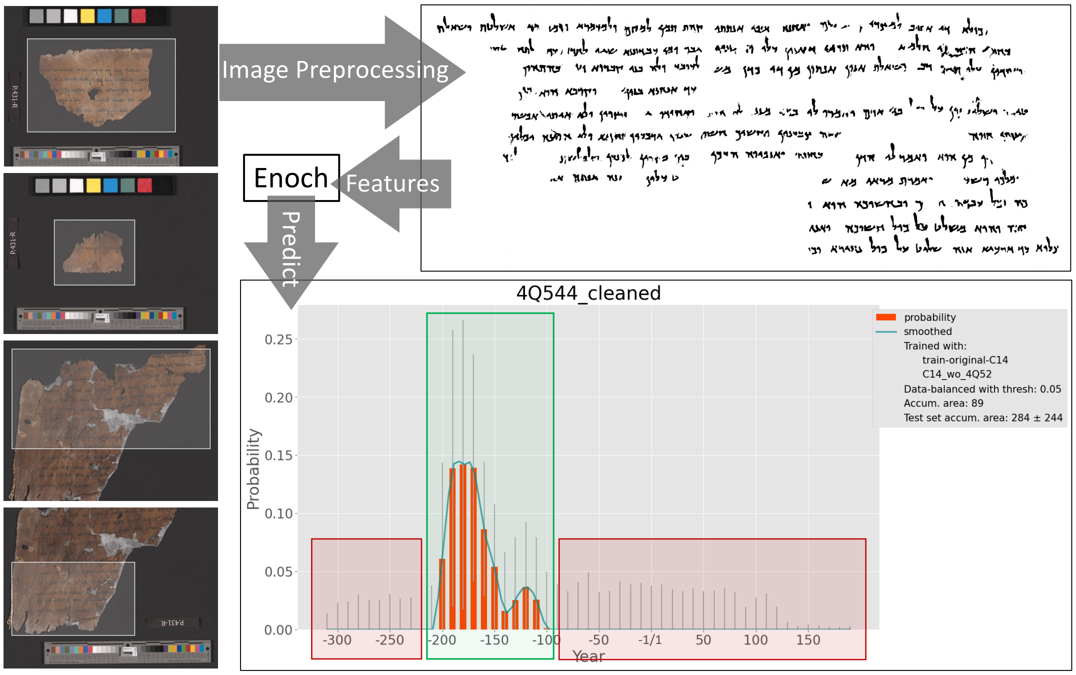

Fig S22. Enoch's prediction for 4Q544 as an example: Four fragment images from IAA plate 431 are on the left side. These fragment images are preprocessed using multispectral image fusion, neural network-based binarization (BiNet), noise reduction, and alignment correction to obtain the image on the top right. The features extracted from the preprocessed image are passed through the trained Enoch model to produce the prediction on the bottom right. This is a simple, unimodal prediction. The **green** box indicates the probable date range with high mean values (in this case, 200–100 BCE), and the **red** box indicates no mean with high uncertainty areas.

for individual plots, we look at the level of the mean value and the size of the error bars around it, to decide the most probable date or date range for that individual manuscript.

The discrete prediction bars can be mathematically smoothed into continuous curves, yielding Gaussian Mixture Models (GMMs) as a representation. This transformation allows for a more nuanced and probabilistic portrayal of the underlying distribution of predicted manuscript dates. If the smoothed prediction depicts a unimodal distribution, choosing the probable date range is easy (see Fig S22). However, it requires more attention when the prediction is bimodal. The reader then needs to pay more attention to the error bars and the means for each 10-year bin (see Fig S23). This cannot be easily solved with an algorithm: A high probability value is 'good', but not if it is accompanied by a large uncertainty. In that case, the choice of a stable estimate with a slightly lower mean probability may be advisable.

S7.3 On shared characteristics and finding matches elsewhere

In order to appreciate how the Enoch model differs from traditional palaeographic approaches, we elaborate on what was briefly mentioned in section 5 in the main article, namely that Enoch emphasizes shared characteristics and similarity matching, whereas traditional palaeography focuses on dissimilarities that are assumed indicative for style development.

Enoch's Bayesian regression model performs the quantitative analysis of textural and allographic feature vectors. These feature vectors encapsulate various handwriting characteristics, offering a systematic representation for predictive modeling. Unlike the traditional approach of human palaeographers, who often seek dissimilarities, this model employs a similarity-based strategy. It strives to uncover patterns and relationships within the feature space by quantifying the resemblance of test images to the training set with known ^{14}C date distribution. Leveraging a Bayesian framework, the model offers a probabilistic

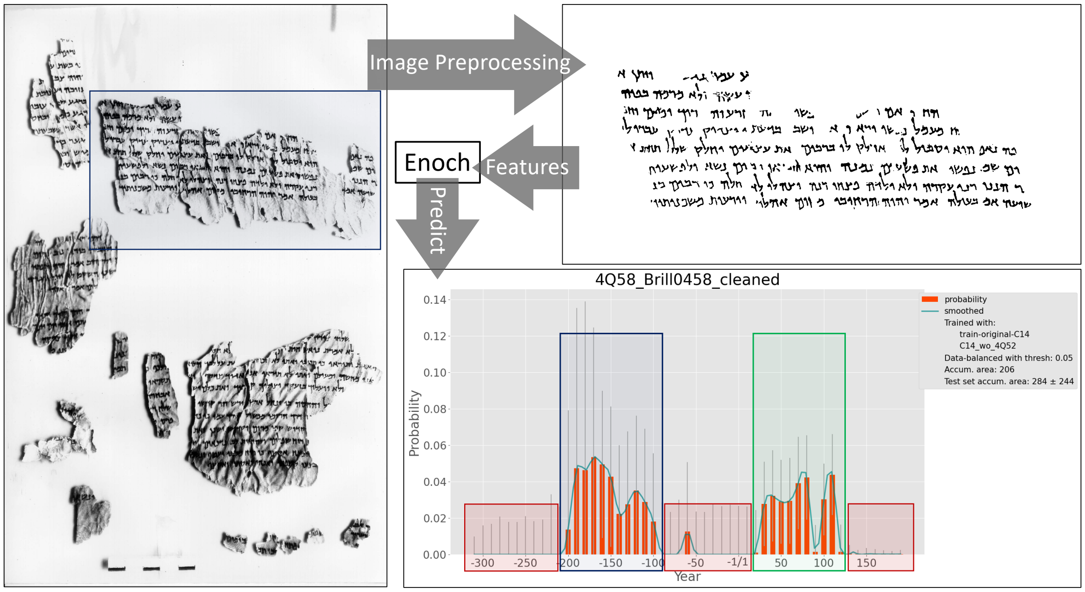

Fig S23. Enoch's prediction for 4Q58 as an example: Brill scan 431 is on the left side. The top right fragment (marked in **blue**) is preprocessed using neural network-based binarization (BiNet), noise reduction, and alignment correction to obtain the image on the top right. The features extracted from the preprocessed image are passed through the trained Enoch model to produce the prediction on the bottom right. This is a bimodal prediction. In this prediction plot, the **blue** and **green** boxes both indicate the probable date ranges with high mean values, and the **red** boxes indicate no significant mean with high uncertainty areas. Now, if the reader needs to choose one of the ranges from the **blue** or the **green**, then the **green** is the more probable range (in this case, 30– 120 CE) because of smaller error bars than the **blue** range.

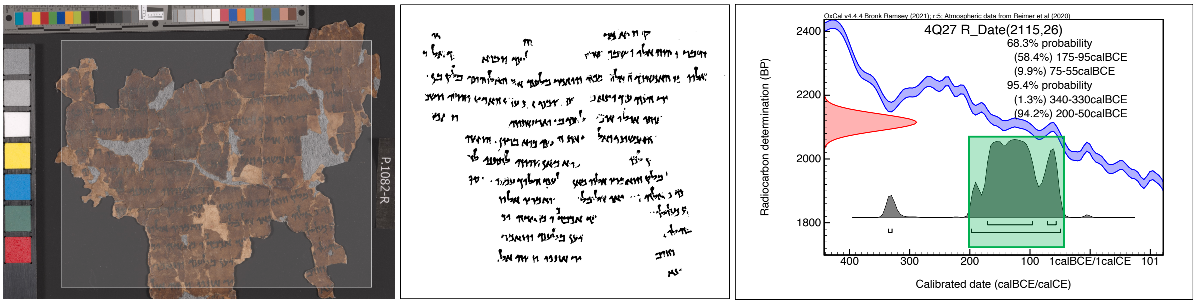

Fig S24. 4Q27 as a training sample for Enoch's date prediction (4Q27 has six images in training and two in testing. This is one of the six training images): Fragment 1 from IAA plate 1082 is on the left side. This fragment image is preprocessed using multispectral image fusion, neural network-based binarization (BiNet), noise reduction, and alignment correction to obtain the image in the middle. On the right is the OxCal data from radiocarbon dating for 4Q27, which is the target output for the training of Enoch. The **green** area indicates the accepted part of the 2σ calibrated bimodal data.

and data-driven means of attributing dates to unseen manuscripts. It thus complements the qualitative expertise of human palaeographers with a quantitative approach that can reveal subtle patterns and associations within the data. 4Q27 provides an excellent example of this approach, with six images in training Enoch and two in test prediction. Fig S24 shows one of the training images, and Fig S25 shows the prediction plot for one of the two test images.

Another example is that of 4Q319 (see section 4 and Fig 2 in the main article). Here, the AI experts'

preferred reading of the prediction plot (see Table S11) is for the younger range, against the so-called 'biased range' of 200–100 BCE. The occurrence of 'young' peaks can be seen as shape information suggesting an alternative to the bias (that is present for the 200-100 BCE range). The overall shape on the left-hand side is likely due to the OxCal-based training. However, in spite of the lesser occurrence of younger fragments in the training data, the right-hand part shows that from the style-based analysis a younger date is possible due to the stable results (with small error bars).

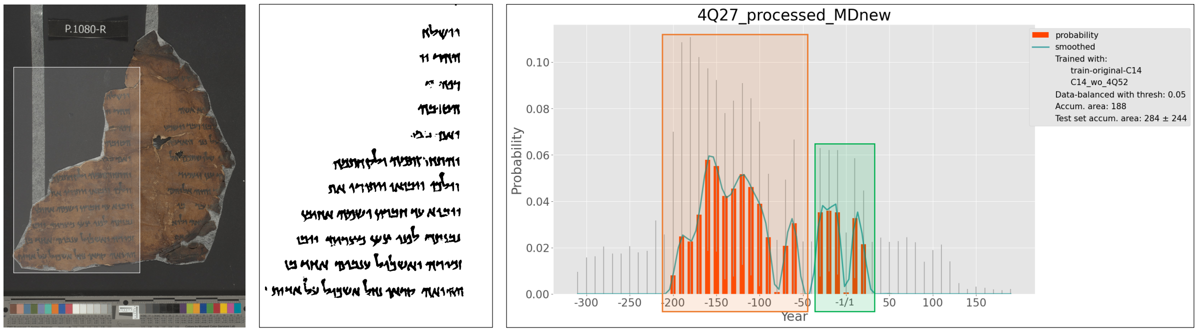

Fig S25. Enoch's prediction for 4Q27 (4Q27 has six images in training and two in testing; this is one of the two test images): Fragment 6 from IAA plate 1080 is on the left side. This fragment image is preprocessed using multispectral image fusion, neural network-based binarization (BiNet), noise reduction, and alignment correction to obtain the test image in the middle. The features extracted from the preprocessed image are passed through the trained Enoch model to produce the prediction on the right. Here, the **orange** area depicts a similar shape to the target OxCal for 4Q27 in training (see Fig S24), as expected from a regression model. In addition, Enoch also looks for similarity over all the training samples and finds an additional probability range (in **green**). Due to the low error values, i.e., smaller error bars, the **green** area is more probable (here, 30 BCE to 20 CE) than the **orange** area in this prediction plot.

S7.4 Palaeographic post-hoc evaluation

The 135 unseen samples were chosen for various palaeographic and historical reasons, such as a diachronic cross-section of a biblical book (Psalms), manuscripts that share the same writing style, or for no particular reason at all.

Second, for the evaluation, we took the best image for each manuscript in terms of image quality: better-cleaned images give better results (see appendix S7.1). The AI experts among the article's authors, M.D. and L.S., made visual evaluations of the scans in order to ensure that data is of sufficient quality with a sufficient number of characters in the used sample. The list of which specific image for each manuscript sample was used in the evaluation can be seen in Table S12. Still, there remain very poor and difficult images for a number of scrolls to work with but we kept them in the test and did not want to tweak the data. So, in cases where the best available image is still a very poor image, we worked with that (see some examples as illustrations of this very poor quality in Table S14). However, we kept all the images so the reader can see all the implications and improvements that can be obtained from careful preprocessing of the images. In Zenodo data repository (https://doi.org/10.5281/zenodo.10629480), the images are organized in three different directories: the first one with all 359 images for the 135 manuscripts, the second one with the selected 135 images, and the final one with 25 images to illustrate the poor quality of images.

Third, we did different balancing tests (see appendix S5.7) and so produced different prediction plots. Yet, in the final evaluation we only use the balanced 0.05 plots, which we also indicate in Zenodo (https://doi.org/10.5281/zenodo.10629480), in the description of *Organization of the data*. All other prediction plots are also available for readers to see the different balancing tests that we have done (see inside Enoch-predictions.tar.gz file in the Zenodo data repository).

Fourth, the AI experts, M.D. and L.S., performed a blind reading of the balanced 0.05 prediction plots. They had no knowledge of the manuscript dates and only read the prediction plots, giving estimated minimum and maximum ranges (see Table S11; for more details on how to read a plot, see appendix S7.2 and S7.3). These estimated minimum and maximum date ranges were then passed on to the palaeographers to assess the outcomes as “realistic” or “unrealistic”.

It is even possible to provide an algorithm to read the plots but the design philosophy of our date prediction model is based on the assumption that it is better to stay close to the known systematics in dealing with OxCal curves with date probabilities than to reside to an ‘oracle’ approach where an algorithm proposes a hard date range. The user can inspect the output of our Enoch model in a similar way as the OxCal curve analysis would ensue.

Again, in order to see potential suggestions by the shape-based method which are complementary to the ^{14}C-informed prediction and in order to avoid the possible criticism that the period 200–100 BCE is overrepresented in the data, the AI experts focus on additional peaks outside that zone, specifically in this exploration (see also S7.3). This means that the presence of dates in the interval 200–100 BCE is conservatively underestimated, avoiding a self-fulfilling prophecy. This does not mean that dates in this range are impossible.

Fifth, in their qualitative post-hoc evaluation, the palaeography experts among the article’s authors, M.P. and E.T., regarded a date prediction as “realistic” if a prediction corresponds (partly or wholly) with their palaeographic estimates, the basis for which was already explained in detail in appendix S1 and S4.1, and “unrealistic” when it does not. In other words, if there is an overlap between our palaeographic estimates and the machine-learning-based dating, even if the overlap is minimal, we regard the model’s date prediction as “realistic”, and “unrealistic” when there is no overlap, i.e., when it is older or younger (“too old” or “too young”). We provide our palaeographic date estimates for each of the 135 manuscripts (see Table S11), with the general principle in mind that we work with a 50-years range and allow for +/- 25 years on either side. Sometimes, e.g., in the case of quite idiosyncratic handwriting, we allow for an even broader range of 100 years. It should also be noted that if the data are of poor quality, especially if only little material is left and therefore few characters to inspect, then palaeographic estimations are more difficult to make. In other words, the palaeographic dates are not hard date ranges, but expert estimates. Still, for the evaluation we used the 50-years range in a strict sense for reasons of clarity, so that if there was only a 5- or 10-year gap we deemed the prediction as “too old” or “too young”.

Summarized, our post-hoc palaeographic assessment is based on the following considerations:

1. In line with Cross and all other palaeographers, we make a distinction between Hasmonaean- and Herodian-style writing;

2. Our palaeographic date estimates of these styles vis-à-vis one another are informed by the traditional view of the Hasmonaean script as being older than the Herodian script. Our ^{14}C results confirm for most manuscripts the basic distinction between Hasmonaean-type manuscripts that are older, and Herodian-style manuscripts that are younger. Yet, for Herodian-type script, our ^{14}C results indicate that Herodian script was present earlier than previously thought. Our evaluation of the implications of the ^{14}C data for Hasmonaean-type script provides evidence for dates in the second century BCE and also allows for the late third century BCE, and for Herodian-type script to be already in existence earlier side by side with Hasmonaean-type script in the second century BCE (see appendix S4.1.3). Thus, we took into account the general tendency in the ^{14}C results that date both individual manuscripts and the emergence of the ‘Hasmonaean’ and ‘Herodian’ scripts about 50–75 years earlier than according to traditional palaeography;

3. Linear typological developments within both Hasmonaean- and Herodian-type script have been stated by scholars, rather than substantiated with external date-bearing evidence. This makes traditional assumptions about “within script” linear typological development problematic, in our view even more so of ‘Herodian’ than of ‘Hasmonaean’. Especially for script generally seen as Late Herodian, we would not exclude a date around the turn of the era or somewhat earlier. We reckon with the possibility of a longevity of script types longer than traditionally assumed. Cross assumed a rapid development of the script from the Hasmonaean period onward. He suggested chronological

ranges of 50 years, and sometimes even shorter ranges of 25–50 years for typological developments, but these assumptions remain unsubstantiated.

It should be noted that other researchers can follow our evaluation by taking the range estimates (see Table S11) and/or look at the prediction plots (from Zenodo repository - (https://doi.org/10.5281/zenodo.8168210)), then consider the specific images of the manuscripts in question in the IAA's Leon Levy Dead Sea Scrolls Digital Library collection [2] and/or consider our binarized images (from the previously mentioned Zenodo repository), and take into account our considerations (see appendix S1 and S4.1). Or, instead of following our reasoning for a "realistic" or "unrealistic" assessment, they can make their own palaeographic post-hoc assessment, and justify their reasoning.

Also, please note that there are different probability values for each 10-year bin's prediction within these minimum and maximum ranges. So, AI experts' minimum and maximum values limit a probable range, but the range is not the final estimated date. One needs to read the probability plots to better estimate within the minimum-maximum range. This means that the range can sometimes be wide, but by reading the probability values along with the uncertainty estimates (or error bars), a reader can even narrow down to a more precise date range if they wish to do so.

The blind range estimation by the AI experts shows the distributions of year ranges in Table S10.

Table S10. Spread estimation (blind-test) by the AI expert

Range	Count	Percentage
280 years	2	1.48%
240 years	1	0.74%
210 years	4	2.96%
190 years	2	1.48%
170 years	6	4.44%
160 years	5	3.70%
150 years	5	3.70%
140 years	4	2.96%
130 years	8	5.93%
120 years	5	3.70%
110 years	8	5.93%
100 years	7	5.19%
90 years	18	13.33%
80 years	9	6.67%
70 years	4	2.96%
60 years	11	8.15%
50 years	22	16.30%
40 years	8	5.93%
30 years	5	3.70%
20 years	1	0.74%
**Total:**	**135**	**100.00%**

Some year ranges are so wide that the date prediction loses its effect of offering a limited number of quantified probability options within the time period under consideration. Fortunately, the instance of wide prediction ranges is limited within the 135 test samples. The definition for "wide range" is informed by the accepted 2σ calibrated ranges which are the training data for the Enoch model and are on average 135 years, including the so-called minor peaks, or 110 years excluding the so-called minor peaks (see Figs S14 and S15, and Table S7). Twenty-nine of the 135 test samples (21%) have a date range of more than 130 years, whereas 42 of the 135 test samples (31%) have a date range of more than 110 years.

In most cases, the date prediction range is well below 135 or 110 years, often only ca. 50 years (16%), which has the highest count of all the ranges (see Table S10).

The current average year value is 69.35 years, excluding wide ranges above 110 years, and 76.32 years, excluding wide ranges above 135 years. If one were to indiscriminately include all ranges, then the current average year value would be 98.76. The median value is 90 years. It should be noted that these average year values, as well as the median value, can change if, in the future, more manuscripts are tested. Also, if the image quality is further improved, these numbers can also be affected and improved (see below).

Most date ranges are indeed below or up to 90 years (78 out of 135 test samples). It should be noted that the possibility was claimed for the traditional palaeographic model to be able to fix a characteristic bookhand or the copying of a manuscript within 50 years or even 25–50 years, but that this was not substantiated with external date-bearing evidence (see appendix S4.1). Now, our Enoch model can produce prediction ranges of 50 years that are empirically based on physical evidence derived from ^{14}C and geometric evidence from shape-based analysis. Enoch outperforms the ^{14}C results: Enoch's predictions are even narrower than the ^{14}C date ranges in the time period 300–50 BCE, provide a more fine-grained distribution (as mentioned in section 3 in the main article).

As can be seen in Table 2 in the main article, 107 (79%) of the 135 undated manuscripts were judged to have obtained a realistic date prediction. Of course, the wider the range of years of prediction plots are, the more manuscripts show an overlap between our palaeographic estimates and the machine-learning-based dating. If we disregard the 42 date predictions with a spread wider than 110 years, then the percentage of realistic predictions drops to 50% (68 out of 135) or to 73% (68 out of 93). Thus, even with a stricter selection rule, only allowing the narrow-range estimates, still a decent percentage of palaeographically realistic evaluations can be obtained from the harvest of undated material. Moreover, if we would also take into account the image quality of the samples and choose instead not to use data of very poor quality then the performance of the Enoch model becomes even more impressive. Twenty-five images are of poor quality (see Table S13), leaving 110 images and samples in the test, of which 91% have a realistic prediction. Again, from these 110 images, if we ignore the 36 date predictions with a spread wider than 110 years, then the percentage of realistic predictions amounts to 61% out of 110, or 89% out of 74.

In the post-hoc evaluation, the palaeographers refrained from a decision in 4 cases ("see comment 1–4" in Table S11). The comments are as follows:

1. 4Q73: we consider this test sample a borderline as we would expect an older dating, ca. 100 BCE or ca. 75 BCE, in view of our considerations, especially the ^{14}C results for Hasmonaean manuscripts (appendix S7.4). The traditional palaeographic date estimation, middle of the first century BCE [3], comes close to Enoch's date prediction;

2. 4Q379: we consider the semicursive script in this manuscript difficult to date. Some semicursive manuscripts are easier to date, but this one is difficult, also according to the traditional palaeographic model there is too little to go on. Therefore, we refrain from a decision; 4Q379 could be around 100 BCE and then the prediction is realistic, but it could also be later. Cf. also [4]: the general indication "Hasmonaean semicursive" (263) indicates the difficulty in dating;

3. 4Q398: this is again a manuscript in semicursive script, and difficult to date. Other palaeography experts gave the following dates: Puech, second quarter of the first century BCE [5]; Yardeni, 50–1 BCE [6]. The prediction plot would be compatible with the latter date;

4. 4Q522: typologically, we would characterize the script as late Hasmonaean, but the date of the prediction model seems slightly too old to us. We would expect a slightly younger date, ca. 100–75 BCE, in view of our considerations (appendix S7.4). The traditional palaeographic date estimate by Puech is late Hasmonaean, second third of the first century BCE [7].

Two observations on the basis of these comments:

1. Outside nice formal bookhands, ordering Dead Sea Scrolls manuscripts according to typology can be difficult for palaeographers, especially for the semicursive script. In addition to the physical and image quality of the data (see appendix S7.1), script diversity can also pose a challenge for the Enoch model. More specifically, Enoch can handle formal and semiformal scripts well in predicting their age range, but manuscripts written in semicursive script are more difficult to date at the

current stage. This can be explained by the fact that Enoch was not yet trained enough on this (only two ^{14}C samples, 4Q114 and 4Q255/4Q433a, are in semicursive script);

2. The range 100–50 BCE is underrepresented in Enoch's date predictions. This can be explained by the distribution of ^{14}C samples across the time line, having little evidence securely fixed for this part of the time line: 4Q201, 4Q255/4Q433a, 4Q27, and 4Q2 cover (part of) the range 100–50 BCE but all of them extend beyond the range as well. Roughly speaking, Enoch predicts Hasmonaean-type manuscripts before 100 BCE and Herodian-type manuscripts after 50 BCE. Still, it should be noted that the range 100–50 BCE is not completely left devoid of Enoch's prediction plots, as the plots for 4Q185, 4Q554, and 11Q13 show, albeit with a wide range of 150 years for 4Q554.

From the machine-learning perspective, these problems can be sorted out as more samples from critical time periods are added to the training data.

S7.5 6 July 2021 test

Earlier in the project, a test was conducted on 6 July 2021. The test consisted of giving manuscripts with unseen ^{14}C results to the AI experts to see whether Enoch would give date prediction estimates that match the ^{14}C results. However, at the start of the test, it was unknown to the AI experts that the samples were chosen because of ^{14}C results being available for them afterward.

The ^{14}C results were taken from the 1990s ^{14}C dating of the Dead Sea Scrolls [8, 9]. The assumption was that the manuscripts chosen were not contaminated with castor oil as these manuscripts were not handled by the original team of editors in the 1950s [10–12]. This applies to 1QIsa^{a}, 1QpHab, 1QapGen, 1QS, 1QH^{a}, 11Q19, Mas1l.

Two more manuscripts were added for other reasons. 4Q53 was added because scholars assume that it was written by the same scribe as 1QS. 4Q319 was added because it is actually the same manuscript as 4Q259 [13], which was subjected to ^{14}C dating by our own project. It should be noted that at the time of the test this identity was not known to the AI experts.

The test was filmed. The film captures the whole process that was conducted in one go. The film can be accessed here: https://doi.org/10.5281/zenodo.8167946

Table S11. AI experts' (blind) range estimation (est_min and est_max) and palaeography experts' evaluation (pal_eval) with year range (pal_min and pal_max).

Q-num	est_min	est_max	pal_eval	pal_min	pal_max
1QapGen	-50	-1	realistic	-50	-1
1QH^{a}	-140	-1	realistic	-50	-1
1QIsa^{a}	-200	-100	realistic	-175	-125
1QpHab	-40	10	realistic	-25	25
1QS	-190	-100	realistic	-150	-100
2Q3	-40	130	realistic	-50	-1
2Q14	-310	-100	too_old	-75	-25
2Q24	-40	10	realistic	-50	-1
3Q6	-10	120	realistic	-25	25
4Q13	-40	20	realistic	-50	-1
4Q27	-30	20	realistic	-75	-25
4Q28	30	120	too_young	-200	-150
4Q38	-30	10	realistic	-50	-1
4Q38a	-190	-60	too_old	-50	-1
4Q53	-40	10	too_young	-150	-100
4Q57	-80	120	realistic	-1	50
4Q58	30	120	realistic	-1	50
4Q73	-40	10	see_comment 1	-100	-50

4Q76	-190	-150	realistic	-175	-125
4Q83	-210	-150	realistic	-175	-125
4Q84	-200	-50	realistic	-50	-1
4Q85	-140	70	realistic	25	75
4Q86	30	120	too_young	-75	-25
4Q87	-40	90	realistic	-25	25
4Q88	-190	20	realistic	-100	-50
4Q89	-50	120	realistic	25	75
4Q90	-30	-10	realistic	-75	-25
4Q91	-40	10	realistic	1	50
4Q92	-190	-100	realistic	-100	-50
4Q93	-50	30	realistic	-75	-25
4Q94	-30	20	realistic	-50	-1
4Q95	-30	90	realistic	-50	-1
4Q96	-30	10	realistic	-50	-1
4Q97	-50	10	realistic	-50	-1
4Q98	-190	-150	too_old	-50	-1
4Q98a	-40	-10	realistic	-75	-25
4Q98b	-10	120	realistic	25	75
4Q98c	10	120	realistic	25	75
4Q98f	-30	60	too_young	-100	-50
4Q98g	-200	-150	realistic	-175	-125
4Q109	-300	-240	realistic	-250	-150
4Q160	-200	-110	realistic	-175	-125
4Q161	-40	80	realistic	-50	-1
4Q163	-190	-1	realistic	-125	-75
4Q166	-40	70	realistic	-50	-1
4Q167	-50	120	realistic	-50	-1
4Q171	-50	70	realistic	-50	-1
4Q175	-150	-1	realistic	-150	-100
4Q184	-40	80	realistic	-50	-1
4Q185	-190	-80	realistic	-100	-50
4Q196	-190	-110	too_old	-100	-50
4Q203	-170	-60	too_old	-50	-1
4Q212	-100	30	realistic	-75	-25
4Q215	-30	70	realistic	-50	-1
4Q215a	-30	20	realistic	-50	-1
4Q216	-190	-110	too_old	-75	-25
4Q227	-40	30	realistic	-50	-1
4Q252	-40	20	realistic	-50	-1
4Q256	-40	10	realistic	-75	-25
4Q258	-50	20	realistic	-50	-1
4Q266	-190	-100	realistic	-100	-50
4Q267	-170	20	realistic	-50	-1
4Q271	-40	20	realistic	-75	-25
4Q272	-50	10	realistic	-75	-25
4Q274	-170	-40	realistic	-75	-25
4Q276	-30	60	realistic	-75	-25
4Q277	-30	110	realistic	-75	-25
4Q284a	-160	80	realistic	-50	-1

4Q301	-40	-1	realistic	-75	-25
4Q303	-30	110	realistic	-50	-1
4Q319 [1]	-70	-60	realistic	-125	-25
4Q325	-40	40	realistic	-50	-1
4Q373	-300	-240	too_old	-100	-50
4Q375	-30	20	realistic	-50	-1
4Q379	-190	-100	see_comment 2	-125	-75
4Q390	-30	70	realistic	-75	-25
4Q391	-200	-110	realistic	-125	-75
4Q394	-200	-110	too_old	-100	-1
4Q397	-40	60	realistic	-50	-1
4Q398	-30	10	see_comment 3	-75	-25
4Q409	-30	20	realistic	-50	-1
4Q410	-40	80	realistic	-50	-1
4Q422	-190	-110	realistic	-150	-100
4Q426	-190	-50	realistic	-100	-50
4Q431	-30	120	realistic	-50	-1
4Q432	-160	120	realistic	-50	-1
4Q434	-30	20	realistic	-75	-25
4Q436	-30	20	realistic	-50	-1
4Q437	-40	20	realistic	-50	-1
4Q439	-40	70	realistic	-25	25
4Q442	-10	120	realistic	-50	-1
4Q448	-30	10	too_young	-100	-50
4Q457	-40	-10	too_young	-150	-100
4Q471a	-160	20	realistic	-50	-1
4Q473	-30	20	realistic	-50	-1
4Q474	-30	20	realistic	-50	-1
4Q475	-30	120	realistic	-50	-1
4Q476	-30	120	realistic	-50	-1
4Q492	30	120	too_young	-75	-25
4Q493	-40	70	realistic	-75	-25
4Q494	-30	20	realistic	-50	-1
4Q501	-30	20	realistic	-75	-25
4Q508	-190	-110	too_old	-50	-1
4Q511	-30	80	realistic	-50	-1
4Q522	-200	-110	see_comment 4	-100	-50
4Q525	-50	10	realistic	-50	-1
4Q530	-30	10	too_young	-125	-75
4Q531	-30	20	realistic	-50	-1
4Q540	-40	30	too_young	-150	-100
4Q542	-50	20	too_young	-125	-75
4Q544	-200	-110	realistic	-150	-100
4Q545	-200	-100	realistic	-125	-75
4Q547	-200	-120	realistic	-125	-75
4Q554	-200	-50	realistic	-75	-25
4Q557	-200	-120	realistic	-150	-100

[1] Avoiding the data imbalance bias risk, the range 70 to 60 BCE was chosen for this particular sample. However, for 4Q319 there is additional evidence (see Fig. 2) that the period 200–100 BCE is likely, given the ^{14}C response for a fragment from the same manuscript, i.e., 4Q259.

4Q577	-50	10	too_young	-125	-75
5-6Hev1b	-40	120	realistic	50	100
5-6Hev45	-40	120	too_old	134	134
5Q5	-200	-150	too_old	-25	25
6Q18	-160	120	realistic	-50	-1
11Q5	10	120	realistic	25	75
11Q6	30	120	realistic	-1	50
11Q7	30	120	realistic	-1	50
11Q8	-30	20	too_old	25	75
11Q13	-80	20	realistic	-50	-1
11Q14	-40	120	realistic	-1	50
11Q18	-40	120	realistic	-50	-1
11Q19	-40	-1	realistic	-25	25
11Q20	-90	120	realistic	-25	25
Mas1e	-50	30	realistic	-1	50
Mas1f	-200	-100	too_old	25	75
MasJosh	-30	20	realistic	-50	-1
Mur88	-50	120	realistic	25	75
Nash	-200	-110	realistic	-175	-125
Sdeir1	-40	120	realistic	75	125

Table S12. List of images for each manuscript sample used in the post-hoc evaluation

**Q-number**	**Image-name**	**Q-number**	**Image-name**
1QapGen	1QapGen_4_crpcln	4Q301	4Q301_2_processed
1QH[a]	1QHa_QIrug-1668_cln...Lotte	4Q303	4Q303_processed
1QIsa[a]	1QIsaa_col02_cleaned	4Q319	4Q319_1_crpcln
1QpHab	1QpHab_Brill2307_cleaned_MD	4Q325	4Q325_processed
1QS	1Qs_QIrug-2153_cln...Lotte	4Q373	4Q373_processed
2Q3	2Q3_processed	4Q375	4Q375_processed
2Q14	2Q14_1_processed	4Q379	4Q379_1_processed
2Q24	2Q24_processed	4Q390	4Q390_processed
3Q6	3Q6_processed	4Q391	4Q391_4_processed
4Q13	4Q13_processed	4Q394	4Q394_cleaned
4Q27	4Q27_processed_MDnew	4Q397	4Q397_1_processed
4Q28	4Q28_256-1_cleaned	4Q398	4Q398_1_processed
4Q38	4Q38_processed	4Q409	4Q409_processed
4Q38a	4Q38a_processed	4Q410	4Q410_processed
4Q53	4Q53_405_part2_cleaned	4Q422	4Q422_2_processed
4Q57	4Q57_363_part1_cleaned	4Q426	4Q426_processed
4Q58	4Q58_Brill0458_cleaned	4Q431	4Q431_processed
4Q73	4Q73_1112-1_cleaned	4Q432	4Q432_processed
4Q76	4Q76_cleaned	4Q434	4Q434_processed
4Q83	4Q83_1148_part1_cleaned	4Q436	4Q436_processed
4Q84	4Q84_3_processed	4Q437	4Q437_processed
4Q85	4Q85_2_processed	4Q439	4Q439_processed
4Q86	4Q86_processed	4Q442	4Q442_processed
4Q87	4Q87_processed	4Q448	4Q448_processed
4Q88	4Q88_3_processed	4Q457	4Q457_processed
4Q89	4Q89_processed_MDnew	4Q471a	4Q471a_processed
4Q90	4Q90_processed	4Q473	4Q473_processed
4Q91	4Q91_processed	4Q474	4Q474_processed
4Q92	4Q92_processed	4Q475	4Q475_processed
4Q93	4Q93_processed	4Q476	4Q476_processed
4Q94	4Q94_processed	4Q492	4Q492_processed
4Q95	4Q95_processed	4Q493	4Q493_processed
4Q96	4Q96_processed	4Q494	4Q494_processed

4Q97	4Q97_processed	4Q501	4Q501_processed
4Q98	4Q98_processed	4Q508	4Q508_processed
4Q98a	4Q98a_processed	4Q511	4Q511_2_processed
4Q98b	4Q98b_processed	4Q522	4Q522_cleaned_2
4Q98c	4Q98c_processed	4Q525	4Q525_2_processed
4Q98f	4Q98f_processed	4Q530	4Q530_processed
4Q98g	4Q98g_processed	4Q531	4Q531_processed
4Q109	4Q109_cleaned_MDnew	4Q540	4Q540_processed
4Q160	4Q160_137plate_cleaned	4Q542	4Q542_cleaned
4Q161	4Q161_583_part2_cleaned	4Q544	4Q544_cleaned
4Q163	4Q163_584_599_cleaned	4Q545	4Q545_processed
4Q166	4Q166_4_processed	4Q547	4Q547_processed
4Q167	4Q167_processed	4Q554	4Q554_cleaned_MDcrpcln1
4Q171	4Q171_2_processed	4Q557	4Q557_processed
4Q175	4Q175_cleaned	4Q577	4Q577_processed
4Q184	4Q184_287_cleaned	5-6Hev1b	5-6Hev1b_891_cleaned
4Q185	4Q185_160_part2_cleaned	5-6Hev45	5-6Hev45_part2_cleaned
4Q196	4Q196_cleaned	5Q5	5Q5_processed
4Q203	4Q203_906_cleaned	6Q18	6Q18_processed
4Q212	4Q212_227_cleaned	11Q5	11Q5_2_processed
4Q215	4Q215_processed	11Q6	11Q6_2_processed
4Q215a	4Q215a_processed	11Q7	11Q7_2_processed
4Q216	4Q216_cleaned_1	11Q8	11Q8_processed
4Q227	4Q227_processed	11Q13	11Q13_579_part2_cleaned
4Q252	4Q252_processed	11Q14	11Q14_cleaned_1
4Q256	4Q256_907_cleaned	11Q18	11Q18_processed
4Q258	4Q258_140_part1_cleaned	11Q19	11Q19_Brill2293_cleaned
4Q266	4Q266_cleaned_MDcrp1	11Q20	11Q20_5-new
4Q267	4Q267_2_processed	Mas1e	Mas1e_cleaned
4Q271	4Q271_processed	Mas1f	Mas1f_processed
4Q272	4Q272_processed	MasJosh	MasJosh_cleaned
4Q274	4Q274_processed	Mur88	Mur88_2_crp-cln-prcsd_cleaned
4Q276	4Q276_processed	Nash	Nash-MS-OR-...-line9to15
4Q277	4Q277_processed	Sdeir1	Sedir1_984_cleaned
4Q284a	4Q284a_processed		

Table S13. List of twenty-five images of poor quality

Image-name	Image-name	Image-name
4Q540_processed	4Q53_405_part2_cleaned	4Q577_processed
4Q196_cleaned	4Q457_processed	4Q508_processed
4Q86_processed	4Q98_processed	4Q98f_processed
4Q492_processed	4Q448_processed	Mas1f_processed
4Q88_3_processed	4Q530_processed	4Q98c_processed
2Q14_1_processed	4Q98g_processed	4Q98b_processed
4Q284a_processed	5Q5_processed	4Q398_1_processed
4Q432_processed	4Q373_processed	4Q379_1_processed
6Q18_processed		

Table S14. A few examples of poor quality images (after binarization and cleaning).

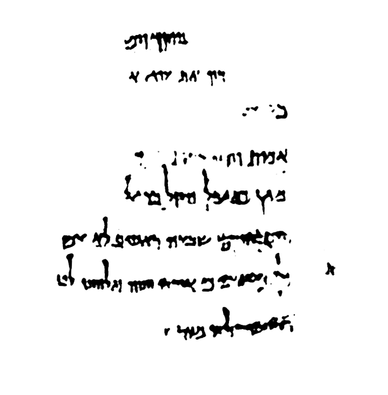	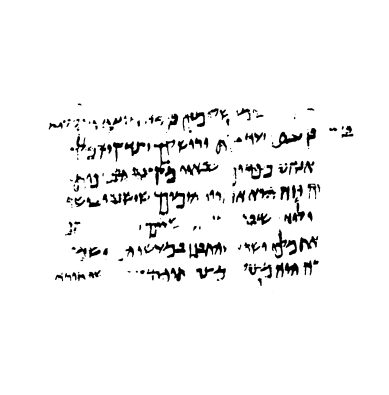
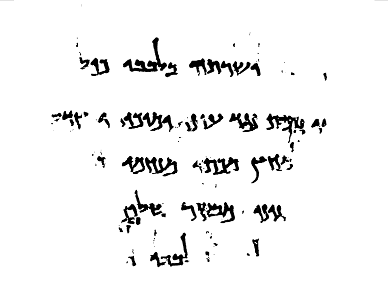	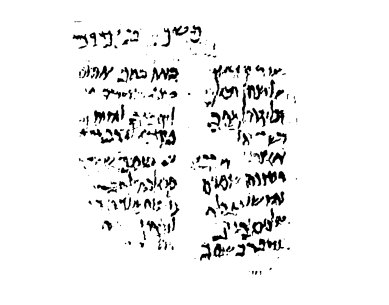
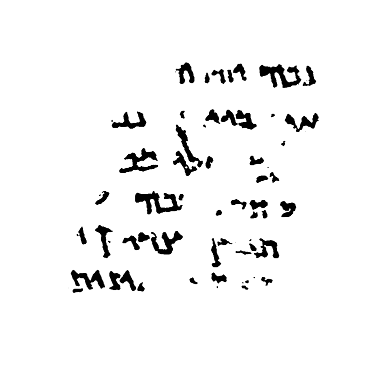	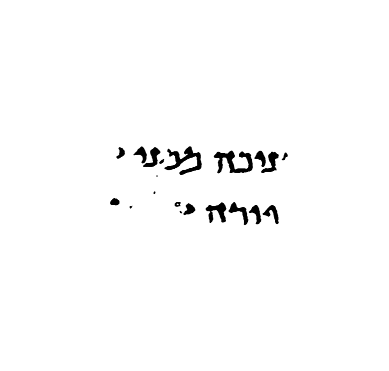
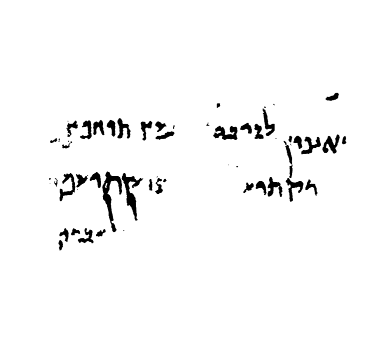	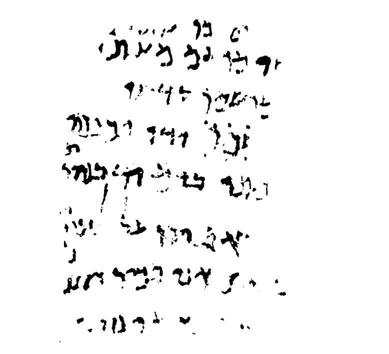

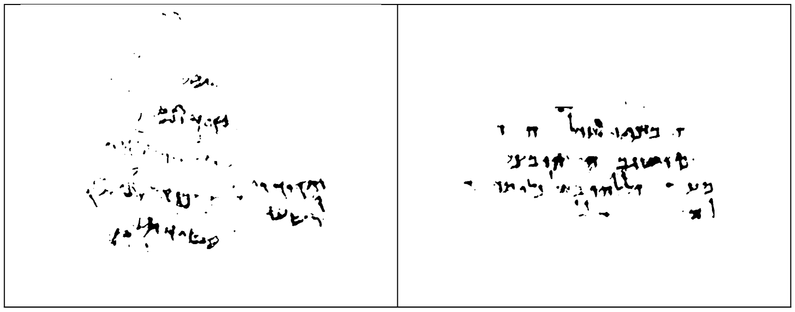

References

1. He S, Samara P, Burgers J, Schomaker L. Image-based historical manuscript dating using contour and stroke fragments. Pattern Recognition. 2016;58:159–171. doi:10.1016/j.patcog.2016.03.032.

2. Israel Antiquities Authority. The Leon Levy Dead Sea Scrolls Digital Library;. `https://www.deadseascrolls.org.il/explore-the-archive`.

3. Ulrich EC, Cross FM, Fuller RE. Discoveries in the Judaean Desert XV. Qumran Cave 4.X, The Prophets. Oxford: Clarendon Press; 1997.

4. Brooke G, Collins J, Elgvin T, Flint P, Greenfield J, Larson E, et al. Discoveries in the Judaean Desert XXII: Qumran Cave 4. XVII, Parabiblical Texts, Part 3. Oxford: Clarendon Press; 1997.

5. Puech E. La Lettre essénienne MMT dans le manuscrit 4Q397 et les parallèles. Revue de Qumran. 2015;27:99–135. doi:10.2143/RQ.27.1.3129665.

6. Qimron E, Strugnell J. Discoveries in the Judaean Desert: Volume X. Qumran Cave 4.V, Miqṣat Maʿaśe Ha-Torah. Oxford: Clarendon Press; 1994.

7. Puech E. Discoveries in the Judaean Desert: Volume XXV. Qumrân Grotte 4.XVIII. Textes hébreux: 4Q521-4Q528, 4Q576-4Q579. Oxford: Clarendon Press; 1998.

8. Bonani G, Ivy S, Wölfli W, Broshi M, Carmi I, Strugnell J. Radiocarbon Dating of Fourteen Dead Sea Scrolls. Radiocarbon. 1992;34:843–849. doi:10.1017/s0033822200064158.

9. Jull AJT, Donahue DJ, Broshi M, Tov E. Radiocarbon Dating of Scrolls and Linen Fragments from the Judean Desert. Radiocarbon. 1995;37:11–19. doi:10.1017/s0033822200014740.

10. Doudna G. Dating the Scrolls on the Basis of Radiocarbon Analysis. In: Flint PW, VanderKam JC, editors. The Dead Sea scrolls after fifty years: A comprehensive assessment, Volume one. Leiden: Brill; 1998. p. 430–471.

11. Carmi I. Are the ^{14}C Dates of the Dead Sea Scrolls affected by castor oil contamination? Radiocarbon. 2002;44:213–216. doi:10.1017/s0033822200064808.

12. Rasmussen KL, van der Plicht J, Doudna G, Nielsen F, Højrup P, Stenby EH, et al. The effects of possible contamination on the radiocarbon dating of the Dead Sea Scrolls II: empirical methods to remove castor oil and suggestions for redating. Radiocarbon. 2009;51:1005–1022. doi:10.1017/S0033822200034081.

13. Hempel C. The Community Rules from Qumran: A Commentary. Tübingen: Mohr Siebeck; 2020.
