# Supplementary material for: Dating ancient manuscripts using radiocarbon and AI-based writing style analysis
Source: PLoS One. 2025 Jun 4;20(6):e0323185. doi: 10.1371/journal.pone.0323185 (PMC12136314; doi:10.1371/journal.pone.0323185)
Supplement: S8 Appendix — (PDF) [file pone.0323185.s008.pdf]

S8 Appendix for the article:

Dating ancient manuscripts using radiocarbon and AI-based writing style analysis

Mladen Popović[1*], Maruf A. Dhali[1,2], Lambert Schomaker[2], Johannes van der Plicht[3], Kaare Lund Rasmussen[4], Jacopo La Nasa[5], Ilaria Degano[5], Maria Perla Colombini[5], Eibert Tigchelaar[6],

1 Qumran Institute, University of Groningen, 9712 GK, The Netherlands
2 Artificial Intelligence, Bernoulli Institute, University of Groningen, 9747 AG, The Netherlands
3 Center for Isotope Research, University of Groningen, 9747 AG, The Netherlands
4 Department of Physics, Chemistry, and Pharmacy, University of Southern Denmark, DK 5230, Denmark
5 Department of Chemistry and Industrial Chemistry, University of Pisa, 56126 Pisa PL, Italy
6 Faculty of Theology and Religious Studies, KU Leuven, 3000 Leuven, Belgium

* m.popovic@rug.nl

Data and materials: All data, code, and test film associated with this article are publicly available on Zenodo with the following DOIs:

- Data and prediction plots (v3): https://doi.org/10.5281/zenodo.10998958.
- Code and feature files (v6): https://doi.org/10.5281/zenodo.13319794.
- Film (see details in S7 Appendix: https://doi.org/10.5281/zenodo.8167946.

Please note that this article has 12 appendices in total, from **S1** to **S12**.

S8 Comparative plots for different information sources

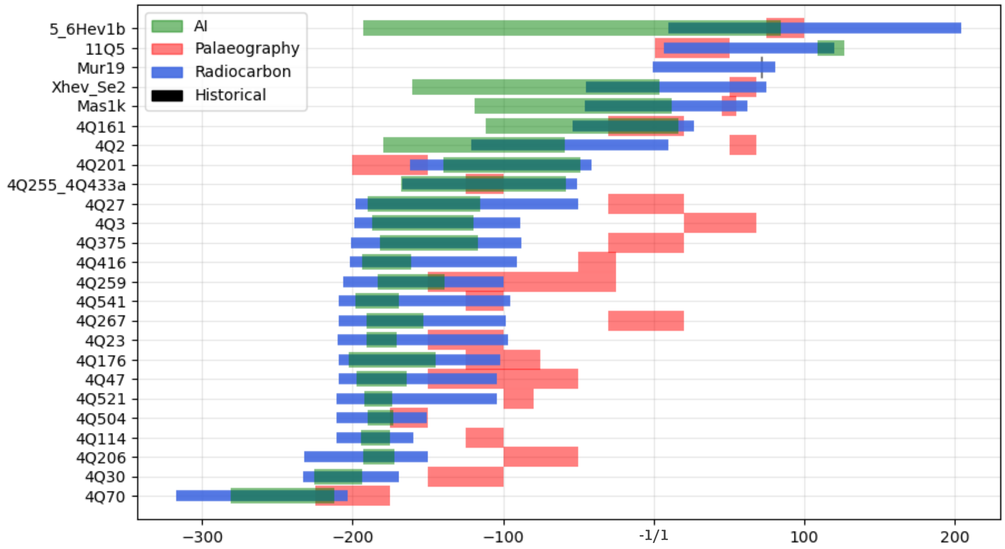

Fig S26. Overview of date estimations by three information sources and a calendar date: accepted 2σ calibrated ranges ^{14}C without minor peaks (blue), Enoch (green), palaeography (red), and historical (black). The vertical axis contains the manuscript numbers, and the horizontal axis contains dates: BCE in negative and CE in positive.

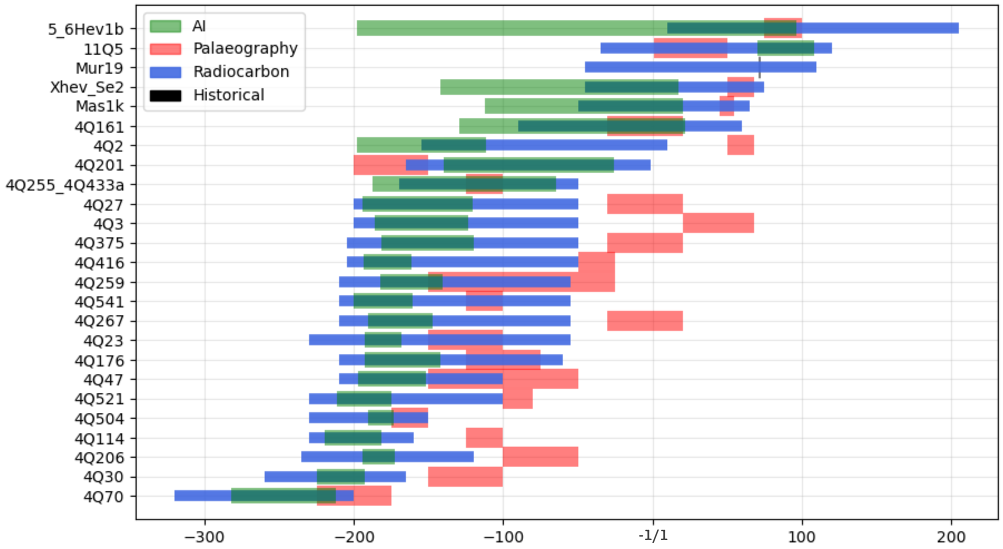

Fig S27. Overview of date estimations by three information sources and a calendar date: accepted 2σ calibrated ranges ^{14}C with minor peaks (blue), Enoch (green), palaeography (red), and historical (black). Please note that this is the same as in figure 1 in the main article, except here, the minor peaks are taken as a continuous range within the 2σ calibrated range.

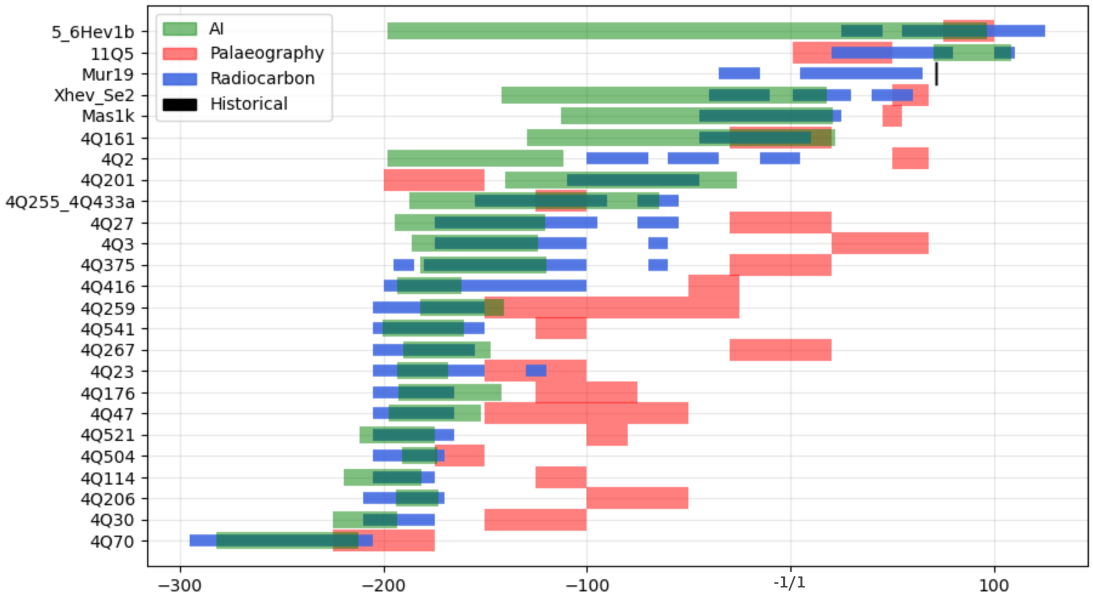

Fig S28. Overview of date estimations by three information sources and a calendar date: accepted 1σ calibrated ranges ^{14}C (blue), Enoch (green), palaeography (red), and historical (black).
