# Supplementary material for: Dating ancient manuscripts using radiocarbon and AI-based writing style analysis
Source: PLoS One. 2025 Jun 4;20(6):e0323185. doi: 10.1371/journal.pone.0323185 (PMC12136314; doi:10.1371/journal.pone.0323185)
Supplement: S9 Appendix — (PDF) [file pone.0323185.s009.pdf]

S9 Appendix for the article:

Dating ancient manuscripts using radiocarbon and AI-based writing style analysis

Mladen Popović[1*], Maruf A. Dhali[1,2], Lambert Schomaker[2], Johannes van der Plicht[3], Kaare Lund Rasmussen[4], Jacopo La Nasa[5], Ilaria Degano[5], Maria Perla Colombini[5], Eibert Tigchelaar[6],

1 Qumran Institute, University of Groningen, 9712 GK, The Netherlands
2 Artificial Intelligence, Bernoulli Institute, University of Groningen, 9747 AG, The Netherlands
3 Center for Isotope Research, University of Groningen, 9747 AG, The Netherlands
4 Department of Physics, Chemistry, and Pharmacy, University of Southern Denmark, DK 5230, Denmark
5 Department of Chemistry and Industrial Chemistry, University of Pisa, 56126 Pisa PL, Italy
6 Faculty of Theology and Religious Studies, KU Leuven, 3000 Leuven, Belgium

* m.popovic@rug.nl

Data and materials: All data, code, and test film associated with this article are publicly available on Zenodo with the following DOIs:

- Data and prediction plots (v3): https://doi.org/10.5281/zenodo.10998958.
- Code and feature files (v6): https://doi.org/10.5281/zenodo.13319794.
- Film (see details in S7 Appendix: https://doi.org/10.5281/zenodo.8167946.

Please note that this article has 12 appendices in total, from **S1** to **S12**.

S9 List of images for different tests

Table S15. Complete list of 64 training images (including 4Q52; for the date prediction model) from the radiocarbon-dated manuscripts.

Q-numbers	Name	Plate	Fragment	Q-numbers	Name	Plate	Fragment
4Q2	4Q2_1	215	1,4	4Q255	4Q255_4Q433a_1	177	3R
	4Q2_2	215	3		4Q255_4Q433a_2	177	4R
4Q3	4Q3_1	393	5	4Q259	4Q259_1	683	6
4Q23	4Q23_1	271	1,4		4Q259_2	683	7
	4Q23_2	272	5, 6, 18, 7		4Q259_3	695	1,3
	4Q23_3	272	19			696	6
		401	3, 4, 5		4Q259_4	810	3R, 5R, 7R, 8R
4Q27	4Q27_1	1080	1, 2, 6	4Q267	4Q267_1	106	2,6,9,11
	4Q27_2	1081A	2		4Q267_2	107	2,9,12
	4Q27_3	1082	1	4Q375	4Q375_1	122A	1
	4Q27_4	1082	4		4Q375_2	122A	1,2
	4Q27_5	1084B	1,7,9	4Q416	4Q416_1	180	1,2
	4Q27_6	1086B	2, 8		4Q416_2	181	1
4Q30	4Q30_1	237	7		4Q416_3	181	1
	4Q30_2	238	1	4Q504	4Q504_1	421	3, 4,5
4Q47	4Q47_1	1092	1		4Q504_2	982	1
	4Q47_2	1092	3, 5		4Q504_3	982	2
4Q52	4Q52_1	42599			4Q504_4	982	2
	4Q52_3	206	1,3	4Q521	4Q521_1	330-1	1
4Q70	4Q70_1	1109	7, 11		4Q521_2	330-1	1
	4Q70_2	1110	2	4Q541	4Q541_1	147	1,19
	4Q70_3	1110	3	5_6Hev1b	5_6Hev1b_1	890	2
		1111	1	11Q5	11Q5_1	974	1
	4Q70_4	1111	3		11Q5_2	974	1
4Q114	4Q114_1	224	1		11Q5_3	975	1
4Q161	4Q161_1	583	2,3		11Q5_4	975	1
	4Q161_2	585	2,5		11Q5_5	976	1
4Q176	4Q176_1	285	1		11Q5_6	976	3
	4Q176_2	285	2		11Q5_7	977	2
4Q201	4Q201_1	821	2		11Q5_8	978	1
		904	1		11Q5_9	979	1
	4Q201_2	821	1	Mas1k	Mas1k_1	X232	1
4Q206	4Q206_1	358	1,6		Mas1k_2	X232	1
	4Q206_2	359	1,3	Xhev_Se2	Xhev_Se2_1	534	2

Table S16. List of 135 manuscripts used for making date predictions. Please note that one manuscript may contain several images in the test dataset.

Q-numbers	Q-numbers	Q-numbers	Q-numbers
1QapGen	4Q98	4Q301	4Q508
1QH{a}	4Q98a	4Q303	4Q511
1QIsa{a}	4Q98b	4Q319	4Q522
1QpHab	4Q98c	4Q325	4Q525
1QS	4Q98f	4Q373	4Q530
2Q3	4Q98g	4Q375	4Q531

Table S16 continued from previous page

2Q14	4Q109	4Q379	4Q540
2Q24	4Q160	4Q390	4Q542
3Q6	4Q161	4Q391	4Q544
4Q13	4Q163	4Q394	4Q545
4Q27	4Q166	4Q397	4Q547
4Q28	4Q167	4Q398	4Q554
4Q38	4Q171	4Q409	4Q557
4Q38a	4Q175	4Q410	4Q577
4Q53	4Q184	4Q422	5/6Hev1b
4Q57	4Q185	4Q426	5/6Hev45
4Q58	4Q196	4Q431	5Q5
4Q73	4Q203	4Q432	6Q18
4Q76	4Q212	4Q434	11Q5
4Q83	4Q215	4Q436	11Q6
4Q84	4Q215a	4Q437	11Q7
4Q85	4Q216	4Q439	11Q8
4Q86	4Q227	4Q442	11Q13
4Q87	4Q252	4Q448	11Q14
4Q88	4Q256	4Q457	11Q18
4Q89	4Q258	4Q471a	11Q19
4Q90	4Q266	4Q473	11Q20
4Q91	4Q267	4Q474	Mas1e
4Q92	4Q271	4Q475	Mas1f
4Q93	4Q272	4Q476	Mas1l
4Q94	4Q274	4Q492	Mur88
4Q95	4Q276	4Q493	Nash
4Q96	4Q277	4Q494	Sdeir1
4Q97	4Q284a	4Q501	

Table S17. Complete list of 23 training images from a selection of previously ^{14}C-tested manuscripts [1, 2].

**Manuscript**	**Radiocarbon (BP)**	**Image IDs**
Mas1l	2086,28	MasJosh.png
1QIsa{a}	2141,32	1QIsaa_col01_cleaned.png
		1QIsaa_col02_cleaned.png
		1QIsaa_col03_cleaned.png
		1QIsaa_col34_cleaned.png
		1QIsaa_col35_cleaned.png
1QpHab	2054,22	QIrug-Qumran_extr09_2305_1QpHab_crpcln.png
		QIrug-Qumran_extr09_2306_1QpHab_crpcln.png
		QIrug-Qumran_extr09_2307_1QpHab_crpcln.png
		QIrug-Qumran_extr09_2308_1QpHab_crpcln.png
		QIrug-Qumran_extr09_2309_1QpHab_crpcln.png
		QIrug-Qumran_extr09_2310_1QpHab_crpcln.png
		QIrug-Qumran_extr09_2311_1QpHab_crpcln.png
11Q19	2030,40	QIrug-Qumran_extr09_2293_11Q19_crpcln.png
		QIrug-Qumran_extr09_2294_11Q19_crpcln.png
		QIrug-Qumran_extr09_2295_11Q19_crpcln.png

Table S17 continued from previous page

		QIrug-Qumran_extr09_2296_11Q19_crpcln.png
		QIrug-Qumran_extr09_2297_11Q19_crpcln.png
		QIrug-Qumran_extr09_2298_11Q19_crpcln.png
		QIrug-Qumran_extr09_2299_11Q19_crpcln.png
		QIrug-Qumran_extr09_2300_11Q19_crpcln.png
1QS	2041,68	QIrug-Qumran_extr09_2151_1Qs_1_crpcln.png
		QIrug-Qumran_extr09_2151_1Qs_2_crpcln.png

Table S18. Complete list of 30 images for date-bearing manuscripts from the fifth–fourth centuries BCE and the second century CE.

Manuscript	Date	Manuscript	Date
A6_11R	-411	IA06	-353
A6_12R	-411	IA17	-324
A6_13R	-411	IA21	-330
A6_14	-411	MareshaOstracon	-176
A6_15	-411	Mur24_1	133
A6_16	-411	Mur24_2	133
A6_3	-411	NS_A1r	-353
A6_4	-411	NS_A2r	-351
A6_5	-411	NS_A4r	-348
A6_7	-411	NS_A5r	-348
A6_8	-411	NS_A6r	-349
B3_1	-456	NS_C1r	-330
IA01	-348	NS_C4r	-324
IA03	-348	WDSP1_1	-335
IA04	-351	WDSP2	-352

Table S19. List of all 13 images that are split from training manuscripts and added to test.

Q-number	Number of images
4Q27	2
4Q161	2
4Q267	2
4Q375	1
5-6Hev1b	1
11Q5	5

References

1. Bonani G, Ivy S, Wölfli W, Broshi M, Carmi I, Strugnell J. Radiocarbon Dating of Fourteen Dead Sea Scrolls. Radiocarbon. 1992;34:843–849. doi:10.1017/s0033822200064158.

2. Jull AJT, Donahue DJ, Broshi M, Tov E. Radiocarbon Dating of Scrolls and Linen Fragments from the Judean Desert. Radiocarbon. 1995;37:11–19. doi:10.1017/s0033822200014740.
