# Supplementary material for: Dating ancient manuscripts using radiocarbon and AI-based writing style analysis
Source: PLoS One. 2025 Jun 4;20(6):e0323185. doi: 10.1371/journal.pone.0323185 (PMC12136314; doi:10.1371/journal.pone.0323185)
Supplement: S10 Appendix — (PDF) [file pone.0323185.s010.pdf]

S10 Appendix for the article:

Dating ancient manuscripts using radiocarbon and AI-based writing style analysis

Mladen Popović[1*], Maruf A. Dhali[1,2], Lambert Schomaker[2], Johannes van der Plicht[3], Kaare Lund Rasmussen[4], Jacopo La Nasa[5], Ilaria Degano[5], Maria Perla Colombini[5], Eibert Tigchelaar[6],

1 Qumran Institute, University of Groningen, 9712 GK, The Netherlands
2 Artificial Intelligence, Bernoulli Institute, University of Groningen, 9747 AG, The Netherlands
3 Center for Isotope Research, University of Groningen, 9747 AG, The Netherlands
4 Department of Physics, Chemistry, and Pharmacy, University of Southern Denmark, DK 5230, Denmark
5 Department of Chemistry and Industrial Chemistry, University of Pisa, 56126 Pisa PL, Italy
6 Faculty of Theology and Religious Studies, KU Leuven, 3000 Leuven, Belgium

* m.popovic@rug.nl

Data and materials: All data, code, and test film associated with this article are publicly available on Zenodo with the following DOIs:

- Data and prediction plots (v3): https://doi.org/10.5281/zenodo.10998958.
- Code and feature files (v6): https://doi.org/10.5281/zenodo.13319794.
- Film (see details in S7 Appendix: https://doi.org/10.5281/zenodo.8167946.

Please note that this article has 12 appendices in total, from **S1** to **S12**.

S10 Radiocarbon sample information

Table S20. Information of physical samples for radiocarbon dating

IAA plate	Sample	Work	Plate-fragment	Info from IAA on DJD references, places in fragments where samples were taken, previous treatments from the 1950s onward, and sample weights
206	4Q52	4QSam[b]	P206-Fr003	DJD 17: pl XXIV, fr 7; Bottom left; Maybe glues? Japanese Tissue Paper + Methylcellulose glue (2001)
421	4Q504	4QDibHam[a]	P421-Fr004	DJD 7: pl LII, fr 7; Bottom; Maybe castor oil?
285	4Q176	4QTanh	P285-Fr002	DJD 5: pl XXII, fr 10; Upper left; Scotch tape; Rice paper + Perspex glue + trichlorethylene
224	4Q114	4QDan[c]	P224-Fr001	DJD 16: pl XXXV, fr 3; Bottom right; Japanese Tissue Paper + Methylcellulose glue (2009)
385	4Q216	4QJub[a]	P385-Fr011	DJD 13: pl I, fr 12ii; Left sheet: upper margin; Scotch tape; Rice paper + Perspex glue + trichlorethylene; Hinge of Japanese Tissue Paper + Methylcellulose glue (1992); Magen Broshi sampled the right sheet and the thread in 2003, therefore it was decided not to sample it again
801	4Q185	4QSapiential Work	P801-Fr003	Not in DJD 5. Strugnell, RevQ 7 (1970) p.257 pl I, fr h. Bottom right; Japanese Tissue Paper + Methylcellulose glue (2012); Fragment 1 is sewn and encapsulated for exhibition, therefore it was decided to sample fr 3 instead
577	11Q20	11QT[b]	P577-Fr014	DJD 23: pl XLIII, fr 10b; Bottom left; Plate 608 is sewn and encapsulated for exhibition, therefore it was decided to sample plate 577 instead
64	Mur88	MurXII	P64-Fr001	DJD 2: pl LX, col X; Left sheet: bottom right; Rice paper + Perspex glue + trichlorethylene
891	5/6Hev1b	5/6HevPsalms	P891-Fr003	DJD 38: pl XXVII, fr 10; Bottom right
585	4Q161	4QpIsa[a]	P585-Fr001	DJD 5: pl IV, fr 2; Middle right; Scotch tape? Rice paper + Perspex glue + trichlorethylene
206	4Q52	4QSam[b]	P206-Fr003 (b)	DJD 17: pl XXIV, fr 7; Bottom left; Batch 1: additional material (4 mg)
285	4Q176	4QTanh	P285-Fr002 (b)	DJD 5: pl XXII, fr 10; Upper left; Batch 1: additional material (3mg)
224	4Q114	4QDan[c]	P224-Fr001 (b)	DJD 16: pl XXXV, fr 3; Bottom right; Batch 1: additional material (c.1 mg)
385	4Q216	4QJub[a]	P385-Fr011 (b)	DJD 13: pl I, fr 12ii; Left sheet: upper margin; Batch 1: additional material (4 mg)
577	11Q20	11QT[b]	P577-Fr014 (b)	DJD 23: pl XLIII, fr 10b; Bottom left; Batch 1: additional material (4 mg)
64	Mur88	MurXII	P64-Fr001 (b)	DJD 2: pl LX, col X; NB IAA did not list this one in their Excel list so no additional information
891	5/6Hev1b	5/6HevPsalms	P891-Fr003 (b)	DJD 38: pl XXVII, fr 10; Bottom right; Batch 1: additional material (6 mg)
585	4Q161	4QpIsa[a]	P585-Fr001 (b)	DJD 5: pl IX, fr 2; Middle right; Batch 1: additional material (4 mg)

Table S20 continued from previous page

1111	4Q70	4QJer[a]	P1111-Fr010	DJD 15: pl XXIX, fr 37; Left margin, middle; IAA measurement: 7 mg
1093	4Q47	4QJosh[a]	P1093-Fr005	DJD 14: pl XXXIV, fr 20; Up right diagonal margin; IAA measurement: 7 mg in two pieces
271	4Q23	4QLevNum[a]	P271-Fr002	DJD 12: pl XXIII, fr 1; Bottom margin, middle; IAA measurement: 8.50 mg (one piece, broke down into 3 after weighing)
177	4Q255/ 4Q433a	4QpapS[a]/ 4QpapHodayot-like Text B	P177 recto-Fr001	DJD 29: pl XV, fr. 1; Bottom margin, middle; IAA measurement: 6 mg in three pieces
977	11Q5	11QPs[a]	P977-Fr004	DJD 4: pl III, fr A,B,C I; Middle-left, the sample was taken from the delaminated area adjacent to the Tetragrammaton and כול; IAA measurement: 7 mg in two pieces
393	4Q3	4QGen[c]	P393-Fr005	DJD 12: pl IX; Bottom margin, right side; IAA measurement: 8-9 mg in two pieces
1081A	4Q27	4QNum[b]	P1081A-Fr002	DJD 12: pl XXXIX, fr 12; Lateral margin, bottom right; IAA measurement: 10 mg in one piece
x232	Mas1k	MasShirShabb	Px232-Fr001	Masada 6: ill 15; Bottom margin, right side; IAA measurement: 8 mg in two pieces
386	4Q206	4QEn[e] ar	P386-Fr001	Milik, Books of Enoch: pl XX, fr b; Bottom, center-left, below last א; IAA measurements: 7 mg in two pieces
237	4Q30	4QDeut[c]	P237-Fr007	DJD 14: pl V, fr 32; Bottom, center; IAA measurements: 8 mg in two pieces
904	4Q201/ 4Q338	4QEn[a] ar/ 4QGenealogical List	P904-Fr009	DJD 36: pl I, fr 2; Bottom, right; IAA measurements: 7-8 mg in two pieces
810	4Q259	4QS[e]	P810-Fr011	DJD 26: pl XV, fr 2b; Bottom; IAA measurements: 9 mg in two pieces
180	4Q416	4QInstruction[b]	P180-Fr004	DJD 34: pl VI, fr 4; Bottom, left corner; IAA measurements: 8-9 mg in two pieces
215	4Q2	Gen[b]	P215-Fr004	DJD 12: pl VI; Right blank margin, bottom left; IAA measurements: 9 mg in one piece
122A	4Q375	4QapocrMoses[a]	P122A-Fr001	DJD 19: pl XIV, fr 1; Bottom left, middle of 2nd column; IAA measurements: 8-9 mg in one piece
534	XHev/Se2	XHev/Se Num[b]	P534-Fr002	DJD 38: pl XXIX, fr 2; Bottom right corner; IAA measurements: 9 mg in two pieces
147	4Q541	4QapocrLevi[b]	P147-Frag019	DJD 31: pl XIV, fr 24; Bottom left corner; IAA measurements: 8-9 mg in one piece
330	4Q521	4QMessianic Apocalypse	P330-Fr004	DJD 25: pl III, fr 10; Bottom left corner; IAA measurements: 8 mg in two pieces
107	4Q267	4QDamascus[b]	P107-Fr010	DJD 18: pl XX, fr 9; Top left corner; IAA measurements: 7 mg in three pieces and some dust
879	Mur19	Mur pap WrDiv	P879-Fr001	DJD 2: pl XXX, fr 19; Top left corner; IAA measurements: 8 mg in one piece and some dust
