# Supplementary material for: Dating ancient manuscripts using radiocarbon and AI-based writing style analysis
Source: PLoS One. 2025 Jun 4;20(6):e0323185. doi: 10.1371/journal.pone.0323185 (PMC12136314; doi:10.1371/journal.pone.0323185)
Supplement: S11 Appendix — (PDF) [file pone.0323185.s011.pdf]

S11 Appendix for the article:

Dating ancient manuscripts using radiocarbon and AI-based writing style analysis

Mladen Popović[1*], Maruf A. Dhali[1,2], Lambert Schomaker[2], Johannes van der Plicht[3], Kaare Lund Rasmussen[4], Jacopo La Nasa[5], Ilaria Degano[5], Maria Perla Colombini[5], Eibert Tigchelaar[6],

1 Qumran Institute, University of Groningen, 9712 GK, The Netherlands
2 Artificial Intelligence, Bernoulli Institute, University of Groningen, 9747 AG, The Netherlands
3 Center for Isotope Research, University of Groningen, 9747 AG, The Netherlands
4 Department of Physics, Chemistry, and Pharmacy, University of Southern Denmark, DK 5230, Denmark
5 Department of Chemistry and Industrial Chemistry, University of Pisa, 56126 Pisa PL, Italy
6 Faculty of Theology and Religious Studies, KU Leuven, 3000 Leuven, Belgium

* m.popovic@rug.nl

Data and materials: All data, code, and test film associated with this article are publicly available on Zenodo with the following DOIs:

- Data and prediction plots (v3): https://doi.org/10.5281/zenodo.10998958.
- Code and feature files (v6): https://doi.org/10.5281/zenodo.13319794.
- Film (see details in S7 Appendix: https://doi.org/10.5281/zenodo.8167946.

Please note that this article has 12 appendices in total, from **S1** to **S12**.

S11 Data-sheet radiocarbon runs

The samples were dated by two different AMS machines characterised by codes GrA and GrM. For the GrA machine the $\delta^{13}C$ values of the IRMS are shown in the table; for GrM these are AMS values.

Table S21. Data-sheet ^{14}C runs

fragment	KLR	samplenr	GrA	GrM	^{14}a %	σ	δ^{13}C ‰	C%	<^{14}a>	<σ>	age BP	σ	remarks	used	calibrated 1σ	calibrated 2σ
P206-Fr003	11089	65369	69793		75.69	0.39	-21.22	39.5								
(4Q52)		67017		10677	74.38	0.42	-21.20									
		67017		10678	75.05	0.43	-21.40									
									75.69	0.39	2237	41	1 GrA only			
									74.71	0.30	2342	33	2 GrM averaged			
									75.07	0.24	2303	26	3 averaged	X	401–369 BCE	407–356,<br>281–232 BCE
P421-Fr004	11090	65370	68446		76.20	0.32	-17.85	48.8								
(4Q504)		65370	68447		76.43	0.33	-18.30	46.3								
		65370	68446		76.68	0.31										
		65370	68447		76.19	0.31										
									76.38	0.16	2164	16	4 averaged	X	342–321,<br>201–175 BCE	352–287,<br>228–219,<br>211-151 BCE
P285-Fr002	11091	65371	69794		77.43	0.39	-22.19	44.2								
(4Q176)		65371	69794		77.06	0.36										
		65371	69794		76.20	0.43										
		67018		10679	75.68	0.42	-21.70									
		67018		10680	75.74	0.41	-22.10									
									76.95	0.23	2104	23	3 GrA averaged			
									75.71	0.29	2235	31	2 GrM averaged			
									76.49	0.18	2153	19	5 averaged	X	343–321,<br>202–166 BCE	351–304,<br>209–102,<br>67–60 BCE
P224-Fr001	11092	65372	69795		76.04	0.39	-20.50	42.7								
(4Q114)		65372	69795		76.39	0.36										
		65372	69795		76.14	0.43										
									76.21	0.23	2182	24	3 GrA averaged			
P224-Fr001		69074		13252	76.49	0.44	-19.80						2nd run, cleaned;			
(4Q114)		69074		13253	76.73	0.39	-19.70						after soxhlet;			
		69074		13254	76.20	0.36	-20.30						no glue;			
		69074		13255	76.40	0.35	-21.00						no black spot.			
									76.44	0.19	2158	20	4 GrM averaged			
									76.34	0.15	2168	15	all 7 averaged	X	343–320,<br>202–176 BCE	352–287,<br>228–219,<br>211–160 BCE
P385-Fr011	11093	65373	69799		74.71	0.48	-21.61	46.0	74.71	0.48	2342	51	questionable ??			
(4Q216)		67020		10675	68.75	0.40	-21.70									
		67020		10676	69.24	0.38	-22.10									

Table S21. Data-sheet ^{14}C runs

**fragment**	**KLR**	**samplenr**	**GrA**	**GrM**	**^{14}a %**	σ	**δ^{13}C ‰**	**C%**	**<^{14}a>**	**<σ>**	**age BP**	σ	**remarks**	**used**	**calibrated 1σ**	**calibrated 2σ**
									69.01	0.28	2979	32	2 GrM averaged			
P801-Fr003	11094	65374	68448		77.17	0.33	-20.03	43.6								
(4Q185)		65374	68449		77.29	0.33	-20.27	46.2								
		65374	68448		76.93	0.31										
		65374	68449		77.43	0.31										
									77.20	0.16	2078	17	4 averaged		107–46 BCE	159–42,<br>7–5 BCE
P577-Fr014	11095	65357	69800		77.60	0.40	-20.93	44.1								
(11Q20)		65357	69800		77.71	0.36										
		65357	69800		77.79	0.44										
		67021		10681	76.51	0.43	-22.00									
		67021		10682	75.90	0.42	-21.00									
									77.15	0.18	2084	19	5 averaged			
									77.70	0.23	2027	24	3 GrA averaged			
									76.20	0.30	2183	32	2 GrM averaged			
		67021		18827	76.42	0.33	-20.70									
		67021		18828	75.61	0.35	-20.60									
									76.02	0.24	2202	26	2 new GrM aver			
									76.08	0.19	2195	19	4 GrM averaged			
P64-Fr001	11096	65376	69806		78.22	0.39	-21.80	42.2								
(Mur88)		65376	69806		78.73	0.36										
		65376	69806		78.31	0.44										
		67022		10663	77.66	0.41	-23.00		77.66	0.41	2030	42				
		67022		10664	79.25	0.42	-21.60		79.25	0.42	1868	43				
									78.44	0.18	1950	18	5 averaged			
									78.44	0.23	1950	23	3 GrA averaged			
									78.43	0.29	1951	30	2 GrM averaged			
		67022		18829	76.90	0.33	-21.60									
		67022		18830	77.93	0.33	-20.90									
									77.44	0.24	2053	25	2 new GrM aver			
									77.83	0.18	2013	19	4 GrM averaged			
P891-Fr003	11097	65377	69807		70.89	0.37	-21.23	41.8					questionable;			
(5/6Hev1b)		65377	69807		66.83	0.33							inhomogeneity?			
		65377	69807		72.95	0.43							reject GrA			
		67023		10659	78.07	0.38	-21.40									
		67023		10660	79.06	0.40	-21.20									
									78.54	0.28	1940	28	2 GrM averaged	X	30–42,<br>59–124 CE	10–204 CE
P585-Fr001	11098	65378	69810		77.96	0.38	-21.02	40.8								
(4Q161)		65378	69810		77.61	0.36										

Table S21. Data-sheet ^{14}C runs

fragment	KLR	samplenr	GrA	GrM	^{14}a %	σ	δ^{13}C ‰	C%	<^{14}a>	<σ>	age BP	σ	remarks	used	calibrated 1σ	calibrated 2σ
		65378	69810		78.12	0.44										
		67024		10661	77.92	0.39	-22.80									
		67024		10662	76.95	0.38	-21.40									
									77.86	0.22	2010	23	3 GrA averaged			
									77.42	0.27	2055	28	2 GrM averaged			
									77.69	0.17	2028	18	5 averaged	X	45 BCE–8 CE	89–80 BCE,<br>54 BCE–27 CE,<br>48–57 CE
P1111-Fr010<br>(4Q70)	11567	67025		11151	75.92	0.35	-19.20									
		67025		11152	75.37	0.30	-17.90									
		67025		11170	75.99	0.34	-18.80									
		67025		11171	76.00	0.33	-18.50									
									75.79	0.16	2226	17	4 averaged	X	362–351,<br>295–272,<br>266–209 BCE	375–346,<br>317–203 BCE
P1093-Fr005<br>(4Q47)	11568	67026		11153	76.27	0.32	-22.00									
		67026		11154	76.41	0.32	-23.60									
		67026		11172	76.72	0.33	-21.60									
		67026		11181	77.00	0.36	-21.70						statistic failure			
									76.46	0.19	2155	19	3 averaged	X	343–320,<br>202–167 BCE	351–291,<br>209–104 BCE
P271-Fr002<br>(4Q23)	11569	67027		11155	76.46	0.35	-24.60									
		67027		11156	76.52	0.32	-24.10									
		67027		11182	77.72	0.38	-22.80						statistic failure			
		67027		11183	77.79	0.38	-23.50						statistic failure			
									76.49	0.24	2152	24	2 averaged	X	346–316,<br>204–151,<br>129–123 BCE	351–289,<br>227–220,<br>210–97,<br>71–58 BCE
P177-Fr001<br>(4Q255/<br>4Q433a)	11570	67028		11166	77.10	0.33	-10.80									
		67028		11167	76.42	0.32	-10.10									
		67028		11184	77.70	0.38	-10.60									
		67028		11185	76.93	0.35	-10.60									
									76.99	0.17	2100	17	4 averaged	X	152–94,<br>74–56 BCE	167–51 BCE
P977-Fr004<br>(11Q5)	11571	67029		11168	77.75	0.31	-21.80									
		67029		11169	78.25	0.33	-21.60									
		67029		11186	78.70	0.38	-21.00									
		67029		11187	78.91	0.49	-20.90									

Table S21. Data-sheet ^{14}C runs

fragment	KLR	samplenr	GrA	GrM	^{14}a %	σ	$\delta^{13}C$ ‰	C%	<^{14}a>	<σ>	age BP	σ	remarks	used	calibrated 1σ	calibrated 2σ
									78.27	0.18	1967	18	4 averaged	X	23–78,<br>101–107 CE	31–16 BCE,<br>7–120 CE
P393-Fr005	11924	69725		14380	77.10	0.40	-19.00									
(4Q3)		69725		14381	76.79	0.40	-18.90									
		69725		14228	76.73	0.40	-19.10									
		69725		14229	76.45	0.40	-18.60									
									76.77	0.20	2123	21	4 averaged	X	174–102,<br>67–60 BCE	339–326,<br>199–89,<br>81–53 BCE
P1081a-Fr002	11925	69228		13385	76.69	0.36	-20.90	39.9								
(4Q27)		69228		13386	76.98	0.35	-21.20	39.7								
		69228		14170	77.90	0.27	-21.90						statistic failure			
		69228		14171	77.67	0.29	-22.20						statistic failure			
									76.84	0.25	2115	26	2 averaged	X	171–97,<br>72–57 BCE	336–330,<br>198–50 BCE
Px232-Fr001	11926	69229		13387	77.66	0.37	-20.70	40.3								
(Mas1k)		69229		13388	77.70	0.36	-20.70	41.3								
		69229		14175	77.98	0.31	-22.40									
		69229		14223	78.20	0.39	-20.30									
									77.89	0.18	2007	18	4 averaged	X	41–9 BCE,<br>1 BCE–25 CE	46 BCE–62 CE
P386-Fr001	11927	69726		14382	76.52	0.40	-20.80									
(4Q206)		69726		14383	76.54	0.41	-21.10									
		69726		14230	76.12	0.40	-20.50									
		69726		14241	76.20	0.37	-21.30									
									76.34	0.20	2169	21	4 averaged	X	348–312,<br>206–171 BCE	356–281,<br>232–150,<br>131–121 BCE
P237-Fr007	11928	69727		14565	75.91	0.44	-20.70									
(4Q30)		69727		14566	76.47	0.44	-20.90									
		69727		14395	76.82	0.35	-19.90									
		69727		14242	75.61	0.38	-20.90									
		69727		14243	76.11	0.37	-20.00									
		69727		14384	77.38	0.39	-20.00						statistic failure			
									76.21	0.17	2182	18	5 averaged	X	351–295,<br>209–176 BCE	356–279,<br>256–248,<br>233–169 BCE
P904-Fr009	11929	69230		13389	76.31	0.39	-21.10									
(4Q201/		69230		13390	77.33	0.37	-21.10									

Table S21. Data-sheet ^{14}C runs

fragment	KLR	samplenr	GrA	GrM	^{14}a %	σ	δ^{13}C ‰	C%	$<^{14}a>$	$<\sigma>$	age BP	σ	remarks	used	calibrated 1σ	calibrated 2σ
4Q338)		69230		14173	77.37	0.30	-22.10									
		69230		14174	77.53	0.31	-22.20									
		69230		14172	77.82	0.29	-22.80						statistic failure			
		69230							77.21	0.17	2077	18	4 averaged	X	107–46 BCE	162–41,<br>9–2 BCE
P810-Fr011	11930	69728		14396	76.85	0.36	-18.70									
(4Q259)																
		69728		14397	76.64	0.36	-18.80									
		69728		14244	75.99	0.37	-19.20									
		69728		14245	76.63	0.38	-19.10									
									76.53	0.18	2148	19	4 averaged	X	343–322,<br>201–155 BCE	349–311,<br>206–100,<br>68–59 BCE
P180-Fr004	11931	69729		14398	76.60	0.38	-20.30									
(4Q416)		69729		14399	76.99	0.37	-21.10									
		69729		14246	76.55	0.39	-21.60									
		69729		14359	76.63	0.54	-20.30									
									76.71	0.20	2130	22	4 averaged	X	196–184,<br>179–104 BCE	343–321,<br>202–91,<br>79–54 BCE
P215-Fr004	11932	69730		14400	77.22	0.38	-20.40									
(4Q2)		69730		14401	77.69	0.36	-19.60									
		69730		14360	76.67	0.46	-19.90									
		69730		14361	77.74	0.42	-19.80									
									77.38	0.20	2059	20	4 averaged	X	97–71,<br>58–39,<br>11 BCE–2 CE	152–131 BCE,<br>121 BCE–10 CE
P122A-Fr001	11933	69731		14567	76.55	0.45	-20.80									
(4Q375)		69731		14568	76.20	0.44	-20.30									
		69731		14362	77.23	0.42	-19.00									
		69731		14363	76.93	0.43	-19.60									
									76.74	0.22	2126	23	4 averaged	X	193–189,<br>176–101,<br>67–60 BCE	342–323,<br>201–88,<br>82–53 BCE
P534-Fr002	11934	69231		13391	77.75	0.40	-19.80									
(XHev/Se2)		69231		13392	78.14	0.36	-19.80									
		69231		14224	78.10	0.39	-19.40									
		69231		14225	77.88	0.40	-18.70									

Table S21. Data-sheet ^{14}C runs

fragment	KLR	samplenr	GrA	GrM	^{14}a %	σ	δ^{13}C ‰	C%	<^{14}a>	<σ>	age BP	σ	remarks	used	calibrated 1σ	calibrated 2σ
									77.98	0.19	1998	20	4 averaged	X	38–13 BCE,<br>3–29,<br>43–59 CE	45 BCE–75 CE
P147-Fr019<br>(4Q541)	11935	69732<br>69732<br>69732<br>69732		14569<br>14570<br>14364<br>14365	76.20<br>76.55<br>76.72<br>76.64	0.43<br>0.43<br>0.42<br>0.42	-18.80<br>-18.80<br>-18.20<br>-17.60									
									76.53	0.21	2148	22	4 averaged	X	343–320,<br>202–152 BCE	351–305,<br>209–95,<br>73–57 BCE
P330-Fr004<br>(4Q521)	11936	69733<br>69733<br>69733<br>69733		14571<br>14572<br>14377<br>14366	76.26<br>76.39<br>76.00<br>77.16	0.44<br>0.45<br>0.38<br>0.43	-21.20<br>-20.90<br>-20.90<br>-20.80									
									76.43	0.21	2159	22	4 averaged	X	346–316,<br>204–165 BCE	353–286,<br>229–217,<br>211–104 BCE
P107-Fr010<br>(4Q267)	11937	69232<br>69232<br>69232<br>69232		13393<br>13394<br>14226<br>14227	76.99<br>76.62<br>76.14<br>76.16	0.36<br>0.39<br>0.40<br>0.40	-20.40<br>-20.30<br>-19.60<br>-20.30	47.6<br>38.3								
									76.51	0.19	2151	21	4 averaged	X	344–320,<br>202–157 BCE	351–294,<br>209–98,<br>70–58 BCE
P879-Fr001<br>(Mur19)	11938	69734<br>69734<br>69734<br>69734		14573<br>14574<br>14378<br>14379	77.80<br>78.15<br>78.11<br>78.23	0.44<br>0.44<br>0.40<br>0.39										
									78.08	0.21	1987	21	4 averaged	X	32–17 BCE,<br>7–64 CE	41–9 BCE,<br>1 BCE–81 CE,<br>98–110 CE
		67806		11762	0.08000	0.030	-29.60						background age			
		67460		11306	78.24	0.10	-24.92				1971	15	Netherlands<br>Roman age			
		68084		11761	77.96	0.12	-27.31				2000	15				
P224		69073		13256	74.25	0.54	-20.60				2390	60				

Table S21. Data-sheet ^{14}C runs

**fragment**	**KLR**	**samplenr**	**GrA**	**GrM**	**^{14}a %**	σ	**δ^{13}C ‰**	**C%**	**$<^{14}a>$**	**$<\sigma>$**	**age BP**	σ	**remarks**	**used**	**calibrated 1σ**	**calibrated 2σ**
(4Q114)																
P1081a (4Q27)		69093		13262	110.5	0.56	-25.71						modern age			
