# Supplementary material for: Dating ancient manuscripts using radiocarbon and AI-based writing style analysis
Source: PLoS One. 2025 Jun 4;20(6):e0323185. doi: 10.1371/journal.pone.0323185 (PMC12136314; doi:10.1371/journal.pone.0323185)
Supplement: S12 Appendix — (PDF) [file pone.0323185.s012.pdf]

S12 Appendix for the article:

Dating ancient manuscripts using radiocarbon and AI-based writing style analysis

Mladen Popović[1*], Maruf A. Dhali[1,2], Lambert Schomaker[2], Johannes van der Plicht[3], Kaare Lund Rasmussen[4], Jacopo La Nasa[5], Ilaria Degano[5], Maria Perla Colombini[5], Eibert Tigchelaar[6],

1 Qumran Institute, University of Groningen, 9712 GK, The Netherlands
2 Artificial Intelligence, Bernoulli Institute, University of Groningen, 9747 AG, The Netherlands
3 Center for Isotope Research, University of Groningen, 9747 AG, The Netherlands
4 Department of Physics, Chemistry, and Pharmacy, University of Southern Denmark, DK 5230, Denmark
5 Department of Chemistry and Industrial Chemistry, University of Pisa, 56126 Pisa PL, Italy
6 Faculty of Theology and Religious Studies, KU Leuven, 3000 Leuven, Belgium

* m.popovic@rug.nl

Data and materials: All data, code, and test film associated with this article are publicly available on Zenodo with the following DOIs:

- Data and prediction plots (v3): https://doi.org/10.5281/zenodo.10998958.
- Code and feature files (v6): https://doi.org/10.5281/zenodo.13319794.
- Film (see details in S7 Appendix: https://doi.org/10.5281/zenodo.8167946.

Please note that this article has 12 appendices in total, from **S1** to **S12**.

S12 Worksheet of comparative data for 2σ calibrated ranges and traditional palaeographic estimates

Whole or partial overlap between 2σ calibrated ranges and palaeographic estimates in 17 of the 26 valid samples: 4Q23, 4Q47, 4Q52, 4Q70, 4Q161, 4Q176, 4Q201/4Q338, 4Q255/4Q433a, 4Q259, 4Q504, 4Q521, 4Q541, 11Q5, Mas1k, Mur19, 5/6Hev1b, XHev/Se2 (see appendix S4.1.1).

1. **4Q23 (4QLevNum^{a})**

- 355–285 BCE (29.8%), 230–220 BCE (0.8%), 210–95 BCE (62.8%), 75–55 BCE (2.1%)
- DJD 12:154 (Ulrich): early Hasmonaean formal script, dating from approximately the middle or latter half of the second century BCE (150–100 BCE).

2. **4Q47 (4QJosh^{a})**

- 355–290 BCE (33.8%), 210–100 BCE (61.6%)
- DJD 14:143 (Ulrich): referring to Cross Hasmonaean formal bookhand, second half of the second century or the first half of the first century BCE (150-50 BCE).
- Puech, Revue Biblique 122/4 (2015), 482: hasmonéenne au mieux dans la première moitié du 1er s. avant J.-C. (100–50 BCE).

3. **4Q52 (4QSam^{b})**

- 410–355 BCE (78.9%), 285–230 BCE (16.6%)
- DJD 17:220 (Cross, Parry, and Saley) (ca. 250 BCE).

4. **4Q70 (4QJer^{a}))**

- 375–345 BCE (16.3%), 320–200 BCE (79.2%)
- DJD 15:150 (Tov): quoting Yardeni 1990 and Cross 1985 (Cross shifting between earlier and later dates to settle on an earlier date), the late third or early second century BCE (225–175 BCE).

5. **4Q161 (4QpIsa^{a})**

- 90–80 BCE (1.7%), 55 BCE–30 CE (92.1%), 45–60 CE (1.7%)
- Strugnell 1970 groups this manuscript with other manuscripts such as 4Q166 and 4Q171 and gives a general indication of the script as developed rustic semiformal Herodian (see also DJD 19:112). Yardeni 2007 also lists this manuscript as part of those copied by the prolific scribe she identified and dates it to the late first century BCE to the beginning of the first century CE (30 BCE–20 CE).

6. **4Q176 (4QTanh)**

- 355–300 BCE (30.5%), 210–100 BCE (64.2%), 70–60 BCE (0.7%)
- Strugnell 1970:229 and Tigchelaar RevQ 2019; “middle Hasmonaean” (ca. 125–75 BCE).

7. **4Q201/4Q338 (4QEn[a] ar/4QGenealogical List)**

- 165–40 BCE (93.6%), 10–1 BCE (1.9%)
- Milik 1976:140: first half of the second century BCE. Mixed evidence: archaic and connections with semicursive scripts of third and second centuries BCE, perhaps dependent upon the Aramaic scripts and scribal customs of northern Syria or Mesopotamia.
- Puech 2017:99: ca. 200 BCE.
- Langlois Le premier manuscrit du Livre d'Hénoch, 62–68: ca. 150 BCE.
- 200–150 BCE

8. **4Q255/4Q433a (4QpapS[a]/4QpapHodayot-like Text B)**

- 170-50 BCE (95.4%)
- DJD 26:8, 20, 24, 29 (Alexander/Vermes, following Cross): 125–100 BCE.

9. **4Q259 (4QS[e])**

- 350–310 BCE (24.3%), 210–100 BCE (69.7%), 70–55 BCE (1.4%)
- DJD 26:8, 20, 24, 133 (Alexander and Vermes, also referring to Cross): 50–25 BCE. Late Hasmonaean/Early Herodian semicursive, with mixed semicursive and semiformal features. But 4Q259 is difficult to date palaeographically. Suggestions range from 50–25 BCE (Cross), second half second century BCE, 150–100 BCE (Milik), to first half first century BCE, preferably shortly after 100 BCE, 100–75 BCE (Puech).

10. **4Q504 (4QDibHam[a])**

- 355–285 BCE (45.4%), 230–150 BCE (50.1%)
- DJD 7:137 (Baillet): "L'écriture est une calligraphie asmonéenne, qui peut dater des environs de 150 avant J.-C." Cross: 175–150 BCE.

11. **4Q521 (4QMessianic Apocalypse)**

- 355–285 BCE (38.0%), 230–100 BCE (57.5%)
- DJD 25:3–5 (Puech): formal Hasmonaean script, following Cross; first quarter of the first century BCE (100–80 BCE).

12. **4Q541 (4QapocrLevi[b] ar)**

- 355–300 BCE (24.6%), 210–95 BCE (68.2%), 75–55 BCE (2.7%)
- DJD 31:227 (Puech): Hasmonaean, to the end of the second century BCE, ca. 100 BCE; the writing is of the type of 1QS, 1QIsa[a], 4Q175, but posterior to 4Q504 (125–100 BCE).

13. **11Q5 (11QPs[a])**

- 35–15 BCE (3.3%), 5–120 CE (92.2%)
- DJD 4:6–9 (Sanders): first half of the first century CE (1–50 CE).

14. **Mas1k (ShirShabb)**

- 50 BCE–65 CE (95.4%)
- Masada 6:120 (Newsom and Yadin; Newsom HSS 27:168): developed Herodian formal hand, late Herodian formal hand, ca. 50 CE. Also: DJD 11:239.

15. **Mur19 (pap WrDiv)**

- 45 BCE–85 CE (91.5%), 95–110 CE (3.9%)
- Cursive script with internal date of 71/72 CE validates radiocarbon date. The text refers to "year 6 of Masada". See appendix S2.4.

16. **5/6Hev1b (Ps)**

- 10–205 CE (95.4%)
- DJD 38:143: late Herodian, understood as 50-68 CE. Cross: 75–100 CE.

17. **XHev/Se2 (XHev/Se Num[a])**

- 45 BCE–75 CE (95.4%)
- DJD 38:174 (Flint): late Herodian, 50–68 CE.

Older 2σ calibrated ranges in 9 of the 26 valid samples: 4Q2, 4Q3, 4Q27, 4Q30, 4Q114, 4Q206, 4Q267, 4Q375, 4Q416 (see appendix S4.1.2).

1. **4Q2 (Gen[b])**

- 155–130 BCE (5.2%), 125 BCE–10 CE (90.3%)
- DJD 12:31 (Davila): late Herodian or even post-Herodian formal hand, ca. 50–68+ CE.

2. **4Q3 (4QGen[c])**

- 340–325 BCE (3.5%), 200–50 BCE (92.0%)
- DJD 12:39 (Davila): Herodian formal hand, dating from the middle to the end of that period, ca. 20–68 CE.

3. **4Q27 (4QNum[b])**

- 340–330 BCE (1.3%), 200–50 BCE (94.2%)
- DJD 12:211 (Jastram): following Cross, early Herodian semiformal, 30 BCE–20 CE, earlier in that range.

4. **4Q30 (4QDeut[c])**

- 360–275 BCE (57.4%), 260–245 BCE (1.4%), 235–165 BCE (36.7%)
- DJD 14:15 (White Crawford): following Cross, typical Hasmonaean book hand, 150–100 BCE. But Cross 2003 gives a more narrow date of 125–100 BCE.

5. **4Q114 (4QDan[c])**

- 355–285 BCE (49.5%), 230–160 BCE (45.9%)
- DJD 16:270 (Ulrich, following Cross): late second century BCE, no more than about a half century younger than the autograph, 125–100 BCE.

6. **4Q206 (4QEn[e] ar)**

- 360–280 BCE (48.6%), 235–145 BCE (45.8%), 135–120 BCE (1.1%)
- Milik 1976:225: Hasmonaean, probably first half first century BCE, also referring to Cross 1961: p. 138, fig. 2, lines 2 (4Q30) and 3 (4Q51) and p. 149, fig. 4, lines 2 (4Q114) and 4 (4Q398), 100–50 BCE.

7. **4Q267 (4QDamascus[b])**

- 355–290 BCE (28.6%), 210–95 BCE (65.3%), 70–55 BCE (1.6%)
- DJD 18:1, 96 (Yardeni): formal early Herodian, Cross's round semiformal; connects this manuscript with 4Q397 as possibly same scribe, 30 BCE–20 CE.

8. **4Q375 (4QapocrMoses[a])**

- 345–320 BCE (6.0%), 205–50 BCE (89.5%)
- DJD 19:112 (Strugnell): early Herodian, rustic semiformal, 30 BCE–20 CE. Compare with 4Q27, 4Q161, both radiocarbon and palaeography.

9. **4Q416 (4QInstruction[b])**

- 345–320 BCE (8.0%), 205–90 BCE (78.1%), 80–50 BCE (9.4%)
- DJD 34:74–76 (Strugnell and Harrington): Herodian, between 4Q51 and 1QM, hence "in a date transitional between the late Hasmonaean and the earliest Herodian hands", and Strugnell judged the hand of 4Q416 to be earlier than the hands of 4Q415, 4Q417, and 4Q418 by some twenty-five years (76), 50–25 BCE.
