# Supplementary material for: Dating ancient manuscripts using radiocarbon and AI-based writing style analysis
Source: PLoS One. 2025 Jun 4;20(6):e0323185. doi: 10.1371/journal.pone.0323185 (PMC12136314; doi:10.1371/journal.pone.0323185)
Supplement: Attachment — Submitted filename: PLOS ONE Response Review Comments.docx [file pone.0323185.s013.docx]

**Authors’ response to the reviewers’ comments on “Dating ancient manuscripts using radiocarbon and AI-based writing style analysis”**

**Reviewer #1**: The authors' aim has been to use the geometric features derived from the writing style as vectors to construct a regression function that points to a probabilistic range that allows dating scrolls in a specific chronological range.

The training dataset has been constructed from the dating of 30 manuscripts from which 24 valid C14 dates have been obtained for training. The images of the scrolls were binarised using a variant of U-Net and used as input for the model. Of the 75 processed images, 62 were used for training and 13 for evaluation using a leave-one-out strategy, appropriate given the size of the data.

The idea of using features based on the shape of handwriting to train a supervised regression model oriented to a chronological range based on c14 dates is indeed a clever way of looking for patterns in the data using novel computational methods that has the potential to bring a greater objectivity and a higher degree of resolution to the study of historical questions in the specific period and subject under consideration and can therefore be of help to palaeographers and historians in their decision-making and contribute to historical debates.

The model is fully reproducible, resulting in a folder with tabular and graphical outputs that match those presented in the text.

Particularly the contribution of new empirical data (c14 dates) is quite relevant to the problem of accurately reconstructing historical developments from the 4th to the 2nd century BCE and fill the chronological gap between the third century BCE and the second century CE (according to the authors) .

I am not an expert on the particular subject on which the ML techniques have been applied, so my comments are limited to a critical assessment of the methods used on an archaeological problem given my experience in that field.

I have some comments which I will describe below and which lead me to suggest some minor revisions before publication:

My main concern is how much weight the model actually contributes to the conclusions drawn. Let me explain: If the model has been trained with c14 dates in a specific chronological range. Therefore, when making out-of-sample estimates, the model will only be able to give results in the chronological ranges within which it has been trained. Thus, it is possible that some of the undoubtedly interesting conclusions, such as the greater antiquity of some materials (and their historical consequences) are strictly due to the empirical data provided and not to the results of the AI approach. With a larger sample set for training, perhaps these conclusions will change?

This leads me to suggest a consideration of nuancing some of the conclusions drawn in the paper as these observed correlations between chronological ranges and features derived from writing style, while undoubtedly relevant and contributing to reducing uncertainty about the particular problem, have limitations that could be made more explicit and should serve to prevent causal conclusions.

*We are very grateful to the reviewer for their careful and appreciative assessment and for their valuable feedback.*

1. “the model will only be able to give results in the chronological ranges within which it has been trained”

*Indeed, as the reviewer states, the style-based dating has been trained on the time range of ^14^C date points (ca. 300 BCE–130 CE). This means that the greater antiquity of some of the manuscripts moves within the time span and does not go beyond it. After the Bayesian ridge regression, manuscripts from within this range will now obtain their first date estimates due to the interpolation capability of the methods. Indeed, extrapolation to earlier and later dates is bound to get unreliable if samples are too far off in time. We draw no conclusions outside the physics-based evidence range.*

*In addition, that the model produces results in the chronological ranges within which it has been trained can be explained from a palaeographic perspective. Based on palaeography and typological comparison with date-bearing Aramaic manuscripts from the fifth-fourth centuries BCE, a dating even older than the time span 300 BCE–130 BCE is unlikely. But this could be a research question for future study.*

2. “it is possible that some of undoubtedly interesting conclusions, such as the greater antiquity of some materials (and their historical consequences) are strictly due to the empirical data provided and not to the results of the AI approach”

*The model is not fully determined by the ^14^C data, as we also mention in the discussion in* ***section 5 in the main article*** *where we further discuss the question to what extent the style-based predictions are influenced by the ^14^C-based training of the model. That does not appear to be the case. In short, the model certainly contributes independently to the conclusions through its predictions for the age of individual manuscripts, because within the time range, it gives an older date for some manuscripts and not for others. That is not automatic.*

3. “With a larger sample set for training, perhaps these conclusions will change”

*If new data becomes available in the future, some of our conclusions may change but as we also say in* ***section 5 in the main article,*** *these data can be easily added to Enoch for further refinement and precision, continuously improving accuracy. The consequences of each newly added manuscript sample to the Dead Sea Scrolls ^14^C reference collection can now easily be computed using the Enoch approach. In addition, we performed additional tests with a larger ^14^C data set for training Enoch, which includes the data from Zürich and Tucson, Arizona, but did not use those dates as default training data because of the possibility of castor oil contamination that affected those results (see main article 2.1 and Appendix S2). The addition of these data shows no significant alteration to our conclusions. The Enoch model still allows users to include the older ^14^C data in their own training of the model (--train_dataset > option 2).*

4. “these observed correlations between chronological ranges and features derived from writing style, while undoubtedly relevant and contributing to reducing uncertainty about the particular problem, have limitations that could be made more explicit and should serve to prevent causal conclusions”

*In addition to what we said above concerning how chronology and features derived from writing style are related in Enoch, from palaeogeographic and forensics handwriting research it is known that writing style evolves over time (e.g., for medieval periods: He, S., Samara, P., Burgers, J., & Schomaker, L. (2016). Historical manuscript dating based on temporal pattern codebook. Computer Vision and Image Understanding, 152, 167-175.* [*https://doi.org/10.1016/j.cviu.2016.08.008*](https://doi.org/10.1016/j.cviu.2016.08.008)*).*

*A theoretical problem would be that ‘older’ styles are intentionally copied retaining the original style. In one such highly infrequent case we were able to discriminate between writers. This illustrates that shape-based pattern recognition - even if it is a less strong type of evidence than ^14^C - is useful for the characterization of manuscripts (Popović M, Dhali MA, Schomaker L (2021) Artificial intelligence based writer identification generates new evidence for the unknown scribes of the Dead Sea Scrolls exemplified by the Great Isaiah Scroll (1QIsaa). PLoS ONE 16(4): e0249769.* [*https://doi.org/10.1371/journal.pone.0249769*](https://doi.org/10.1371/journal.pone.0249769)*).*

*As an aside, it should be noted that with our method, a large number of manuscripts dating from within the selected time range can now obtain their first, writing-style-based estimates without the necessity of clipping off physical papyrus or sheepskin material. In other words, on the basis of 24 ^14^C samples, we can now compute date estimates for ~1000 manuscripts, and have computed results for 135 out-of-sample manuscripts for which palaeographic confirmation was determined.*

- Line(75) It could be dispensed with as it does not add much to the debate.

*As there is much to do about deep learning, we think it is good to signal here that in this part of our pipeline we did not apply deep learning. To clarify, we added: “****It was decided to let the available data speak, not depending on a pretrained model****”*

- (Line 167) I cant find fig 29.

*Thank you for noticing this. This was an editing mistake. It happened as we separated the main article from the supplementary material. The main article has 3 figures. The figure counting in the supplementary material should start from Figure 4, but now it starts from Figure 1. So, in the main, Figure 29 is Figure 26 in the submitted supplementary. We have updated it and renamed all the supplementary figures starting with S1. We also noticed the same editing issues with the table names. This has also been corrected now.*

- (Line 1529-1532 S7). Did the authors of the article perform the post-hoc evaluation? If so, more clarity is needed in the main text on how the assessment has been carried out, as the issue has methodological implications as it is the baseline from which the final reliability of the model is assessed when dealing with out-of-sample data and may be susceptible to confirmation bias.

*Yes, two of the article’s authors performed the post-hoc palaeographic evaluation. We say so explicitly in these lines, we give their initials in line 1530, Mladen Popović and Eibert Tigchelaar. We have now also added that these are the palaeography experts among the article’s authors in the appendix and in the main article. And explain in detail in the following lines (until 1574) on the basis of what criteria and procedure the assessment has been carried out. The experts produced their own evaluation independently. Please observe that we emphasise that the palaeographic evaluation is based on expert knowledge and we explain our reasoning and are transparent in providing our estimate for each manuscript, explicitly inviting discussion and also other experts to agree or disagree on the basis of palaeographic arguments. That is exactly the point of the tool, the model’s predictions for particular manuscripts are to be used by palaeographers to substantiate, sharpen, or modify their own subjective estimates for specific manuscripts, often to the precision of only 50-year ranges for manuscripts that are more than 2000 years old. We provide all the information in the prediction plots (and all the associated data files) so that readers can make their own decisions. The advantage is that the model provides a more objective and empirical basis for palaeographic reasoning. The ^14^C and the pattern-recognition methods together provide alternative, quantified interpretations to the opinions of palaeography experts. The purpose of the current paper is to stimulate scholarly discussion on the newly provided dates for manuscripts from the target period.*

*Please observe that the palaeographic evaluation is conducted post-hoc and does not determine the model’s final reliability. During development, we assessed the Enoch model using three cross-validation approaches: a train-validation split, manuscript-image splitting for train-test sets, and leave-one-out evaluation. This process has successfully trained the model on the ^14^C data, with its robustness and accuracy duly validated through the aforementioned assessments. The model’s predictions achieved an 85.14% overlap with the original ^14^C probability distributions (see Section 3 in the main article) and predicted style-based radiocarbon dates with mean absolute errors (MAEs) ranging from 27.9 to 30.7 years compared to actual radiocarbon dating. These results establish the model’s reliability. Only after this validation does the post-hoc palaeographic analysis occur, leveraging the model’s predictions for specific manuscripts. This post-hoc palaeographic analysis can be done by any knowledgeable palaeographer.*

- (Line 234 Main text) What are the multiple dating methods integrated in the model? As I understand it, the shape-based features derived from the writing style (geometric evidence) have been extracted, binarized and used as input to construct a supervised dataset whose response variable is a date interval (2σ) according to the set of radiocarbon dates (physical evidence) presented, and that the results have been validated against the default palaeographic method. It is not clear how other dating methods(palaeography) are integrated into the model. My concern is compounded by the statement in line (258) about avoiding palaeographic estimates as target values and the statement in line (890 S4.2) that says that ‘In this study, we combine palaeography and radiocarbon dating to train our date prediction model’. Just after, in line (924 S 4.2) it is confirmed that ‘To train our artificial intelligence-based date prediction model, we used the accepted 2σ calibrated data from 24 of the 26 valid 14 C results". In Appendix S 4.2, although the problem associated with the qualitative nature of the palaeographic method for estimating the age of the scrolls is explained, it is not made clear how this method has been integrated into the training of the model. Therefore, it seems to me necessary to better explain how palaeography has been used in the model's predictor space, if at all, or to disambiguate the different references to the dating methods used within the model, as well as to rephrase the title of S4.2.

*The multiple dating methods integrated in the model are indeed physical evidence (14C) and geometric evidence (character-based features derived from handwriting style). Only in the case of bimodal ^14^C evidence have we used palaeographic knowledge to reject part of the ^14^C input for the training of the model, using the Heaviside function to do so. Not doing so would imply a propagation of the ^14^C calibration problem to the subsequent processing stage, with unlikely predictions that can be easily avoided. We explain this in detail in Appendices S4.2 and S.5.6.2. Thus, palaeography can be said to have been used indirectly, limiting bimodal ^14^C evidence that is then used to train the model, and not directly, for example using palaeographic estimates as target values.*

*To disambiguate we have modified the title of S4.2 (>>>* ***Applying palaeography to determine usable bimodal radiocarbon data input to train the artificial intelligence-based date-prediction model****) as well as the phrasing in S4.2:*

*In this study, we combine palaeography and radiocarbon dating to train our date prediction model.*

*>>>*

***In this study, only in the case of bimodal ^14^C evidence have we used palaeographic knowledge to determine the usable radiocarbon dating input for the training of our date-prediction model (see S5.6).***

*To clarify we have also added,* ***in bold****, in* ***section 2.2*** *in the main article: .*

***In the case of bimodal evidence,*** *we used a Bayesian method outside the OxCal program to limit the date ranges produced from each calibration.* ***We did this to prevent propagation of ^14^C bimodality, which is an effect of the calibration curve not being monotonous (see Appendix S2.4), to the next processing stage of training the style-based predictor. Only in the case of bimodal ^14^C evidence was palaeographic domain knowledge used in training the Enoch model as it allows making a binary split in the OxCal distribution of 2sigma ranges using the Heaviside function with the position of the step being placed at an innocuous low-probability point, near zero, on the curve*** *(see Appendix S5.6).*

*And in* ***section 3*** *in the main article we have added,* ***in bold****: we utilize three information sources: ^14^C, shape-based writing style analysis (AI), and palaeography****, the latter only in the case of bimodal ^14^C evidence****.*

Technical comments:

Although the reproduction of the procedures presented requires a certain level of expertise for which the resources are sufficiently well presented, possible non-technically skilled stakeholders may benefit from having a static version that presents the results alongside the code.

*It is possible to check the results (prediction plots) directly without running the code. They are presented in a separate Zenodo repository. Please see Data and prediction plots (v3): https://doi.org/10.5281/zenodo.10998958.*

The inclusion of indications for the creation of a virtual environment as well as the specific version of python whitin the README file would benefit the reproducibility of the exercise.

*Thank you for the suggestion. It will be useful for future users. We have now updated the Zenodo repository of ‘Code and feature files (v6)’. The software requirement under the description also explains how to create a virtual environment using ‘Conda’ from the provided Python requirements.*

The work therefore presents in a general way novel knowledge that can contribute to advance the knowledge of the specific subject and is of general scientific and historical interest.

**Reviewer #2**: This manuscript presents a novel use of AI based on radiocarbon dating and handwriting style to determine or correct the dates estimated by the palaeographic studies of the so-called Dead Sea Scrolls. It introduces Enoch, an AI-based prediction model that the authors have trained and tested with these manuscripts, demonstrating its potential, which this reviewer understands to be of significant interest (even to be trained and used in other archaeological/historical contexts where dating based on writing analysis can be corrected).
The study presents results of an original research. With regard to materials and methods, the authors are thorough in describing the manuscript selection processes, sampling, laboratory analysis, and data treatment. Radiocarbon analysis protocols have been adapted to solve the difficulties on dating the fragile samples avoiding contamination.
The main difficulty in dating the scrolls lies in the uncertainty inherent in the two methods used, the discrepancies on writing style analysis and the limitations of the radiocarbon dating method and its calibration. The authors dedicate several pages in the supplementary material (S1) to demonstrating the contradictions of palaeography-based dating and the necessity of tools like Enoch.
The authors state that radiocarbon dates are “more reliable time markers” and “palaeographic estimates do not provide absolute or fixed dates” (lines 572-576, Appendix S4.1). In addition, they “compare the radiocarbon dates with previous palaeographic estimates only in a general sense, not as a rigid application of these estimates” (lines 520-521, Appendix S4.1). However, when working with calibrated radiocarbon dates, the selection or rejection of the C14 temporal ranges used to train Enoch are based on very specific palaeographic temporal proposals (e.g., Appendix S4.1.1). In this regard, the dependence of the C14 dating use from palaeography might appear as an important contradiction.
Concerning this, author’s arguments are better explained in Appendix S5.The statistical calculations and the functioning of Enoch are discussed in detail by the authors. The limitations of the sample size (24 radiocarbon dated scrolls) is explained too. Conclusions are supported by the data.
Finally, in addition to the 95 pages of supplementary material, the authors provide access to other data, such as the OxCal codes, raw data or a video where the rationale behind the research and the control tests conducted can be better understood.
In the opinion of this reviewer, the article should be published with only a couple of minor improvements:

1- In the case of 5/6 Hev1b, the discrepancies between the C14 results and the paleographic estimates are explained (lines 711-716, S4.1.1). However, the discrepancy between those dates and the ones proposed by Enoch, which is clearly shown in Figure 1, is not sufficiently detailed.

2- For practical reasons, table 21 should include Q-numbers.

*We are very grateful to the reviewer for their careful and appreciative assessment and for their valuable feedback.*

*For sample 5/6 Hev1b, the style-based estimate (green) appears to be uncertain, ranging over a wide period, which, however, overlaps with the ^14^C based estimates (blue). Interestingly, the palaeographic estimate (red) falls within the overlap region. We have added this to* ***section 3 in the main article****.*

*For Table 21 we have included Q-numbers.*
